# A Novel PIFA/KOH Promoted Approach to Synthesize C2-arylacylated Benzothiazoles as Potential Drug Scaffolds

**DOI:** 10.3390/molecules27030726

**Published:** 2022-01-22

**Authors:** Xiao-Tong Sun, Zhi-Gang Hu, Zhen Huang, Ling-Li Zhou, Jian-Quan Weng

**Affiliations:** College of Chemical Engineering, Zhejiang University of Technology, Hangzhou 310014, China; SXT11096@163.com (X.-T.S.); H1718619378@163.com (Z.-G.H.); zjuthuangzhen@163.com (Z.H.); zl17398375385@163.com (L.-L.Z.)

**Keywords:** *2H*-benzothiazoles, aryl methyl ketones, arylacylation, PIFA/KOH

## Abstract

To discover an efficient and convenient method to synthesize C2-arylacylated benzothiazoles as potential drug scaffolds, a novel [bis(trifluoroacetoxy)iodo]benzene(PIFA)/KOH synergistically promoted direct ring-opening C2-arylacylation reaction of *2H*-benzothiazoles with aryl methyl ketones has been developed. Various substrates were tolerated under optimized conditions affording the C2-arylacylation products in 70–95% yields for 38 examples. A plausible mechanism was also proposed based on a series of controlled experiments.

## 1. Introduction

**Figure 1:**
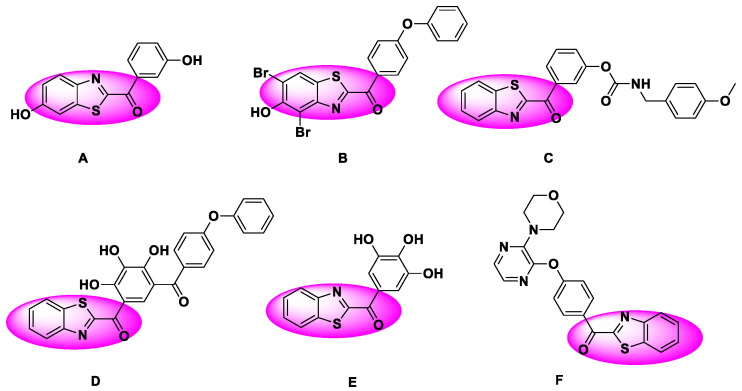
Several C2-arylacylated benzothiazole derivatives with potential as drug candidates.

Benzothiazole skeletons exist as key building blocks in natural products, drugs and agrochemicals and exhibit potent and diverse biological activities [1,2,3,4]. The benzothiazole scaffold is considered to be one of the most important substructures for drug discovery due to its various biological effects, including anti-cancer, anti-oxidant, anti-inflammatory, anti-microbial, anti-fungal, anti-convulsant, and anti-viral activities [5,6,7,8,9,10,11]. Among them, C2-arylacylated benzothiazole derivatives have attracted considerable attention in recent years due to their great potential as new drug candidates. For instance, as shown in Figure 1, 6-hydroxybenzothiophene ketone **A** exhibits potential for the treatment of breast cancer, endometriotic tissues, and other diseases by functioning as an inhibitor of 17b-hydroxysteroid dehydrogenase Type 1 (17b-HSD1) [12,13,14]. C2-arylacylated benzothiazole **B** act as a novel structural class of Ca^2+^/calmodulin-dependent protein kinase II (CaMKII) inhibitors with the potential to be developed as anti-inflammatory agents [15]; 2-Benzothiazolyl-phenylmethanone **C** were found to be potent fatty acid amide hydrolase (FAAH) inhibitors with beneficial effects for disorders such as pain and inflammation [16]. As a potent inhibitor of antiapoptotic Bcl-2 proteins, acylpyrogallol **D** inhibited growth and induced apoptosis in human breast and prostate cancer cell lines [17]. Keto-benzothiazole **E** acted as a potent antiproliferative agent for melanoma [18], while **F** can potentially treat schizophrenia by inhibiting PDE10A [19].

**Figure 2:**
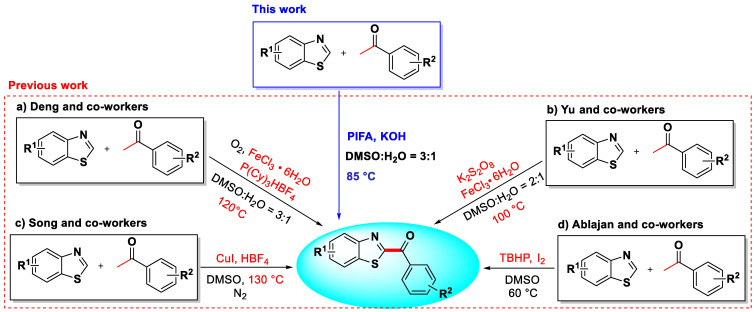
Representative arylacylation reactions of *2H*-benzothiazoles.

Given their extensive potential for therapeutic use, the development of efficient synthetic strategies for C2-arylacylated benzothiazoles is an attractive research topic. Up to now, several methods have been investigated for the synthesis of C2-arylacylated benzothiazoles, including cyclization with or without the sulfuration of *ortho*-substituted anilines [20,21,22,23] and sp^2^ C–H bond functionalization of *2H*-benzothiazoles. From the perspective of synthetic simplicity and atom economy, the direct C2-functionalization method is relatively advantageous. Great efforts have been devoted to preparing C2-arylacylated benzothiazoles by the direct C2-functionalization of *2H*-benzothiazoles with aryl methyl ketones [24,25,26,27], phenylglyoxal [28], *α*-oxocarboxylic acids [29,30], benzoic acid [31], and benzoyl chloride [32]. In particular, the direct C2-arylacylation of *2H*-benzothiazoles with aryl methyl ketones has generated great interest due to many “readily available” and “inexpensive” aryl methyl ketone analogs. In 2013, Deng and co-workers [24] reported the C2-arylacylation of *2H*-benzothiazoles using O_2_ as the oxidant in the presence of FeCl_3_·6H_2_O/P(Cy)_3_HBF_4_ at 120 °C (Figure 2a). In 2014, a FeCl_3_·6H_2_O-catalyzed C2-arylacylated reaction of *2H*-benzothiazoles using K_2_S_2_O_8_ as an oxidant was reported by Yu and co-workers [25] (Figure 2b). The same year, Song and co-workers [26] reported the CuI-catalyzed C2-arylacylation of *2H*-benzothiazoles under a nitrogen atmosphere (Figure 2c). In 2020, Ablajan and co-workers [27] developed a protocol for the C2-arylacylation of *2H*-benzothiazoles in the presence of I_2_ and TBHP (Figure 2d). Despite these notable advances, they still suffered from certain limitations such as relatively high reaction temperature, the transitional metal catalysts and inevitable metal residues, the use of the strongly corrosive additive HBF_4_, or the expensive ligand P(Cy)_3_HBF_4_. In addition, flammable and explosive organic peroxides were used as oxidizing reagents.

In recent years, PIFA has received significant attention as a mild, low-toxic, and selective reagent in organic synthesis, which can also be used as an effective alternative to toxic, heavy metal-based oxidants, and expensive organometallic catalysts [33,34,35]. In line with our continuous efforts to extend the C2-functionalization methods for *2H*-benzothiazoles [36,37,38,39,40], herein we report a novel, convenient, and efficient PIFA/KOH method which synergistically promotes the C2-arylacylation of *2H*-benzothiazoles with aryl methyl ketones, affording a wide variety of 2-arylacylated products with good yields. Our process also has the advantages of mild reaction conditions and being transitional metal-free.

## 2. Results and Discussion

### 2.1. Optimization of Reaction Conditions

Initially, we chose *2H*-benzothiazole (**1a**) and acetophenone (**2a**) as the model substrates to investigate the reaction conditions. The results are outlined in Table 1. Our examination of a series of common solvents demonstrated that the reactions led to the desired arylacylated product with a 7% yield in DMSO, while no product was produced in MeCN, DMF, and H_2_O (Table 1, entries 1–4). To our delight, the reaction efficiency was greatly improved when DMSO was mixed with H_2_O. The experimental results indicated that the product yield reached 60% in the mixture of DMSO and H_2_O with a volume ratio of 3:1 (Table 1, entries 5–8). In addition, the screening of different bases showed that KOH provided the greatest yield (75%) of the desired product (Table 1, entries 7 and 9–11). From the results in entries 11–14, it can be deduced that the base is necessary, with the optimal amount of KOH being one equivalent of **2a**. Furthermore, we changed the amount of PIFA, but poorer yields were obtained in all cases (Table 1, entries 16–18), and no product was observed in the absence of PIFA (Table 1, entry 15). Further investigation indicated that the temperature is very important for this transformation; the yield declined when the reaction temperature was decreased to 75 °C or increased to 95 °C (Table 1, entries 19–20). When the reaction time increased from 8 h to 10 h, the yield of **3aa** was increased to 86%, but no significant improvement was observed when the reaction time was increased to 12 h (Table 1, entries 21–22). Thus, the optimal reaction conditions involved PIFA (2 eq.) and KOH (1 eq.) in DMSO/H_2_O (3:1, *v*/*v*) at 85 °C for 10 h (Table 1, entry 21).

**Figure 3:**
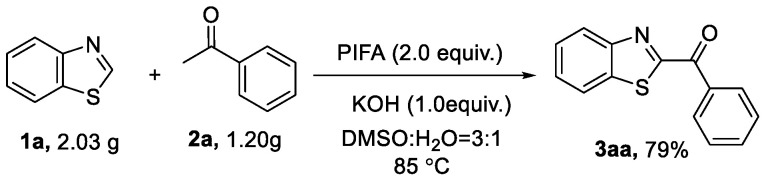
Gram-scale synthesis.

Notably, the procedure was applicable to a 10 mmol scale (1.20 g), and the product **3aa** was isolated in 79% (1.89 g) yield under the optimized reaction conditions (Figure 3).

### 2.2. Expansion of Substrate Scope

**Figure 4:**
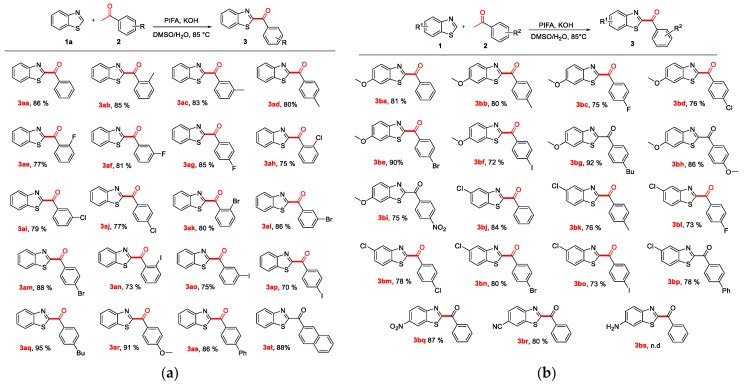
(**a**) Scope of substituted acetophenones. ^a a^ Reaction conditions: **1a** (1.5 eq., 0.45 mmol), **2** (0.30 mmol), PIFA (2.0 eq., 0.60 mmol), KOH (1.0 eq., 0.30 mmol), DMSO/H_2_O (*v*/*v*, 3/1, 2 mL), 85 °C, 10 h; (**b**) Scope of substituted benzothiazoles and substituted acetophenones. ^b b^ Reaction conditions: **1** (1.5 eq., 0.45 mmol), **2** (0.30 mmol), PIFA (2.0 eq., 0.60 mmol), KOH (1.0eq., 0.30 mmol), DMSO/H_2_O (*v*/*v*, 3/1, 2 mL), 85 °C, 10 h.

With these optimized reaction conditions in mind, the scope of aryl methyl ketones (**2**) was first explored for the arylacylation of *2H*-benzothiazole (**1a**). As shown in Figure 4, the reactions with aryl methyl ketones bearing a methyl group at *ortho*-, *meta*-, and *para*- positions gave the corresponding arylacylated products in 80–85% yields (Figure 4a, **3ab-3ad**). The halogenated acetophenones (2-F, 3-F, 4-F, 2-Cl, 3-Cl, 4-Cl, 2-Br,3-Br, 4-Br, 2-I, 3-I, and 4-I) produced corresponding products in 70–88% yield (Figure 4a, **3ae-3ap**). In addition, 4-butylacetophenone, 4-methoxyacetophenone, 4-phenylacetophenone, and 2-acetonaphthalene all formed corresponding arylacylated products in high yields of 86–95% (Figure 4a, **3aq-3at**). The above results demonstrated that a wide variety of aryl methyl ketones bearing either electron-donating or electron-withdrawing groups were well tolerated in this reaction.

Our scope was also extended to substituted *2H*-benzothiazoles and substituted aryl methyl ketones to investigate the utility and limits of the reaction (Figure 4b). To our delight, the reactions of 6-methoxybenzothiazole and 5-chlorobenzothiazole with aryl methyl ketones containing either electron-donating methyl and *n*-butyl groups or electron-withdrawing halogens; including fluoro, chloro, bromo, or iodo moieties or in addition to phenyl groups were well tolerated. These reactions all afforded corresponding arylacylated products in 72–92% yields (Figure 4b, **3ba-bp**). Meanwhile, 6-nitrobenzothiazole and 6-benzothiazolecarbonitrile were also tolerated by the reaction conditions, producing corresponding products **3bq** and **3br** in 87% and 80% yield, respectively. The results demonstrated that the *2H*-benzothiazoles bearing electron-withdrawing or electron-donating groups were compatible with a variety of aryl methyl ketones. Unfortunately, the desired reaction did not occur between 6-aminobenzothiazole and acetophenone (**3bs**), presumably because the amino group is readily oxidizable.

### 2.3. Mechanism Study

**Figure 5:**
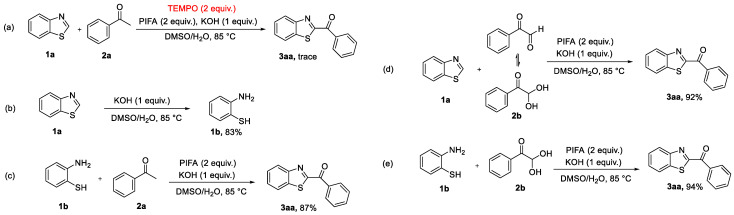
Mechanistic experiments.

In addition, a series of controlled experiments were used to explore the reaction mechanism as follows: (**A**) Two equivalents of the radical trap 2,2,6,6-tetramethyl-1-piperidinyloxy (TEMPO) were added to the mixture under optimal reaction conditions (Figure 5a). TEMPO, a known radical scavenger, will intercept the free radicals generated by the reaction, thus inhibiting the progress of the reaction [27]. The results indicated that the reaction was almost completely inhibited by TEMPO, with only a trace amount of **3aa** being detected. (**B**) The reaction mechanism behind the ring-opening of the *2H*-benzothiazole was explored. The *2H*-benzothiazole was transformed into 2-aminothiophenol (**1b**) in 83% yield under standard conditions (Figure 5b). (**C**) The reaction between 2-aminothiophenol (**1b**) and acetophenone (**2a**), under optimized reaction conditions, formed the target product (**3aa**) in 87% yield, which was close to that of the reaction between *2H*-benzothiazole and acetophenone (86%, Figure 4, **3aa**). This confirmed that the transformation might proceed via a ring-opening pathway. (**D**) The reaction between *2H-*benzothiazole (**1a**) and 2,2-dihydroxy-1-phenylethan-1-one (**2b**) afforded **3aa** in 92% yield (Figure 5d). This result demonstrated that acetophenone (**2a**) was oxidized to phenylglyoxal (**2b**). (**E**) The reaction of 2-aminothiophenol (**1b**) with 2,2-dihydroxy-1-phenylethan-1-one (**2b**) afforded the desired product **3aa** in 94% yield, suggesting that 2-aminothiophenol (**1b**) was the intermediate product (Figure 5e).

**Figure 6:**
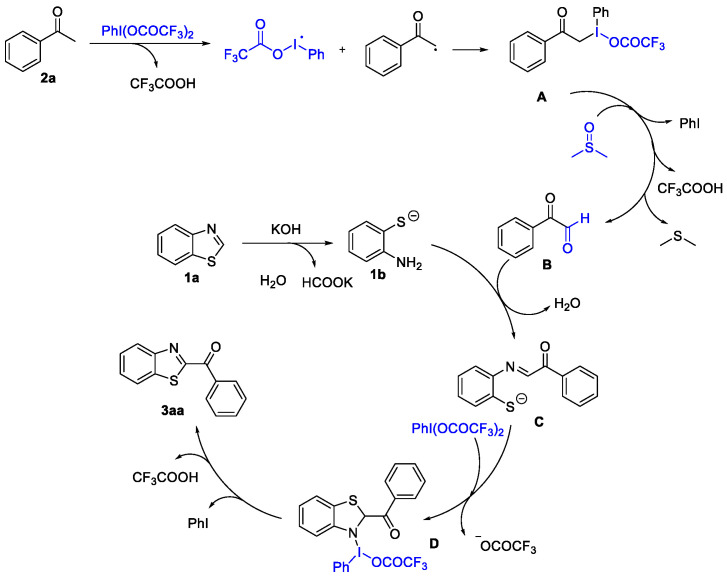
Plausible mechanism.

Based on the above experimental results and related reports [24,25,26,27], a plausible mechanism was proposed in Figure 6. Initially, acetophenone (**2a**) is converted to **A** in the presence of PIFA, which is similar to the I_2_ promoted sp^3^ C-H functionalization [41]. Subsequently, **A** was further oxidized to phenylglyoxal (**B**) in the DMSO [28]. In the meantime, the ringopening of *2H*-benzothiazole (**1a**) under KOH produced 2-aminothiophenol (**1b**) [42]. The condensation of **1b** with **B** formed an imine intermediate **C** [20,21,22,23], which could generate **D** through intramolecular cyclization [43]. Finally, the oxidative dehydrogenation of **D** furnished the target product **3aa** through the elimination of CF_3_COOH and PhI. The presence of the latter compound was detected in the reaction solution by GC-MS [44,45].

## 3. Conclusions

In summary, we have developed a novel PIFA/KOH synergistically promoted C2-arylacylation of *2H*-benzothiazoles using aryl methyl ketones as carbonyl sources. This arylacylation reaction tolerates a wide range of functional groups affording 38 examples of the C2-arylacylated products in 70–95% yield. This protocol provided an efficient and convenient method to synthesize C2-arylacylated benzothiazoles as potential drug scaffolds and complemented the existing approaches for the C2-arylacylation of aromatic rings.

## 4. Materials and Methods

### 4.1. General Information

All reactions were carried out under ambient atmosphere conditions in dried glassware. The reaction progress was monitored by TLC using silica gel GF254, and spots were visualized by exposure to UV light (254 nm). Melting points were determined using an X-4 apparatus without correction. NMR spectra were performed on a Bruker ADVANCE III instrument (500 MHz for ^1^H-NMR and 125 MHz for ^13^C-NMR) using TMS as an internal standard and CDCl_3_ or DMSO-*d*_6_ as the solvent. The high-resolution mass spectra (HRMS) were determined using a Shimadzu LCMS-IT-TOF mass spectrometer equipped with an electrospray ionization (ESI) source.

### 4.2. Synthesis

A mixture of *2H*-benzothiazole **1** (0.45 mmol), aryl methyl ketone **2** (0.30 mmol), KOH (1 equiv., 0.30 mmol), and PIFA (2 equiv., 0.60 mmol) was heated at 85 °C in DMSO/H_2_O (*v*/*v*, 3/1, 2 mL) for 10 h. After cooling to room temperature, the reaction mixture was diluted with H_2_O (30 mL) and extracted with dichloromethane (3 × 10 mL). The combined organic layers were then dried over anhydrous Na_2_SO_4_, filtered, and concentrated under vacuum. The resulting residue was purified by column chromatography (silica gel, petroleum ether/ethyl acetate, 20:1 to 8:1) to give products **3aa-3bs**.

*Benzo[d]thiazol-2-yl(phenyl)methanone* (**3aa**): Yellow solid, yield 86% (61 mg); m.p.: 98–99 °C; ^1^H NMR (500 MHz, CDCl_3_) *δ* 8.58 (dd, *J* = 8.0, 1.0 Hz, 2H), 8.27 (d, *J* = 7.5 Hz, 1H), 8.04 (d, *J* = 7.0 Hz, 1H), 7.73–7.65 (m, 1H), 7.64–7.54 (m, 4H); ^13^C NMR (125 MHz, CDCl_3_) *δ* 185.40, 167.17, 153.94, 137.06, 135.03, 133.91, 131.31, 128.53, 127.64, 126.95, 125.77, 122.19. These spectroscopic data correspond to the reported data in reference [27].

*Benzo[d]thiazol-2-yl(o-tolyl)methanone* (**3ab**): Yellow solid; yield 85% (64 mg); m.p.: 110–112 °C; ^1^H NMR (500 MHz, CDCl_3_) *δ* 8.21 (dd, *J* = 9.0, 1.5 Hz, 1H), 8.03 (td, *J* = 7.5, 1.0 Hz, 2H), 7.60–7.54 (m, 2H), 7.52–7.48 (m, 1H), 7.37 (t, *J* = 7.5 Hz, 2H), 2.55 (s, 3H); ^13^C NMR (125 MHz, CDCl_3_) *δ* 189.27, 167.61, 153.86, 139.11, 137.2, 135.25, 132.03, 131.58, 131.37, 127.69, 126.95, 125.86, 125.35, 122.26, 20.68. These spectroscopic data correspond to the reported data in reference [27].

*Benzo[d]thiazol-2-yl(m-tolyl)methanone* (**3ac**): Yellow solid; yield 83% (63 mg); m.p.: 72–74 °C; ^1^H NMR (500 MHz, CDCl_3_) *δ* 8.40 (d, *J* = 7.5 Hz, 1H), 8.30 (s, 1H), 8.29–8.26 (m, 1H), 8.05–8.03 (m, 1H), 7.63–7.57 (m, 2H), 7.51–7.45 (m, 2H), 2.50 (s, 3H); ^13^C NMR (125 MHz, CDCl_3_) 185.69, 167.29, 153.94, 138.35, 137.06, 135.07, 134.76, 131.50, 128.69, 128.43, 127.59, 126.91, 125.78, 122.19, 21.47. These spectroscopic data correspond to the reported data in reference [27].

*Benzo[d]thiazol-2-yl(p-tolyl)methanone* (**3ad**): Yellow solid; yield 80% (61 mg); m.p.: 96–98 °C; ^1^H NMR (500 MHz, CDCl_3_) *δ* 8.50 (d, *J* = 8.5 Hz, 1H), 8.31–8.14 (m, 1H), 8.11–7.92 (m, 1H), 7.58 (dtd, *J* = 21.0, 7.0, 1.5 Hz, 2H), 7.38 (d, *J* = 8.5 Hz, 1H), 2.49 (s, 2H); ^13^C NMR (125 MHz, CDCl_3_) *δ* 184.94, 153.94, 153.94, 137.01, 132.47, 131.44, 129.28, 127.49, 126.85, 125.69, 122.16, 21.85. These spectroscopic data correspond to the reported data in reference [27].

*Benzo[d]thiazol-2-yl(2-fluorophenyl)methanone* (**3ae**): Yellow solid; yield 77% (59 mg); m.p.: 81–83 °C; ^1^H NMR (500 MHz, CDCl_3_) *δ* 8.20 (d, *J* = 7.3 Hz, 1H), 8.06–8.01 (m, 2H), 7.64–7.55 (m, 3H), 7.34 (t, *J* = 7.9 Hz, 1H), 7.25 (dd, *J* = 17.7, 8.0 Hz, 1H); ^13^C NMR (125 MHz, CDCl_3_) *δ* 185.33, 166.21, 161.24 (d, *J* = 255.0 Hz), 153.72, 137.29, 134.41 (d, *J* = 8.8 Hz), 131.91, 127.86, 127.04, 125.86, 124.89 (d, *J* = 11.3 Hz), 124.00 (d, *J* = 3.75 Hz), 122.29, 116.69 (d, *J* = 21.0 Hz). These spectroscopic data correspond to the reported data in reference [26].

*Benzo[d]thiazol-2-yl(3-fluorophenyl)methanone* (**3af**): Yellow solid; yield 81% (62 mg); m.p.: 78–80 °C; ^1^H NMR (500 MHz, CDCl_3_) *δ* 8.41 (d, *J* = 8.0 Hz, 1H), 8.35 (ddd, *J* = 9.5, 2.5, 1.5 Hz, 1H), 8.28 (d, *J* = 7.0 Hz, 1H), 8.05 (d, *J* = 7.5 Hz, 1H), 7.64–7.55 (m, 3H), 7.39 (tdd, *J* = 8.0, 2.5, 1.0 Hz, 1H); ^13^C NMR (125 MHz, CDCl_3_) *δ* 183.97, 166.62, 153.90, 137.13, 130.16 (d, *J* = 7.5 Hz), 127.89, 127.11, 125.90, 122.23, 120.95 (d, *J* = 21.3 Hz), 118.17 (d, *J* = 23.8 Hz). These spectroscopic data correspond to the reported data in reference [26].

*Benzo[d]thiazol-2-yl(4-fluorophenyl)methanone* (**3ag**): Yellow solid; yield 85% (65.6 mg); m.p.: 100–102 °C; ^1^H NMR (500 MHz, CDCl_3_) *δ* 8.70 (dd, *J* = 8.5, 5.5 Hz, 2H), 8.29–8.24 (m, 1H), 8.06–8.02 (m, 1H), 7.60 (dtd, *J* = 15.5, 7.5, 1.5 Hz, 2H), 7.26 (t, *J* = 9.0 Hz, 2H); ^13^C NMR (125 MHz, CDCl_3_) *δ* 183.56, 167.06, 166.42 (d, *J* = 256.0 Hz), 153.87, 137.03, 134.19 (d, *J* = 8.8 Hz), 131.32 (d, *J* = 2.5 Hz), 127.70, 126.99, 125.71, 122.18, 115.73 (d, *J* = 22.5 Hz). These spectroscopic data correspond to the reported data in reference [26].

*Benzo[d]thiazol-2-yl(2-chlorophenyl)methanone* (**3ah**): Yellow solid; yield 75% (61 mg); m.p.: 92–93 °C; ^1^H NMR (500 MHz, CDCl_3_) *δ* 8.21–8.17 (m, 1H), 8.06–8.01 (m, 1H), 7.80–7.76 (m, 1H), 7.62–7.49 (m, 6H), 7.45 (ddd, *J* = 7.5, 6.0, 2.5 Hz, 1H); ^13^C NMR (125 MHz, CDCl_3_) *δ* 187.61, 165.95, 153.76, 137.45, 136.07, 132.63, 132.36, 130.79, 130.55, 127.97, 127.07, 126.49, 125.97, 122.33. These spectroscopic data correspond to the reported data in reference [26].

*Benzo[d]thiazol-2-yl(3-chlorophenyl)methanone* (**3ai**): Yellow solid; yield 79% (64 mg); m.p.: 128–129 °C; ^1^H NMR (500 MHz, CDCl_3_) *δ* 8.58 (t, *J* = 2.0 Hz, 1H), 8.50 (d, *J* = 8.0 Hz, 1H), 8.28 (d, *J* = 7.5 Hz, 1H), 8.05 (d, *J* = 7.5 Hz, 1H), 7.67–7.51 (m, 4H); ^13^C NMR (125 MHz, CDCl_3_) *δ* 184.05, 166.48, 153.86, 137.11, 136.50, 134.73, 133.80, 131.19, 129.82, 129.45, 127.90, 127.11, 125.91, 122.22. These spectroscopic data correspond to the reported data in reference [27].

*Benzo[d]thiazol-2-yl(4-chlorophenyl)methanone* (**3aj**): Yellow solid; yield 77% (60 mg); m.p.: 99–101 °C; ^1^H NMR (500 MHz, CDCl_3_) *δ* 8.58 (d, *J* = 8.5 Hz, 2H), 8.26 (d, *J* = 7.5 Hz, 1H), 8.05 (d, *J* = 7.5 Hz, 1H), 7.64–7.54 (m, 4H); ^13^C NMR (125 MHz, CDCl_3_) *δ* 184.06, 166.86, 153.89, 140.67, 137.10, 133.33, 132.76, 128.91, 127.82, 127.08, 125.80, 122.23. These spectroscopic data correspond to the reported data in reference [26].

*Benzo[d]thiazol-2-yl(2-bromophenyl)methanone* (**3ak**): Yellow solid; yield 80% (76 mg); m.p.: 103–105 °C; ^1^H NMR (500 MHz, CDCl_3_) *δ* 8.19 (d, *J* = 9.5 Hz, 1H), 8.04 (d, *J* = 9.5 Hz, 1H), 7.77–7.70 (m, 3H), 7.60–7.55 (m, 2H), 7.50 (td, *J* = 7.5, 1.0 Hz, 1H), 7.44 (td, *J* = 8.0, 2.0 Hz, 1H); ^13^C NMR (125 MHz, CDCl_3_) *δ* 188.31, 165.67, 153.76, 138.09, 137.50, 133.70, 132.37, 130.71, 128.00, 127.07, 125.99, 122.34, 120.64. These spectroscopic data correspond to the reported data in reference [26].

*Benzo[d]thiazol-2-yl(3-bromophenyl)methanone* (**3al**): Yellow solid; yield 86% (82 mg); m.p.: 98–100 °C; ^1^H NMR (500 MHz, CDCl_3_) *δ* 8.72 (t, *J* = 2.0 Hz, 1H), 8.55 (d, *J* = 8.0 Hz, 1H), 8.29 (d, *J* = 7.5 Hz, 1H), 8.05 (d, *J* = 7.5 Hz, 1H), 7.81 (d, *J* = 8.0 Hz, 1H), 7.64–7.57 (m, 2H), 7.47 (t, *J* = 8.0 Hz, 1H); ^13^C NMR (125 MHz, CDCl_3_) *δ* 183.99, 136.70, 134.05, 129.99, 127.91, 127.11, 125.93, 122.68, 122.22. These spectroscopic data correspond to the reported data in reference [27].

*Benzo[d]thiazol-2-yl(4-bromophenyl)methanone* (**3am**): Yellow solid; yield 88% (84 mg); m.p.: 92–94 °C; ^1^H NMR (500 MHz, CDCl_3_) *δ* 8.49 (d, *J* = 8.5 Hz, 2H), 8.25 (d, *J* = 7.5 Hz, 1H), 8.04 (d, *J* = 7.5 Hz, 1H), 7.72 (d, *J* = 8.5 Hz, 2H), 7.63–7.56 (m, 2H); ^13^C NMR (125 MHz, CDCl_3_) *δ* 184.27, 166.80, 153.88, 137.10, 133.74, 132.80, 131.89, 129.53, 127.83, 127.08, 125.81, 122.23. These spectroscopic data correspond to the reported data in reference [27].

*Benzo[d]thiazol-2-yl(2-iodophenyl)methanone* (**3an**): Yellow solid; yield 73% (79 mg); m.p.: 121–123 °C; ^1^H NMR (500 MHz, CDCl_3_) *δ* 8.18 (d, *J* = 8.0 Hz, 1H), 8.08–8.04 (m, 1H), 7.97 (d, *J* = 7.5 Hz, 1H), 7.74 (dd, *J* = 8.0, 2.0 Hz, 1H), 7.60–7.55 (m, 1H), 7.52–7.44 (m, 2H), 7.19 (td, *J* = 8.0, 1.5 Hz, 1H); ^13^C NMR (125 MHz, CDCl_3_) *δ* 167.98, 140.66, 136.18, 131.52, 131.30, 128.25, 126.40, 125.59, 123.77, 121.57, 96.35. ESI-HRMS calcd C_14_H_8_INOS [M + H]^+^ 365.9450; found, 365.9469.

*Benzo[d]thiazol-2-yl(3-iodophenyl)methanone* (**3ao**): Yellow solid; yield 75% (82 mg); m.p.: 105–107 °C; ^1^H NMR (500 MHz, CDCl_3_) *δ* 8.87 (t, *J* = 1.5 Hz, 1H), 8.59 (dt, *J* = 7.5, 1.5 Hz, 1H), 8.29–8.26 (m, 1H), 8.04–7.99 (m, 2H), 7.63–7.57 (m, 2H), 7.32 (t, *J* = 8.0 Hz, 1H); ^13^C NMR (125 MHz, CDCl_3_) *δ* 183.82, 166.36, 153.82, 142.53, 139.79, 137.08, 136.69, 130.50, 130.12, 127.87, 127.07, 125.90, 122.19, 94.02. ESI-HRMS calcd C_14_H_8_INOS [M + H]^+^ 365.9450; found, 365.9468.

*Benzo[d]thiazol-2-yl(4-iodophenyl)methanone* (**3ap**): Yellow solid; yield 70% (76 mg); m.p.: 113–115 °C; ^1^H NMR (500 MHz, CDCl_3_) *δ* 8.33–8.29 (m, 2H), 8.27–8.23 (m, 1H), 8.06–8.01 (m, 1H), 7.97–7.93 (m, 2H), 7.64–7.56 (m, 2H); ^13^C NMR (125 MHz, CDCl_3_) *δ* 184.58, 166.73, 153.83, 137.87, 137.07, 134.23, 132.56, 127.80, 127.05, 125.78, 122.20, 102.57. These spectroscopic data correspond to the reported data in reference [46].

*Benzo[d]thiazol-2-yl(4-butylphenyl)methanone* (**3aq**): Yellow liquid; yield 95% (84 mg); ^1^H NMR (500 MHz, CDCl_3_) *δ* 8.52 (d, *J* = 8.0 Hz, 2H), 8.31–8.19 (m, 1H), 8.07–7.91 (m, 1H), 7.62–7.52 (m, 2H), 7.38 (d, *J* = 8.4 Hz, 2H), 2.78–2.70 (m, 2H), 1.71–1.63 (m, 2H), 1.46–1.36 (m, 2H), 1.02–0.91 (m, 3H); ^13^C NMR (125 MHz, CDCl_3_) *δ* 184.88, 167.51, 153.91, 149.88, 136.98, 132.60, 131.45, 128.64, 127.51, 126.82, 125.66, 122.13, 35.87, 33.15, 22.35, 13.91. ESI-HRMS calcd C_18_H_8_NOS [M + H]^+^ 296.1109; found, 296.1109.

*Benzo[d]thiazol-2-yl(4-methoxyphenyl)methanone* (**3ar**): Yellow solid; yield 91% (73 mg); m.p.: 126–128 °C; ^1^H NMR (500 MHz, CDCl_3_) *δ* 8.69–8.63 (m, 2H), 8.28–8.22 (m, 1H), 7.95–7.82 (m, 1H), 7.65–7.51 (m, 2H), 7.04–6.98 (m, 2H), 3.90 (s, 3H); ^13^C NMR (125 MHz, CDCl_3_) *δ* 183.41, 167.91, 164.42, 153.91, 136.90, 133.85, 127.80, 127.35, 126.77, 125.53, 122.11, 113.89, 55.55. These spectroscopic data correspond to the reported data in reference [26].

*[1,1′-Biphenyl]-4-yl(benzo[d]thiazol-2-yl)methanone* (**3as**): White solid; yield 86% (81 mg); m.p.: 100–102 °C; ^1^H NMR (500 MHz, CDCl_3_) *δ* 8.68 (d, *J* = 8.5 Hz, 2H), 8.29 (d, *J* = 7.5 Hz, 1H), 8.05 (d, *J* = 7.0 Hz, 1H), 7.81 (d, *J* = 8.0 Hz, 2H), 7.70 (d, *J* = 7.0 Hz, 2H), 7.63–7.57 (m, 2H), 7.55–7.49 (m, 2H), 7.47–7.42 (m, 1H); ^13^C NMR (125 MHz, CDCl_3_) *δ* 184.78, 167.35, 153.93, 146.56, 139.90, 137.04, 133.71, 131.89, 128.99, 128.38, 127.60, 127.37, 127.17, 126.93, 125.73, 122.18. These spectroscopic data correspond to the reported data in reference [46].

*Benzo[d]thiazol-2-yl(naphthalen-2-yl)methanone* (**3at**): Yellow solid; yield 88% (76 mg); m.p.: 145–147 °C; ^1^H NMR (500 MHz, CDCl_3_) *δ* 9.36 (s, 1H), 8.46 (d, *J* = 9.0 Hz, 1H), 8.32 (d, *J* = 7.5 Hz, 1H), 8.10 (d, *J* = 8.5 Hz, 1H), 8.00 (d, *J* = 8.5 Hz, 1H), 7.70–7.58 (m, 4H); ^13^C NMR (125 MHz, CDCl_3_) *δ* 185.07, 167.41, 153.96, 137.04, 135.98, 134.36, 132.48, 132.24, 130.24, 129.02, 128.34, 127.78, 127.58, 126.91, 126.73, 125.83, 125.76, 122.18. These spectroscopic data correspond to the reported data in reference [26].

*(6-Methoxybenzo[d]thiazol-2-yl)(phenyl)methanone* (**3ba**): Yellow solid; yield 81% (65 mg); m.p.: 138–140 °C; ^1^H NMR (500 MHz, CDCl_3_) *δ* 8.56 (dd, *J* = 8.0, 1.0 Hz, 2H), 8.12 (d, *J* = 9.0 Hz, 1H), 7.67 (t, *J* = 7.5 Hz, 1H), 7.57 (t, *J* = 8.0 Hz, 2H), 7.43 (d, *J* = 2.5 Hz, 1H), 7.20 (dd, *J* = 9.0, 2.5 Hz, 1H), 3.95 (s, 3H); ^13^C NMR (125 MHz, CDCl_3_) *δ* 185.21, 164.70, 159.84, 148.61, 139.16, 135.26, 133.68, 131.20, 128.46, 126.51, 117.63, 103.49, 55.89. These spectroscopic data correspond to the reported data in reference [36].

*(6-Methoxybenzo[d]thiazol-2-yl)(p-tolyl)methanone* (**3bb**): Yellow solid; yield 80% (68 mg); m.p.: 153–155 °C; ^1^H NMR (500 MHz, CDCl_3_) *δ* 8.48 (d, *J* = 8.0 Hz, 2H), 8.11 (d, *J* = 9.0 Hz, 1H), 7.42 (d, *J* = 2.5 Hz, 1H), 7.37 (d, *J* = 8.0 Hz, 2H), 7.19 (dd, *J* = 9.5, 3.0 Hz, 1H), 3.94 (s, 3H), 2.48 (s, 3H); ^13^C NMR (125 MHz, CDCl_3_) *δ* 184.72, 164.98, 159.67, 148.54, 144.71, 139.03, 132.60, 131.29, 129.19, 126.39, 117.50, 103.40, 55.85, 21.83. These spectroscopic data correspond to the reported data in reference [36].

*(4-Fluorophenyl)(6-methoxybenzo[d]thiazol-2-yl)methanone* (3bc): Yellow solid; yield 75% (63 mg); m.p.: 167−169 °C; ^1^H NMR (500 MHz, CDCl_3_) *δ* 8.67 (dd, *J* = 8.5, 5.5 Hz, 2H), 8.11 (d, *J* = 9.5 Hz, 1H), 7.43 (d, *J* = 2.0 Hz, 1H), 7.27–7.20 (m, 3H), 3.94 (s, 3H); ^13^C NMR (125 MHz, CDCl_3_) *δ* 183.35, 167.27, 165.23, 164.50, 159.84, 148.48, 139.10, 134.02 (d, *J* = 8.8 Hz), 126.43, 117.71, 115.64 (d, *J* = 22.5 Hz), 103.41, 55.89. These spectroscopic data correspond to the reported data in reference [36].

*(4-Chlorophenyl)(6-methoxybenzo[d]thiazol-2-yl)methanone* (**3bd**): Yellow solid; yield 76% (69 mg); m.p.: 195−197 °C; ^1^H NMR (500 MHz, CDCl_3_) *δ* 88.58–8.53 (m, 2H), 8.12 (d, *J* = 9.0 Hz, 1H), 7.56–7.53 (m, 2H), 7.43 (d, *J* = 2.5 Hz, 1H), 7.21 (dd, *J* = 9.0, 2.5 Hz, 1H), 3.95 (s, 3H); ^13^C NMR (125 MHz, CDCl_3_) *δ* 181.31, 164.28, 159.91, 148.48, 140.82, 140.34, 133.29, 132.60, 128.79, 126.50, 117.79, 103.39, 55.88. These spectroscopic data correspond to the reported data in reference [36].

*(4-Bromophenyl)(6-methoxybenzo[d]thiazol-2-yl)methanone* (**3be**): Yellow solid; yield 90% (93 mg); m.p.:184–186 °C; ^1^H NMR (500 MHz, CDCl_3_) *δ* 8.09–8.04 (m, 2H), 7.97 (d, *J* = 9.0 Hz, 1H), 7.52–7.46 (m, 3H), 7.37 (d, *J* = 2.5 Hz, 1H), 7.11 (dd, *J* = 9.0, 2.5 Hz, 1H), 3.91 (s, 3H); ^13^C NMR (125 MHz, CDCl_3_) *δ* 181.47, 164.14, 159.94, 148.61, 139.22, 132.68, 131.79, 128.60, 126.52, 117.81, 103.39, 55.88. These spectroscopic data correspond to the reported data in reference [27].

*(4-Iodophenyl)(6-methoxybenzo[d]thiazol-2-yl)methanone* (**3bf**): Yellow solid; yield 72% (85 mg); m.p.: 152–153 °C; ^1^H NMR (500 MHz, CDCl_3_) *δ* 8.31–8.26 (m, 2H), 8.11 (d, *J* = 9.0 Hz, 1H), 7.96–7.93 (m, 2H), 7.43 (d, *J* = 2.0 Hz, 1H), 7.21 (dd, *J* = 9.0, 2.5 Hz, 1H), 3.95 (s, 3H); ^13^C NMR (125 MHz, CDCl_3_) *δ* 184.38, 163.07, 159.92, 148.62, 145.50, 144.12, 142.39, 137.79, 132.47, 126.52, 117.80, 103.39, 99.99, 55.88. ESI-HRMS calcd C_15_H_10_INO_2_S [M + H]^+^ 359.9555; found, 359.9576.

*(4-Butylphenyl)(6-methoxybenzo[d]thiazol-2-yl)methanone* (**3bg**): Yellow solid; yield 92% (90 mg); m.p.: 100–102 °C; ^1^H NMR (500 MHz, CDCl_3_) *δ* 8.49 (d, *J* = 8.0 Hz, 2H), 8.11 (d, *J* = 9.5 Hz, 1H), 7.42 (d, *J* = 2.5 Hz, 1H), 7.37 (d, *J* = 8.5 Hz, 2H), 7.21–7.17 (m, 1H), 3.93 (s, 3H), 2.74–2.71 (m, 2H), 1.69–1.65 (m, 2H), 1.43–1.38 (m, 2H), 0.98–0.94 (m, 3H); ^13^C NMR (125 MHz, CDCl_3_) *δ* 184.71, 165.02, 159.67, 149.60, 139.02, 132.77, 131.32, 129.07, 128.57, 126.37, 117.48, 103.42, 55.83, 35.85, 33.17, 22.34, 13.90. ESI-HRMS calcd C_19_H_20_NO_2_S [M + H]^+^ 326.1215; found, 326.1220.

*(6-Methoxybenzo[d]thiazol-2-yl)(4-methoxyphenyl)methanone* (**3bh**): Yellow solid; yield 86% (77 mg); m.p.: 147–149 °C; ^1^H NMR (500 MHz, CDCl_3_) *δ* 8.65 (d, *J* = 9.0 Hz, 2H), 8.11 (d, *J* = 9.0 Hz, 1H), 7.43 (d, *J* = 2.5 Hz, 1H), 7.19 (d, *J* = 9.0 Hz, 1H), 7.05 (d, *J* = 9.0 Hz, 2H), 3.94 (s, 6H).; ^13^C NMR (125 MHz, CDCl_3_) *δ* 183.26, 165.43, 164.23, 159.59, 148.55, 138.92, 133.70, 127.99, 126.25, 117.39, 113.82, 103.44, 55.84, 55.53. These spectroscopic data correspond to the reported data in reference [27].

*(6-Methoxybenzo[d]thiazol-2-yl)(4-nitrophenyl)methanone* (**3bi**): Yellow solid; yield 75% (71 mg); m.p.: 152–153 °C; ^1^H NMR (500 MHz, CDCl_3_) *δ* 8.73 (d, *J* = 9.0 Hz, 2H), 8.40 (d, *J* = 9.0 Hz, 2H), 8.13 (d, *J* = 9.5 Hz, 1H), 8.02 (d, *J* = 9.0 Hz, 1H), 7.44 (d, *J* = 2.5 Hz, 1H), 3.96 (s, 3H); ^13^C NMR (125 MHz, CDCl_3_) *δ* 183.58, 163.29, 160.30, 148.69, 139.35, 137.18, 137.05, 132.16, 127.81, 126.73, 123.42, 116.60, 103.40, 55.86. These spectroscopic data correspond to the reported data in reference [47].

*(5-Chlorobenzo[d]thiazol-2-yl)(phenyl)methanone* (**3bj**): Yellow solid; yield 84% (68 mg); m.p. = 133–135 °C; ^1^H NMR (500 MHz, CDCl_3_) *δ* 8.59–8.55 (m, 2H), 8.26 (d, *J* = 2.0 Hz, 1H), 7.95 (d, *J* = 8.5 Hz, 1H), 7.70 (t, *J* = 7.5 Hz, 1H), 7.60–7.53 (m, 3H); ^13^C NMR (125 MHz, CDCl_3_) *δ* 184.94, 168.98, 154.66, 135.26, 134.72, 134.14, 133.06, 131.33, 128.59, 128.26, 125.28, 122.98. These spectroscopic data correspond to the reported data in reference [27].

*(5-Chlorobenzo[d]thiazol-2-yl)(p-tolyl)methanone* (**3bk**): Yellow solid; yield 76% (65 mg); m.p.: 92–93 °C; ^1^H NMR (500 MHz, CDCl_3_) *δ* 8.48 (d, *J* = 8.5 Hz, 2H), 8.22 (d, *J* = 1.5 Hz, 1H), 7.92 (d, *J* = 8.5 Hz, 1H), 7.50 (dd, *J* = 8.5, 1.5 Hz, 1H), 7.36 (d, *J* = 8.0 Hz, 2H), 2.48 (s, 3H); ^13^C NMR (125 MHz, CDCl_3_) *δ* 184.40, 169.32, 154.64, 145.28, 135.19, 132.93, 132.14, 131.44, 129.31, 128.07, 125.18, 122.91, 21.84. These spectroscopic data correspond to the reported data in reference [27].

*(5-Chlorobenzo[d]thiazol-2-yl)(4-fluorophenyl)methanone* (**3bl**): Yellow solid; yield 73% (63 mg); m.p.: 142–144 °C;^1^H NMR (500 MHz, CDCl_3_) *δ* 8.75–8.62 (m, 2H), 8.25 (d, *J* = 2.0 Hz, 1H), 7.96 (d, *J* = 8.5 Hz, 1H), 7.55 (dd, *J* = 8.5, 2.0 Hz, 1H), 7.25 (d, *J* = 8.5 Hz, 2H); ^13^C NMR (125 MHz, CDCl_3_) *δ* 183.15, 168.89, 165.55, 154.60, 135.24, 134.25 (d, *J* = 8.8 Hz), 133.15, 128.35, 125.25, 122.99, 115.85 (d, *J* = 22.5 Hz). These spectroscopic data correspond to the reported data in reference [48].

*(5-Chlorobenzo[d]thiazol-2-yl)(4-chlorophenyl)methanone* (**3bm**): Yellow solid; yield 78% (72 mg); m.p.: 185–187 °C; ^1^H NMR (500 MHz, CDCl_3_) *δ* 8.60–8.53 (m, 3H), 8.25 (d, *J* = 2.0 Hz, 1H), 7.96 (d, *J* = 8.5 Hz, 1H), 7.57–7.54 (m, 3H); ^13^C NMR (125 MHz, CDCl_3_) *δ* 183.61, 168.66, 154.60, 140.95, 135.29, 133.21, 133.00, 132.76, 128.97, 128.44, 125.31, 123.01. These spectroscopic data correspond to the reported data in reference [26].

*(4-Bromophenyl)(5-chlorobenzo[d]thiazol-2-yl)methanone* (**3bn**): Yellow solid; yield 80% (84 mg); m.p.: 184–186 °C; ^1^H NMR (500 MHz, CDCl_3_) *δ* 8.51–8.44 (m, 1H), 8.25 (d, *J* = 2.0 Hz, 1H), 7.96 (d, *J* = 9.0 Hz, 0H), 7.74–7.71 (m, 1H), 7.55 (dd, *J* = 8.5, 2.0 Hz, 1H); ^13^C NMR (125 MHz, CDCl_3_) *δ* 183.83, 168.60, 154.59, 135.29, 133.41, 133.22, 132.79, 131.96, 129.83, 128.45, 125.31, 123.00. These spectroscopic data correspond to the reported data in reference [27].

*(5-Chlorobenzo[d]thiazol-2-yl)(4-iodophenyl)methanone* (**3bo**): Yellow solid; yield 73% (87 mg); m.p.: 182–184 °C; ^1^H NMR (500 MHz, CDCl_3_) *δ* 8.32–8.30 (m, 2H), 8.25 (d, *J* = 2.0 Hz, 1H), 7.95 (d, *J* = 2.0 Hz, 1H), 7.70 (d, *J* = 4.5 Hz, 2H), 7.55 (dd, *J* = 8.5, 2.0 Hz, 1H); ^13^C NMR (125 MHz, CDCl_3_) *δ* 183.45, 169.19, 154.55, 135.26, 133.91, 133.17, 132.71, 131.90, 130.93, 128.42, 125.28, 122.99. ESI-HRMS calcd C_14_H_8_ClINOS [M + H]^+^ 399.9060; found, 399.9080.

*[1,1′-Biphenyl]-4-yl(5-chlorobenzo[d]thiazol-2-yl)methanone* (**3bp**): Yellow solid; yield 78% (81 mg); m.p.: 159–161 °C; ^1^H NMR (500 MHz, CDCl_3_) *δ* 8.68–8.63 (m, 2H), 8.27 (d, *J* = 1.5 Hz, 1H), 7.95 (d, *J* = 8.5 Hz, 1H), 7.81–7.78 (m, 2H), 7.72–7.68 (m, 2H), 7.55–7.50 (m, 3H), 7.47–7.42 (m, 1H); ^13^C NMR (125 MHz, CDCl_3_) *δ* 184.26, 169.17, 154.64, 146.77, 139.77, 135.23, 133.35, 133.02, 131.91, 129.01, 128.46, 128.19, 127.36, 127.19, 125.23, 122.95. ESI-HRMS calcd C_20_H_13_ClNOS [M + H]^+^ 350.0406; found, 350.0410.

*(6-Nitrobenzo[d]thiazol-2-yl)(phenyl)methanone* (**3bq**): Yellow solid; yield 87% (74 mg); m.p.: 159–161 °C; ^1^H NMR (500 MHz, CDCl_3_) *δ* 8.98 (d, *J* = 2.0 Hz, 1H), 8.61 (dd, *J* = 8.0, 0.5 Hz, 2H), 8.47 (dd, *J* = 9.0, 2.5 Hz, 1H), 8.39 (d, *J* = 9.0 Hz, 1H), 7.74 (t, *J* = 7.5 Hz, 1H), 7.61 (t, *J* = 8.0 Hz, 2H); ^13^C NMR (125 MHz, CDCl_3_) *δ* 191.14, 172.44, 157.19, 146.61, 137.14, 134.59, 134.26, 131.41, 128.75, 126.24, 122.08, 118.86. These spectroscopic data correspond to the reported data in reference [36].

*2-Benzoylbenzo[d]thiazole-6-carbonitrile* (**3br**): Yellow solid; yield 80% (63 mg); m.p.: 140–142 °C; ^1^H NMR (500 MHz, CDCl_3_) *δ* 8.59 (d, *J* = 7.0 Hz, 2H), 8.40 (d, *J* = 1.0 Hz, 1H), 8.35 (d, *J* = 8.5 Hz, 1H), 7.84 (dd, *J* = 8.5, 1.5 Hz, 1H), 7.73 (t, *J* = 7.5 Hz, 1H), 7.60 (t, *J* = 8.0 Hz, 2H); ^13^C NMR (125 MHz, CDCl_3_) *δ* 171.14, 155.94, 134.51, 134.34, 131.38, 129.67, 128.72, 127.26, 126.54, 118.30, 111.20. These spectroscopic data correspond to the reported data in reference [36].

## Figures and Tables

**Table 1:** Optimization of reaction conditions ^a^.

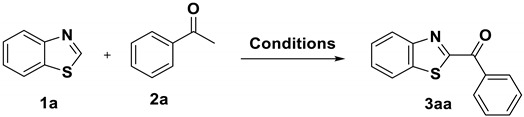				
**Entry**	**Oxidant (eq.)**	**Base (eq.)**	**Solvent (mL)**	**Yield (%) ^b^**
1	PIFA (2)	NaOH (1)	DMSO (2)	7
2	PIFA (2)	NaOH (1)	MeCN (2)	N.D.
3	PIFA (2)	NaOH (1)	DMF (2)	N.D.
4	PIFA (2)	NaOH (1)	H_2_O (2)	N.D.
5	PIFA (2)	NaOH (1)	DMSO/H_2_O 1:1 = (2)	36
6	PIFA (2)	NaOH (1)	DMSO/H_2_O 2:1 = (2)	48
7	PIFA (2)	NaOH (1)	DMSO/H_2_O 3:1 = (2)	60
8	PIFA (2)	NaOH (1)	DMSO/H_2_O 4:1 = (2)	53
9	PIFA (2)	K_2_CO_3_ (1)	DMSO/H_2_O 3:1 = (2)	35
10	PIFA (2)	Na_2_CO_3_ (1)	DMSO/H_2_O 3:1 = (2)	32
11	PIFA (2)	KOH (1)	DMSO/H_2_O 3:1 = (2)	75
12	PIFA (2)	none	DMSO/H_2_O 3:1 = (2)	N.D.
13	PIFA (2)	KOH (0.5)	DMSO/H_2_O 3:1 = (2)	48
14	PIFA (2)	KOH (1.5)	DMSO/H_2_O 3:1 = (2)	53
15	none	KOH (1)	DMSO/H_2_O 3:1 = (2)	N.D.
16	PIFA (0.5)	KOH (1)	DMSO/H_2_O 3:1 = (2)	26
17	PIFA (1.5)	KOH (1)	DMSO/H_2_O 3:1 = (2)	38
18	PIFA (2.5)	KOH (1)	DMSO/H_2_O 3:1 = (2)	51
19 ^c^	PIFA (2)	KOH (1)	DMSO/H_2_O 3:1 = (2)	58
20 ^d^	PIFA (2)	KOH (1)	DMSO/H_2_O 3:1 = (2)	65
21 ^e^	PIFA (2)	KOH (1)	DMSO/H_2_O 3:1 = (2)	86
22 ^f^	PIFA (2)	KOH (1)	DMSO/H_2_O 3:1 = (2)	85

^a^ Reaction conditions: **1a** (1.5 eq., 0.45 mmol), **2a** (0.30 mmol), oxidant, base in solvent at 85 °C for 8 h. ^b^ isolated yield. ^c^ 75 °C. ^d^ 95 °C. ^e^ 10 h. ^f^ 12 h.

## Data Availability

The data presented in this study is available in the article or supporting information.

## Supplementary Materials

The following are available online.

## Sample Availability

Samples of the compounds are available from the authors.
