# Supplementary material for: A Novel PIFA/KOH Promoted Approach to Synthesize C2-arylacylated Benzothiazoles as Potential Drug Scaffolds
Source: Molecules. 2022 Jan 22;27(3):726. doi: 10.3390/molecules27030726 (PMC8838045; doi:10.3390/molecules27030726)

Supporting Information

A novel PIFA/KOH promoted approach to synthesize C2-arylacylated benzothiazoles as potential drug scaffolds

Xiao-Tong Sun, Zhi-Gang Hu, Zhen Huang, Ling-Li Zhou and Jian-Quan Weng *

College of Chemical Engineering, Zhejiang University of Technology, Hangzhou 310014, P. R. China

**E-mail: jqweng@zjut.edu.cn.*

Table of Contents

1. NMR Spectrum of compounds 3aa~3br…………………………………………………………………… 2

2. NMR Spectrum of 1b…………………………………………………………………………………………… 40

3. GC-MS Spectrum of PhI………………………………………………………………………………………… 41

1. **NMR Spectrum of compounds 3aa~3br**

^{1}H and ^{13}C NMR Spectra of 3aa

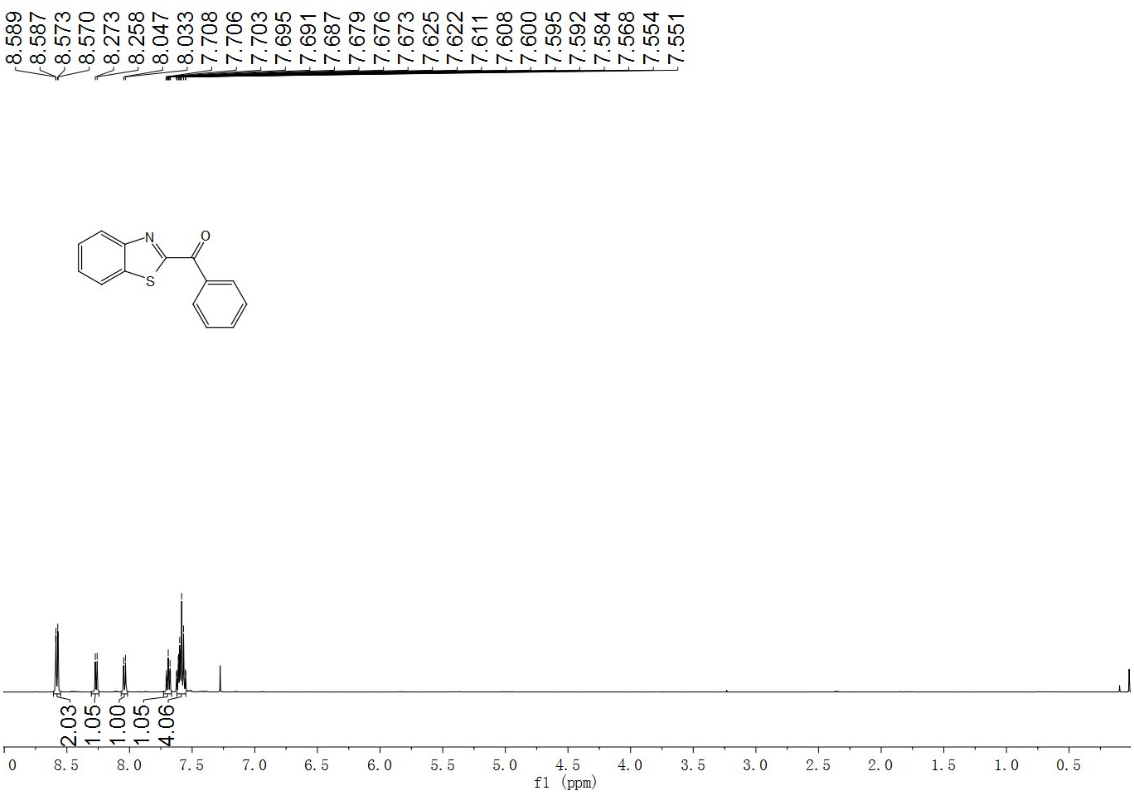

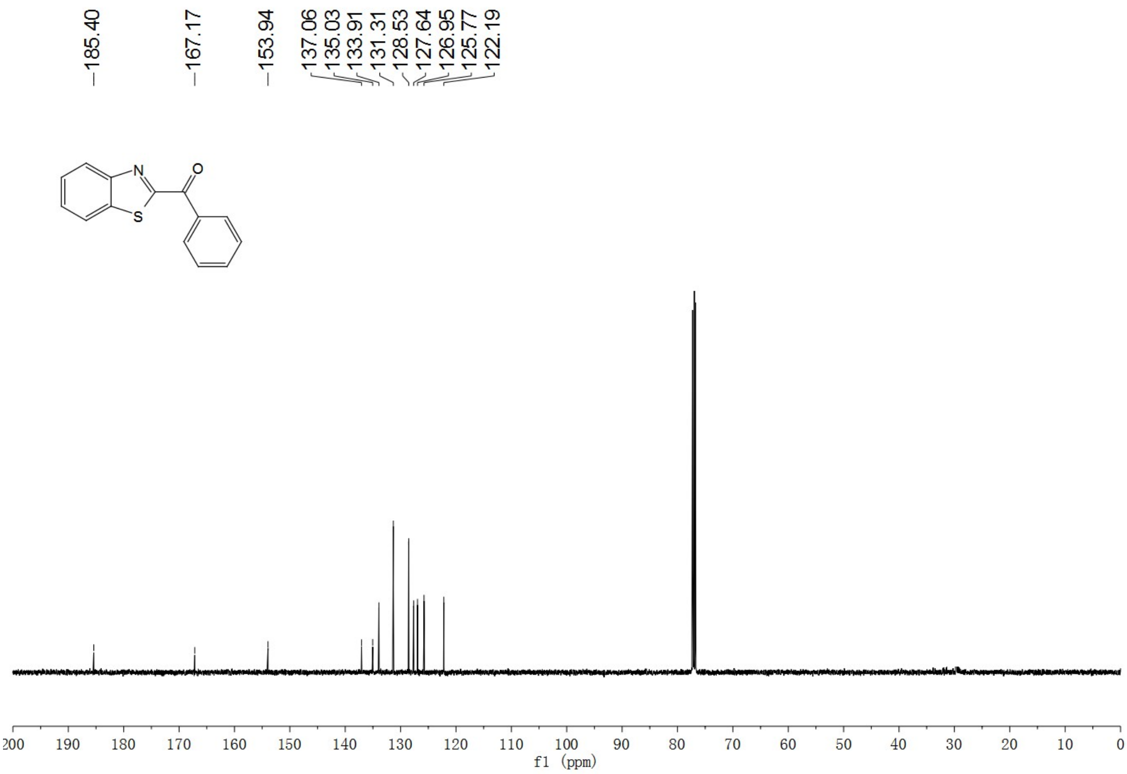

^{1}H and ^{13}C NMR Spectra of 3ab

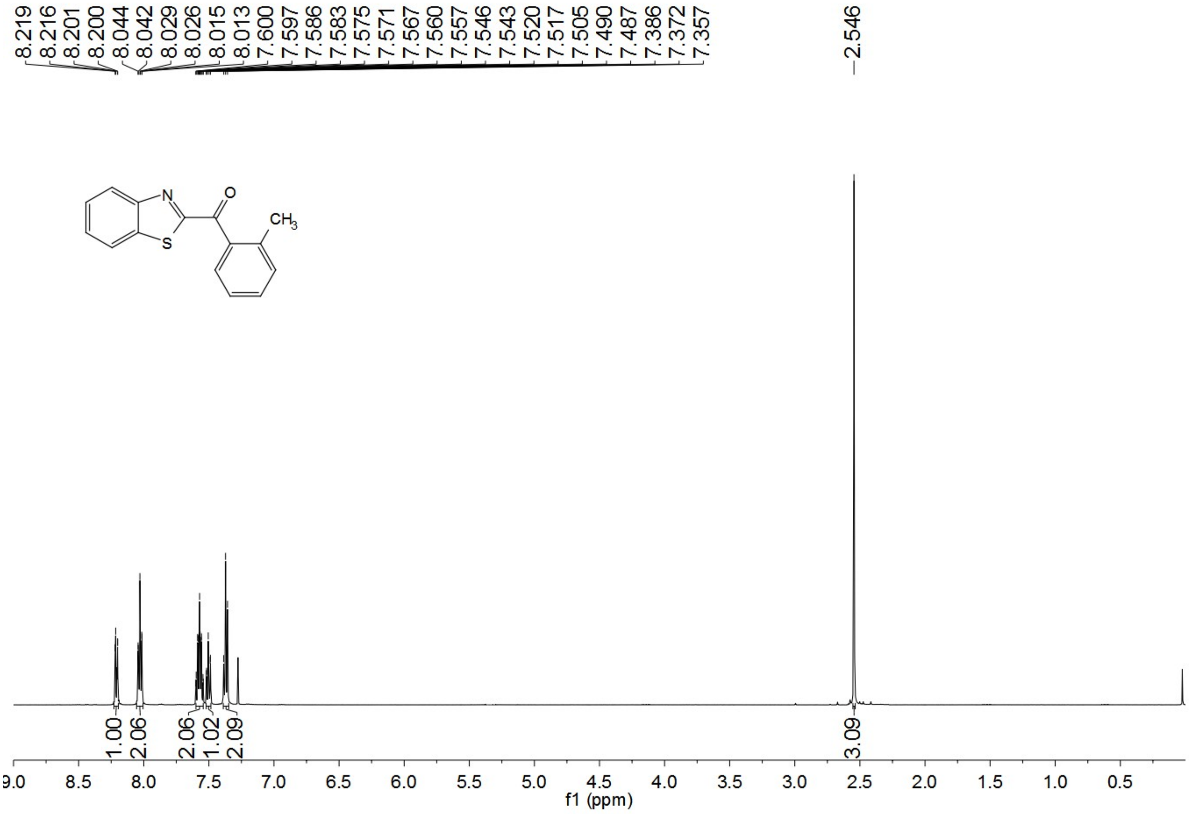

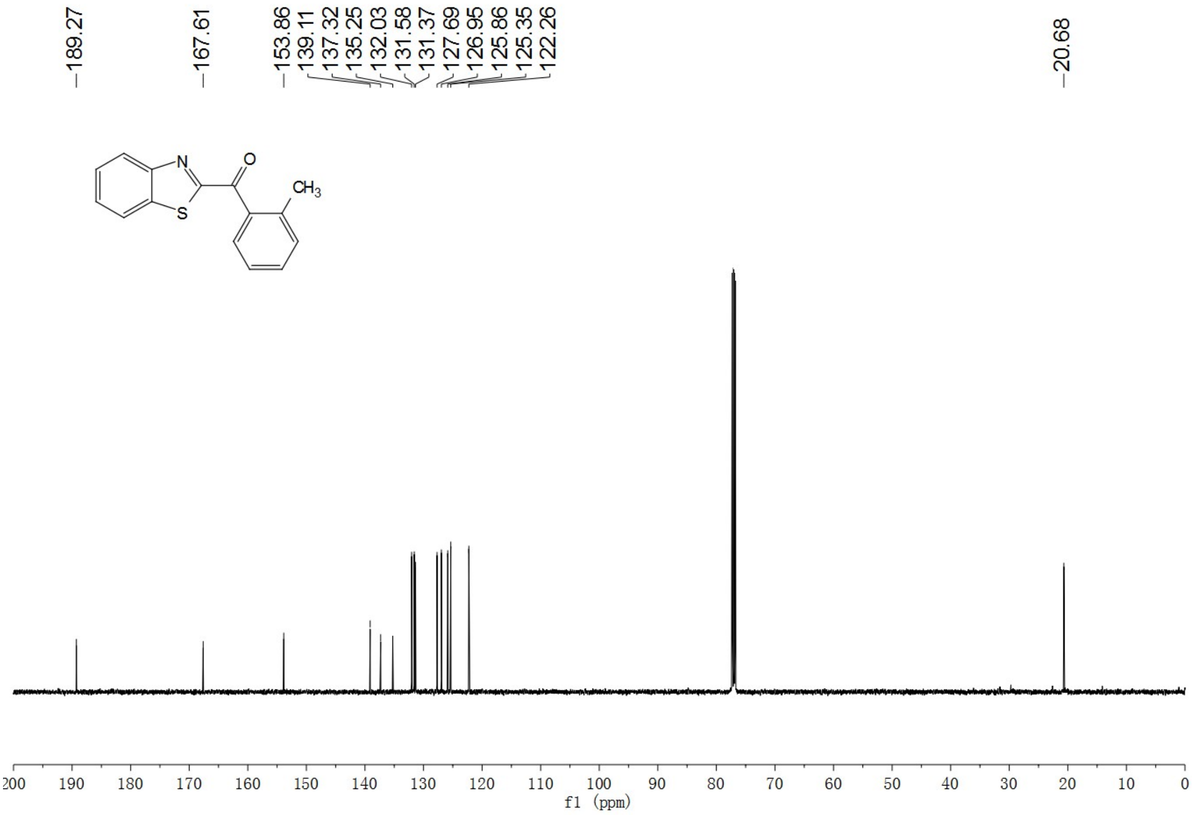

^{1}H and ^{13}C NMR Spectra of 3ac

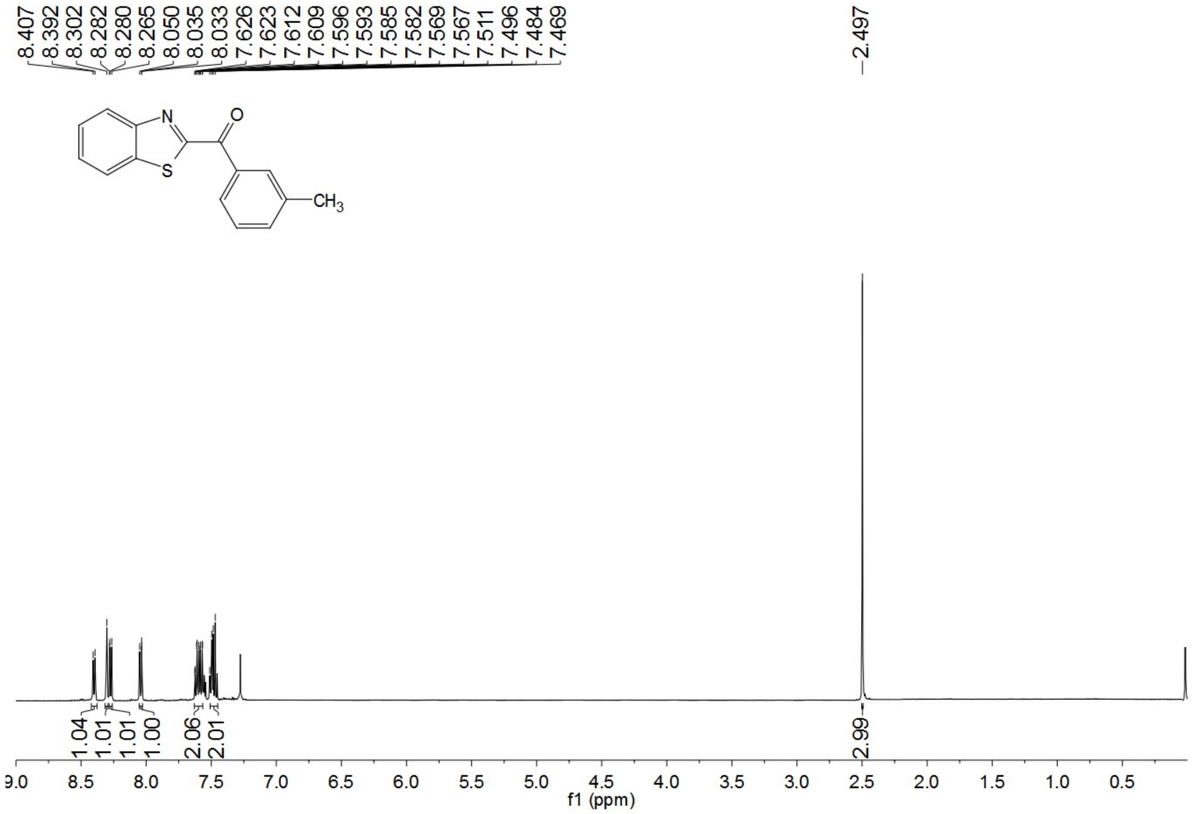

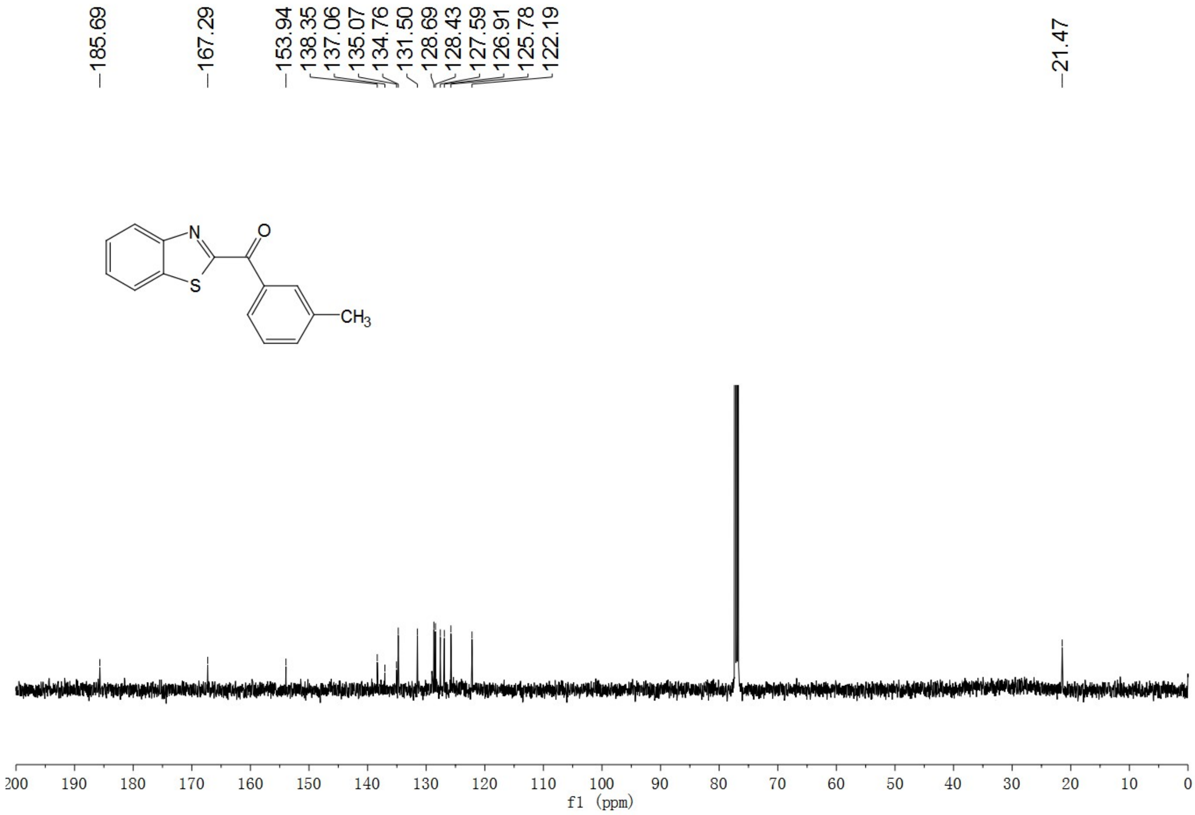

^{1}H and ^{13}C NMR Spectra of 3ad

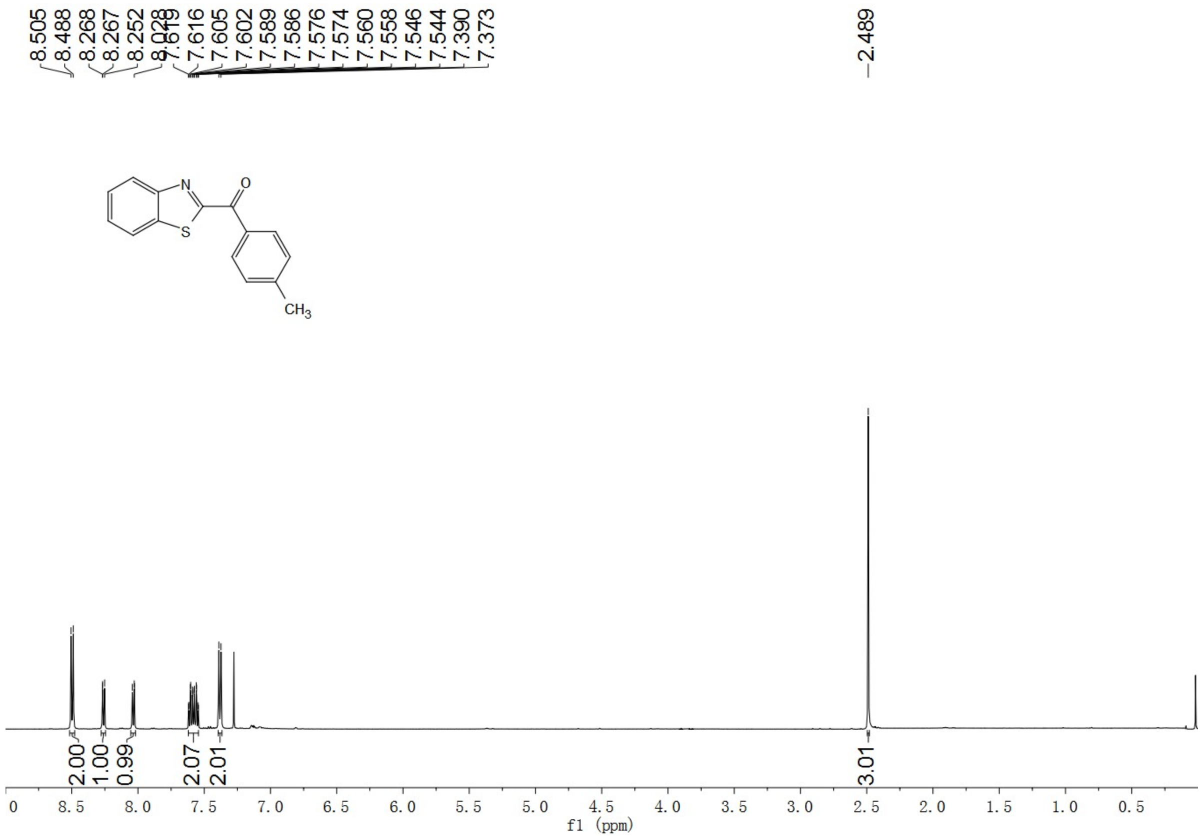

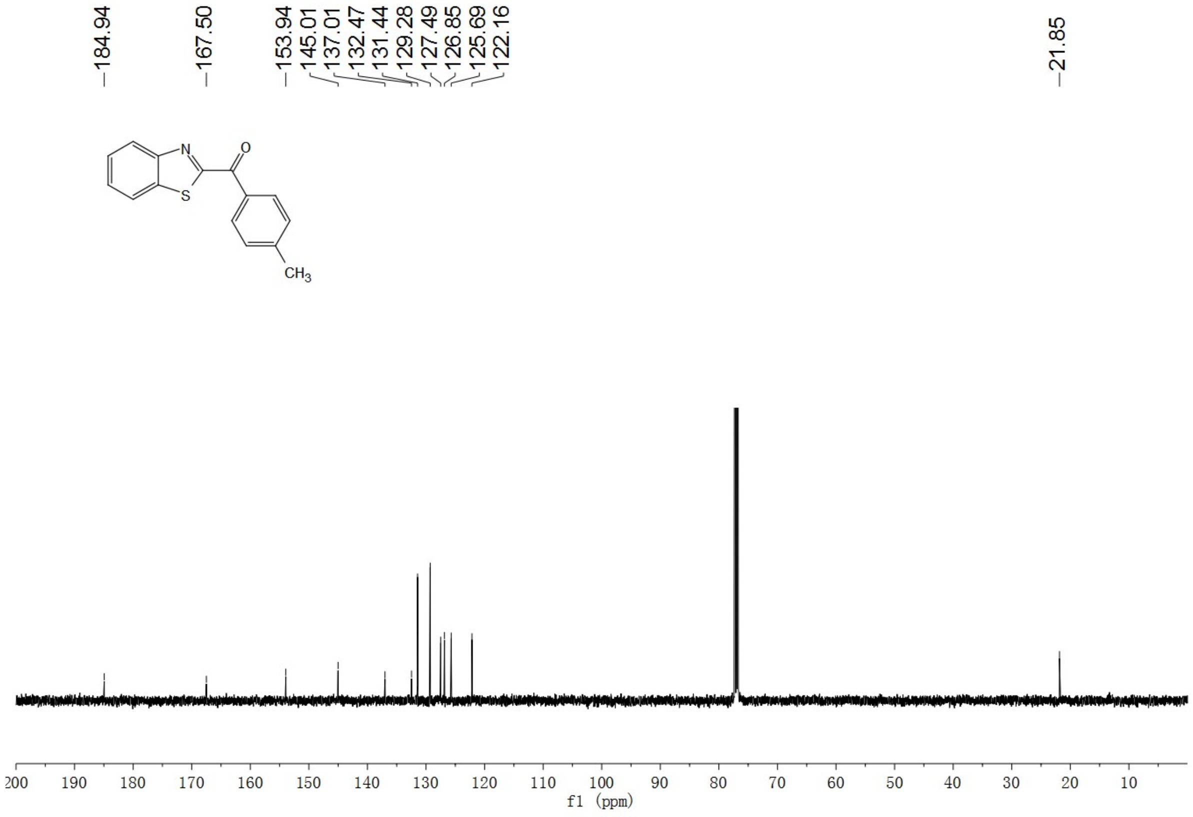

^{1}H and ^{13}C NMR Spectra of 3ae

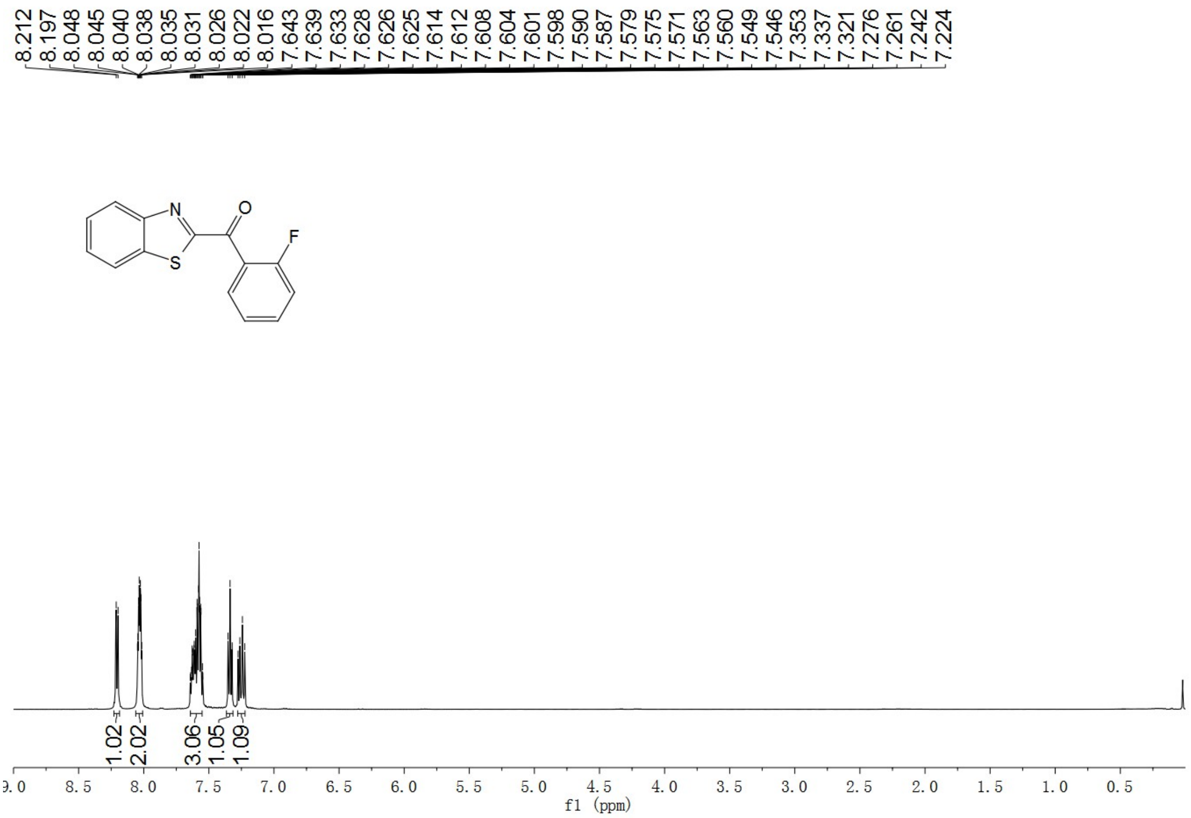

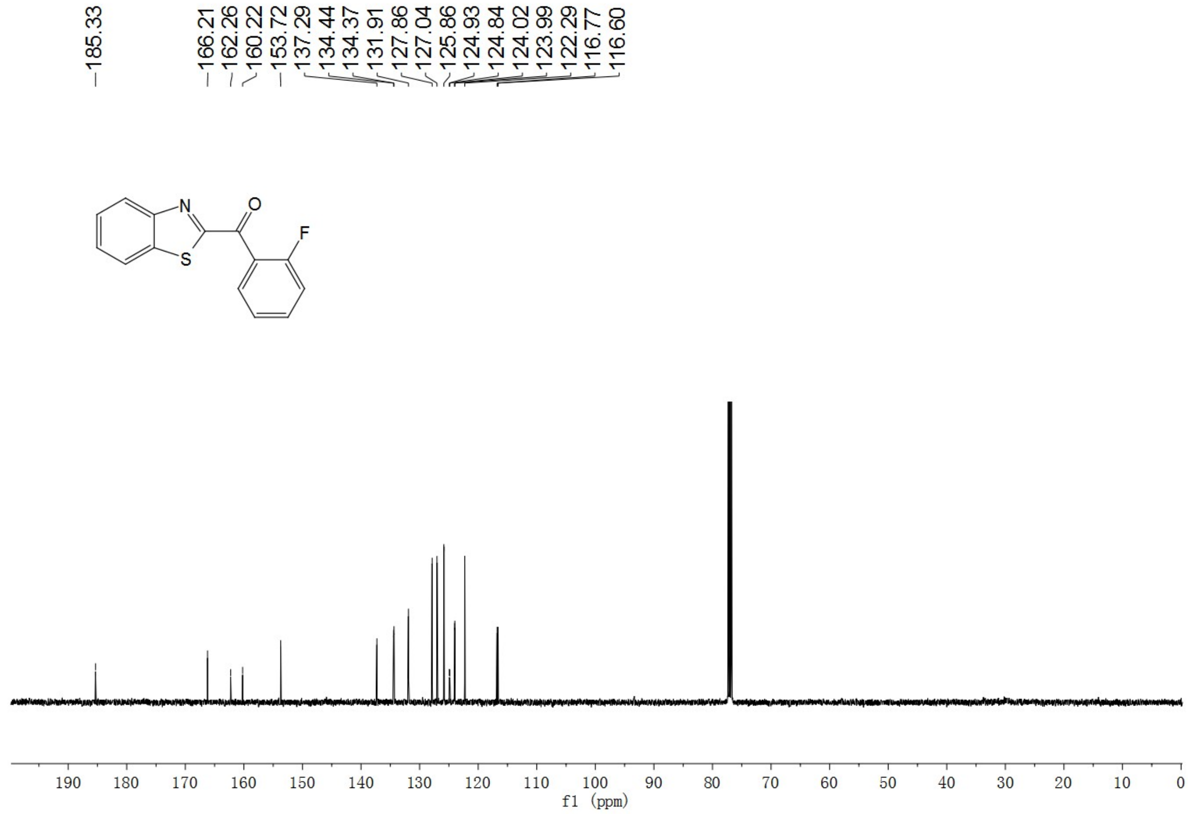

^{1}H and ^{13}C NMR Spectra of 3af

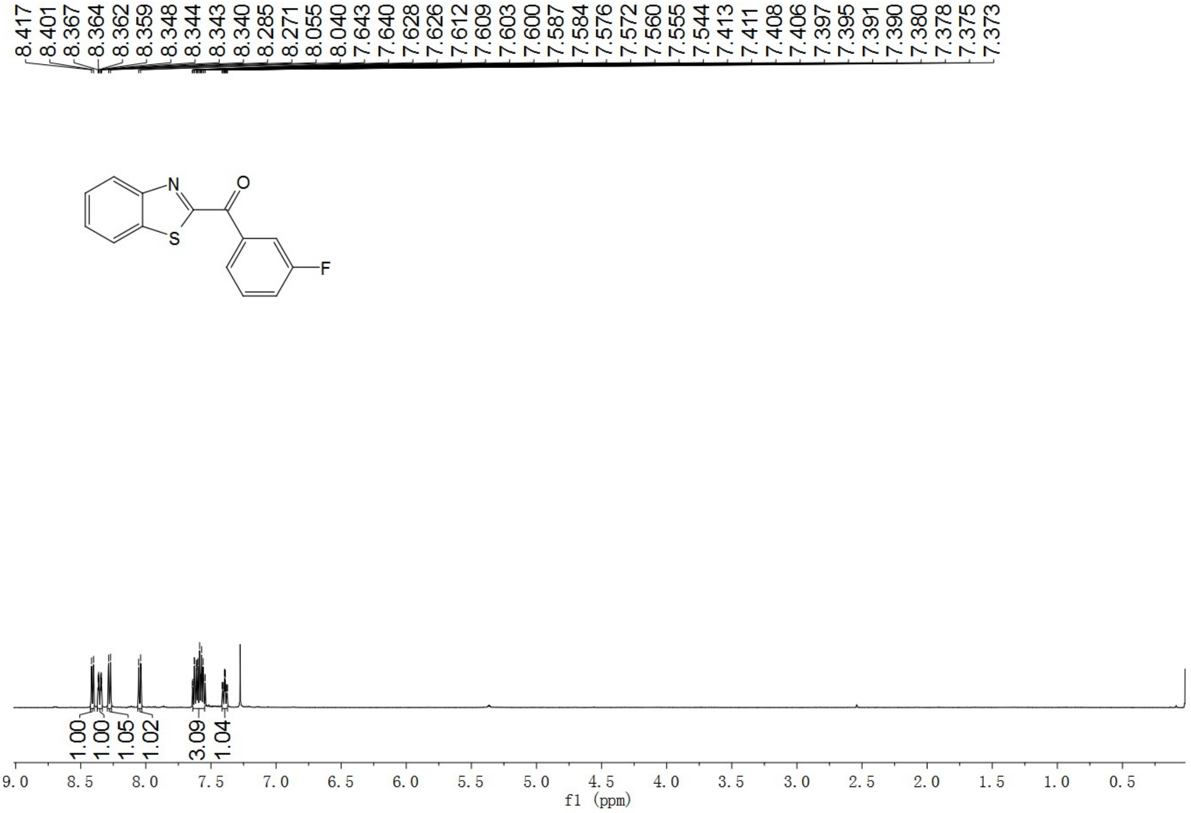

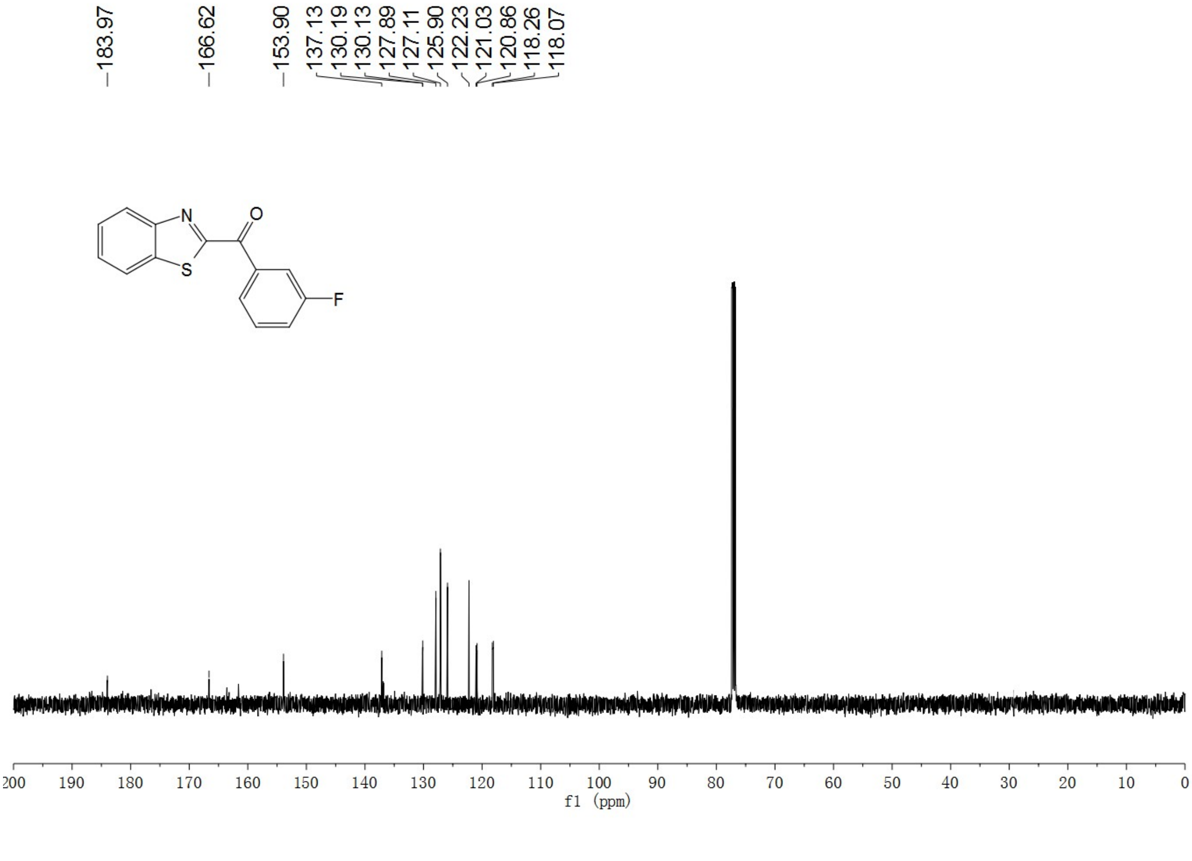

^{1}H and ^{13}C NMR Spectra of 3ag

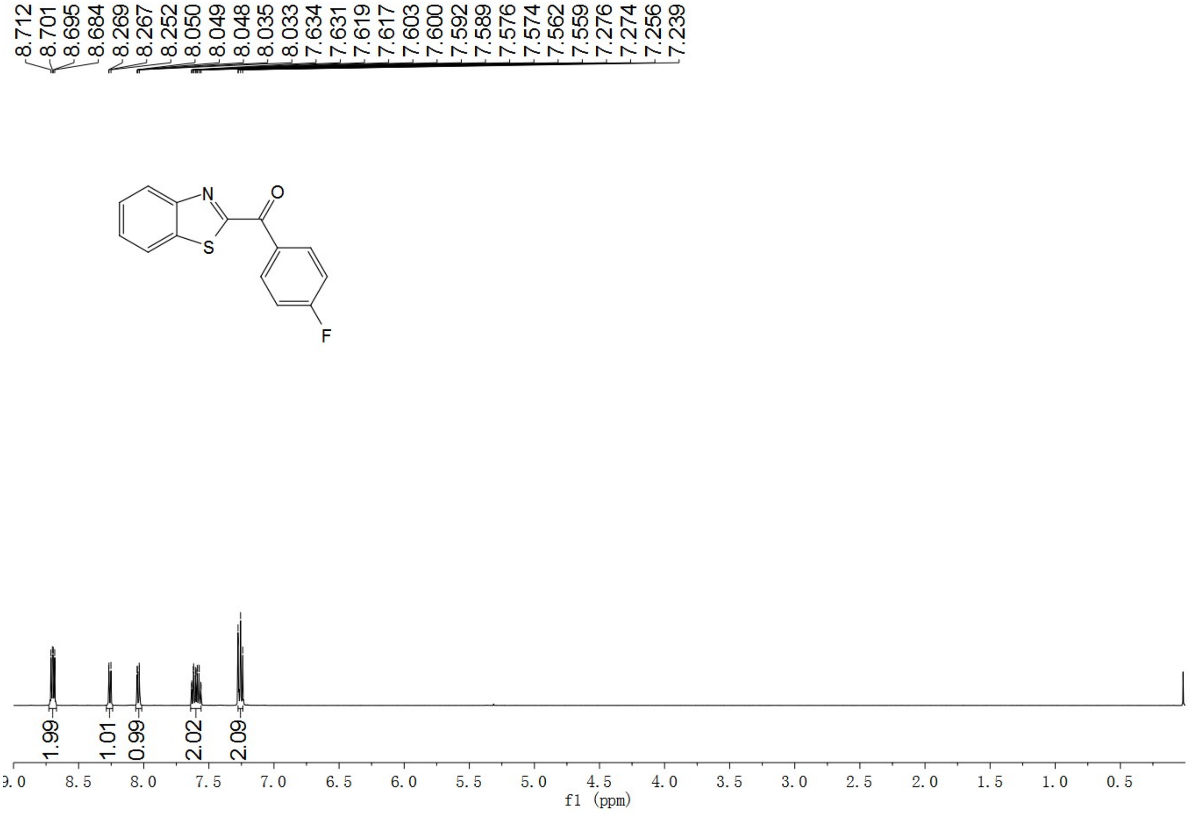

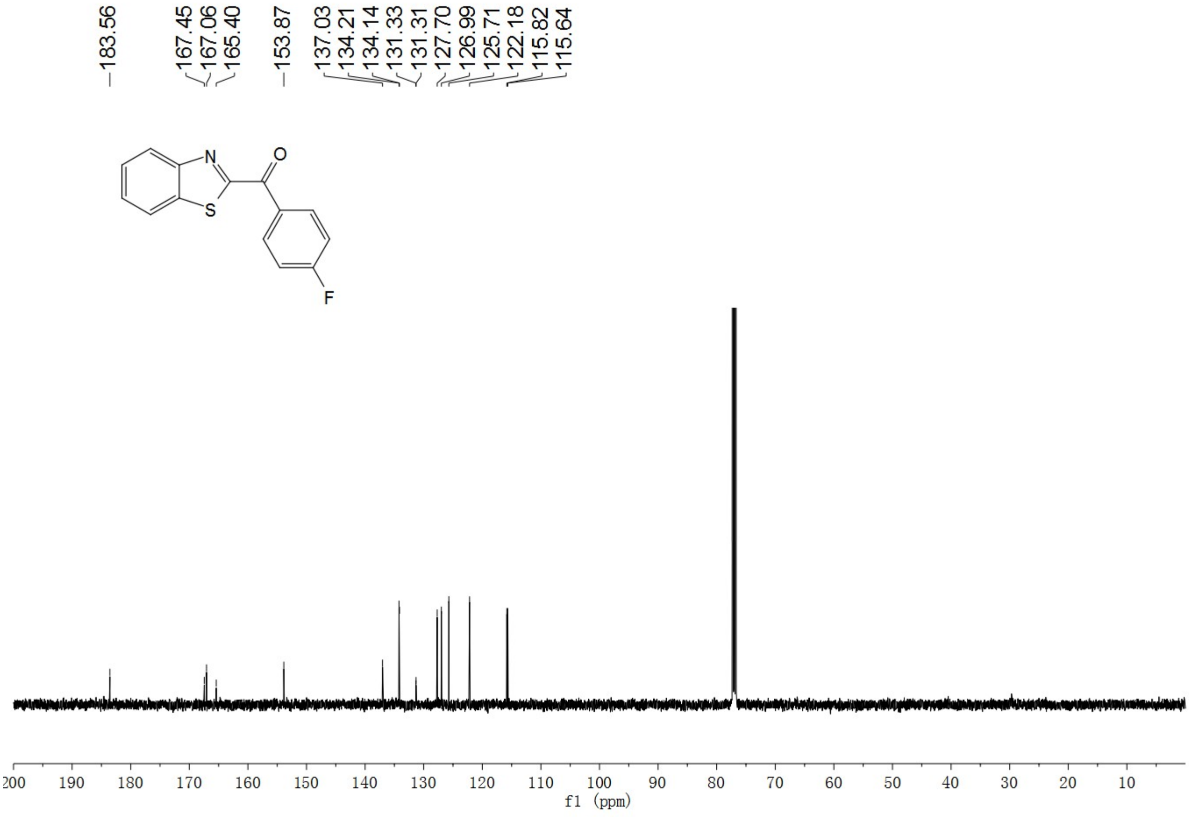

^{1}H and ^{13}C NMR Spectra of 3ah

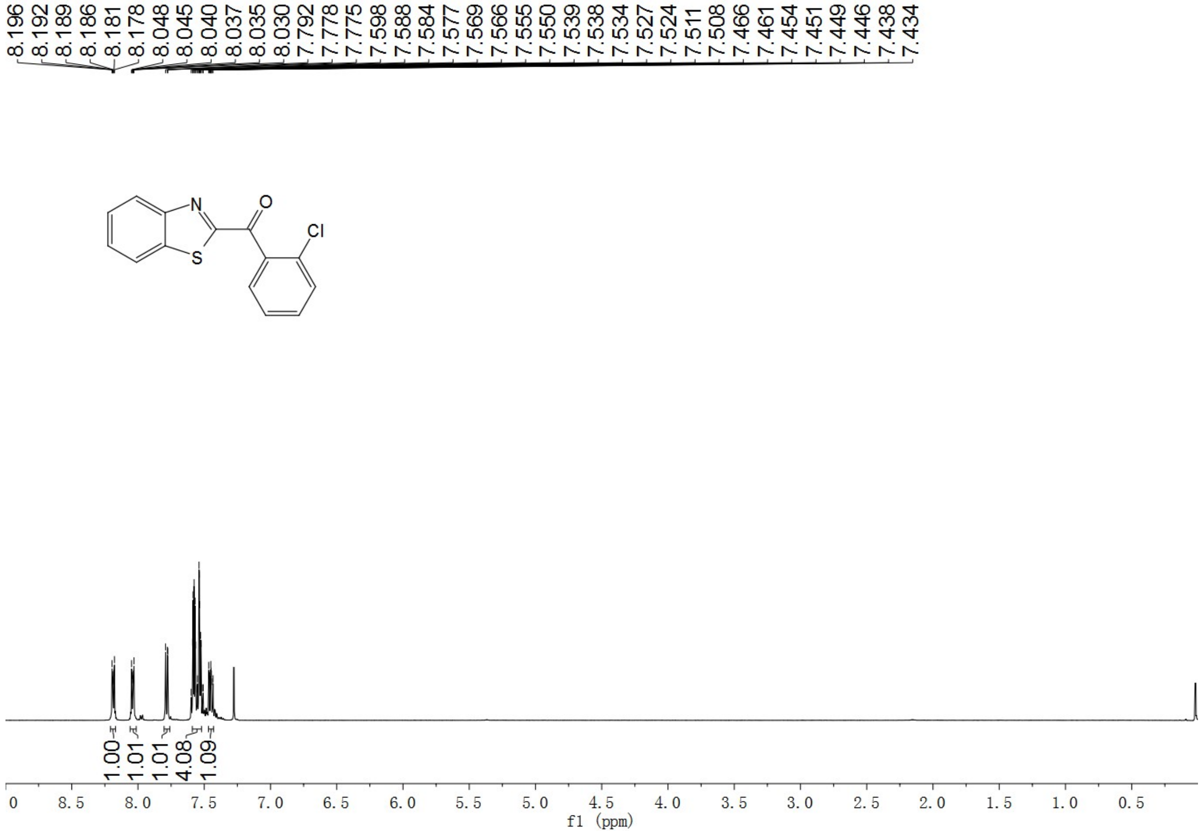

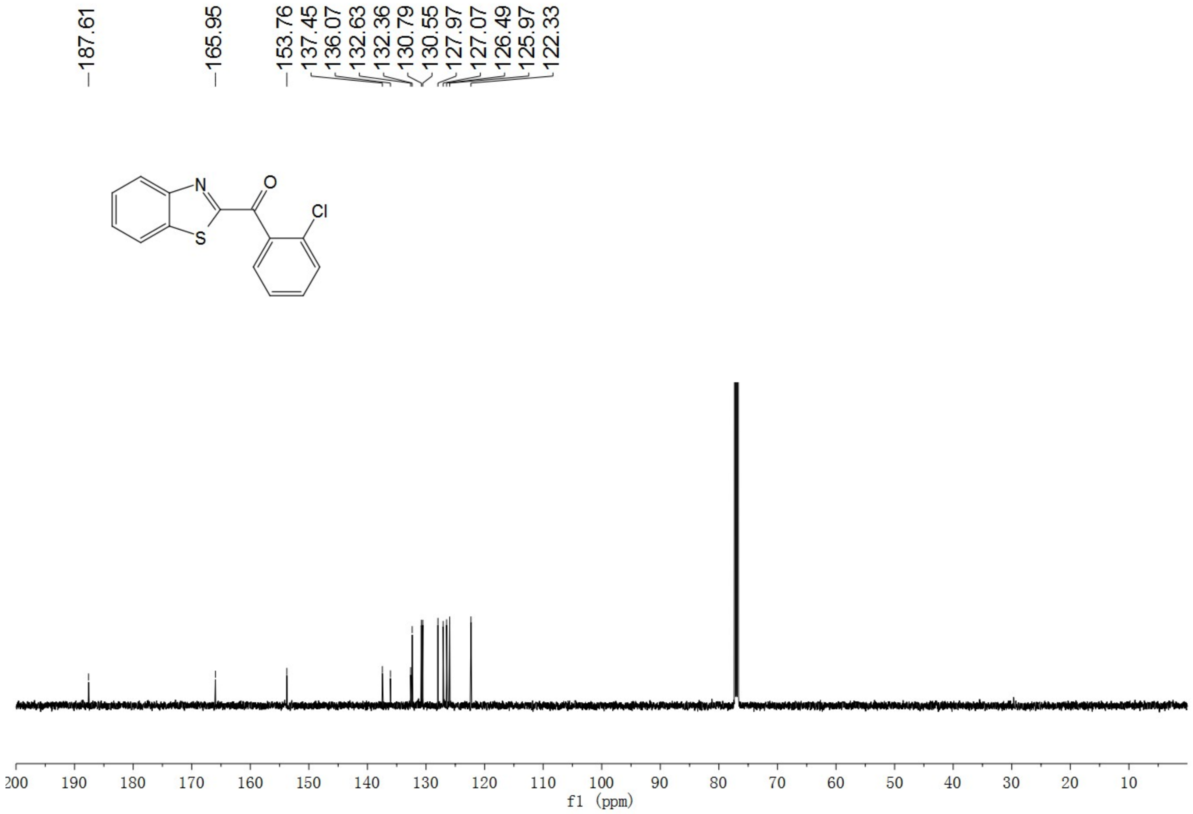

^{1}H and ^{13}C NMR Spectra of 3ai

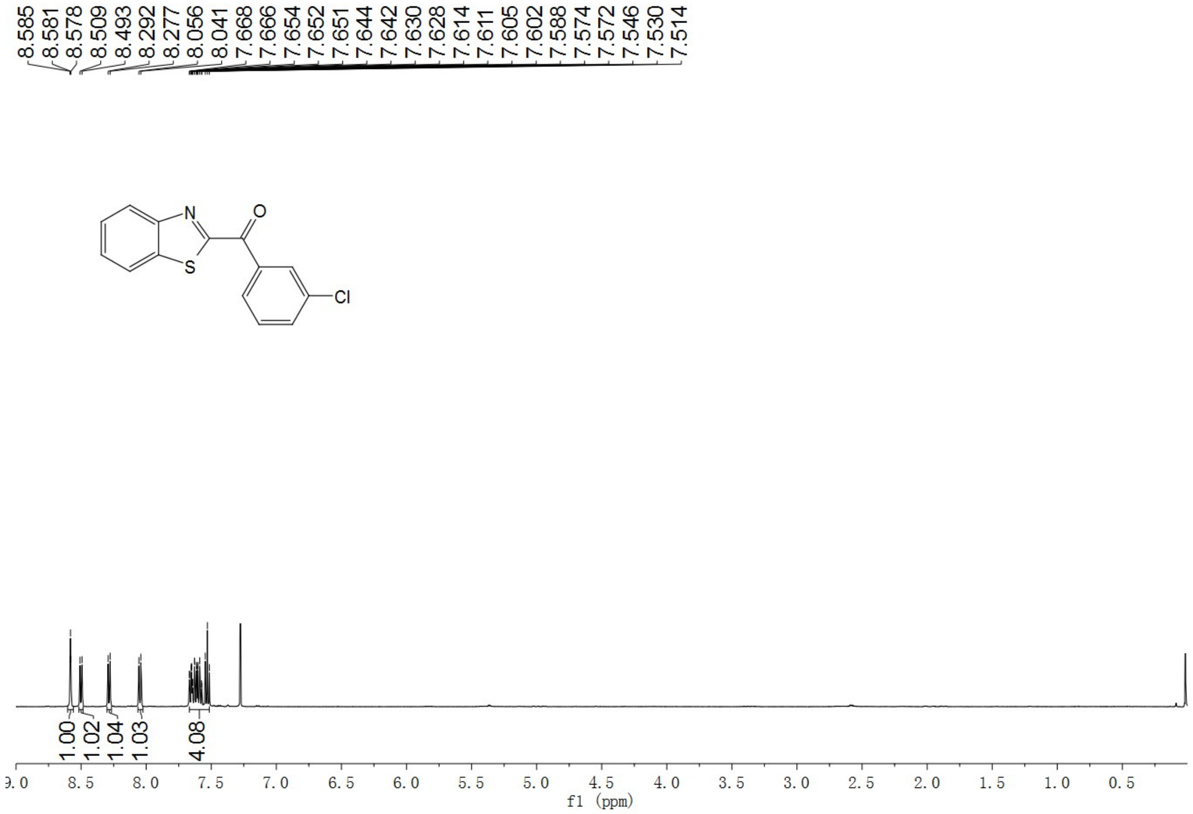

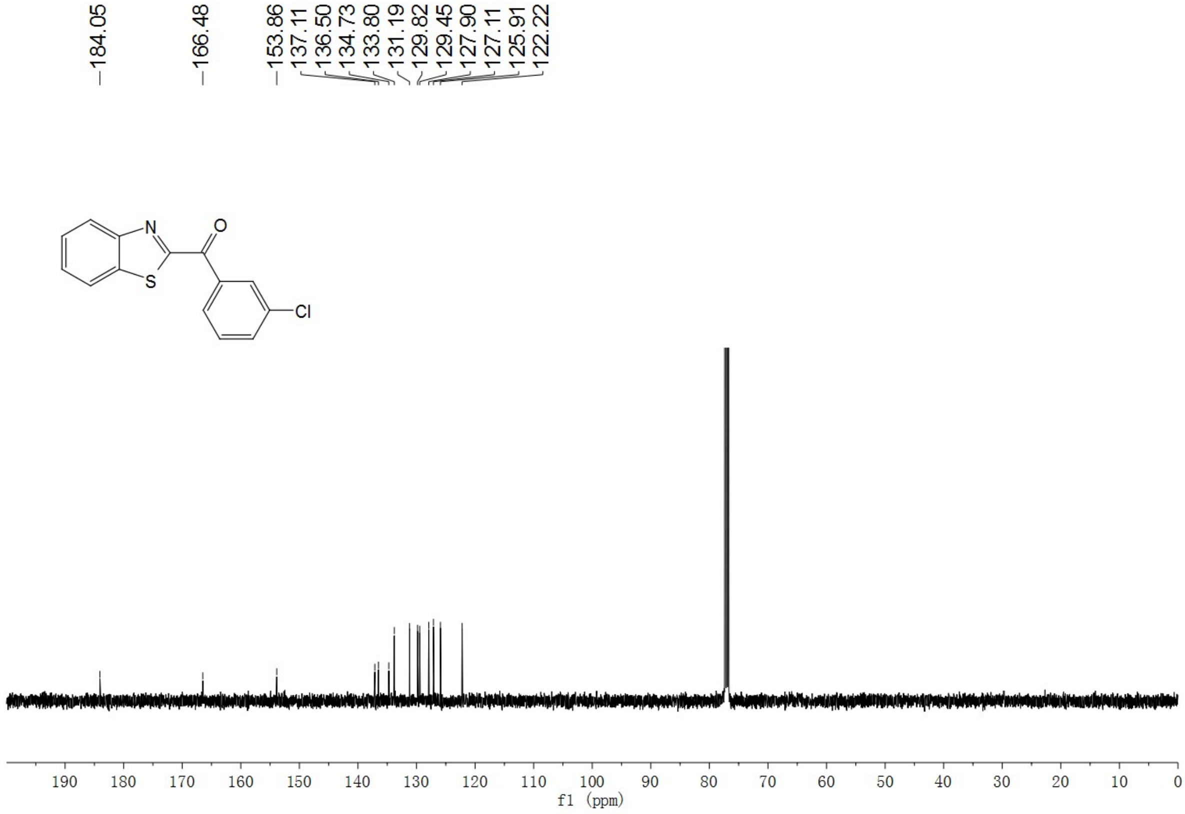

^{1}H and ^{13}C NMR Spectra of 3aj

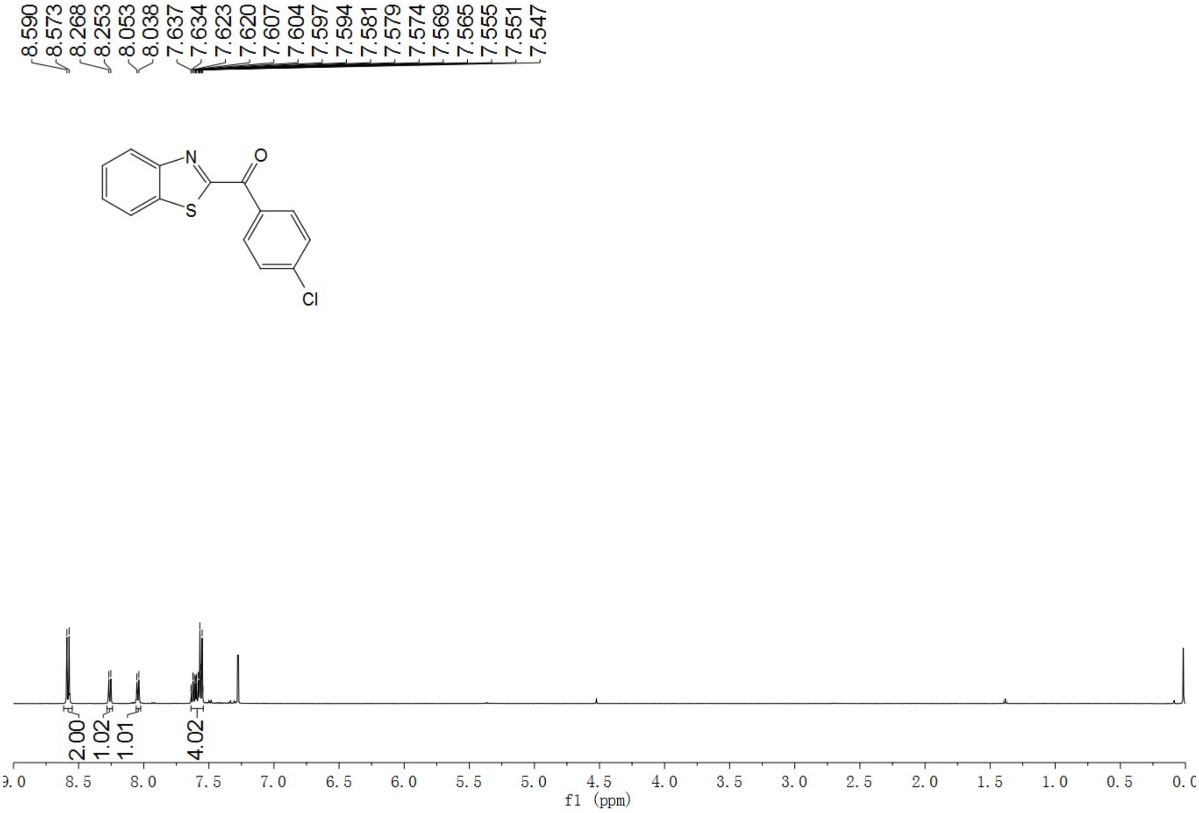

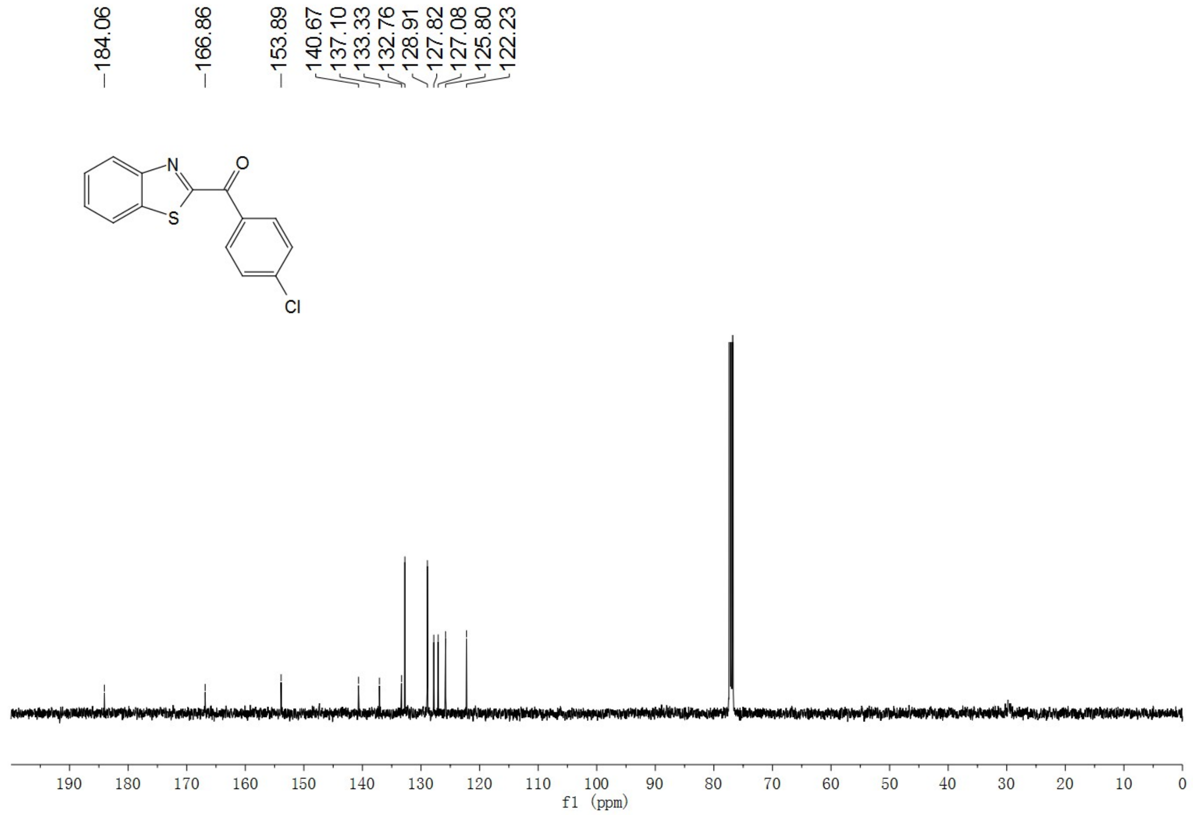

^{1}H and ^{13}C NMR Spectra of 3ak

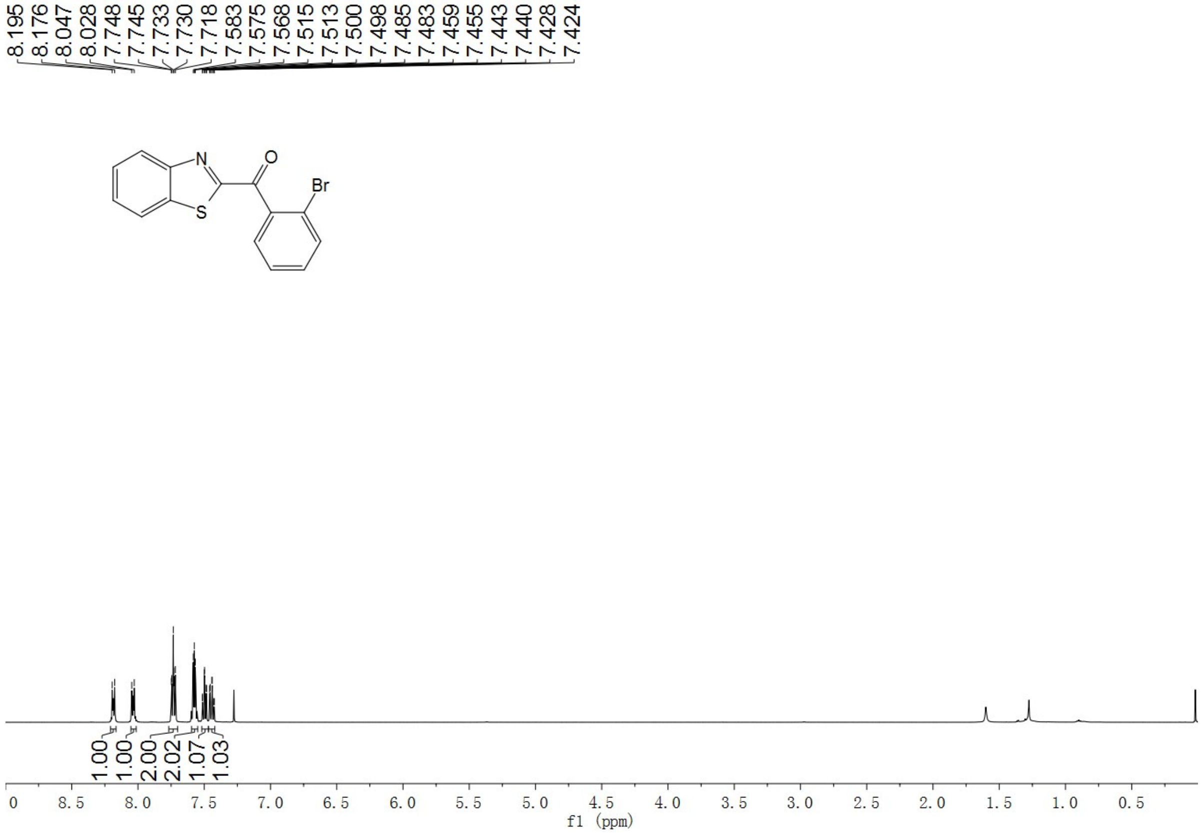

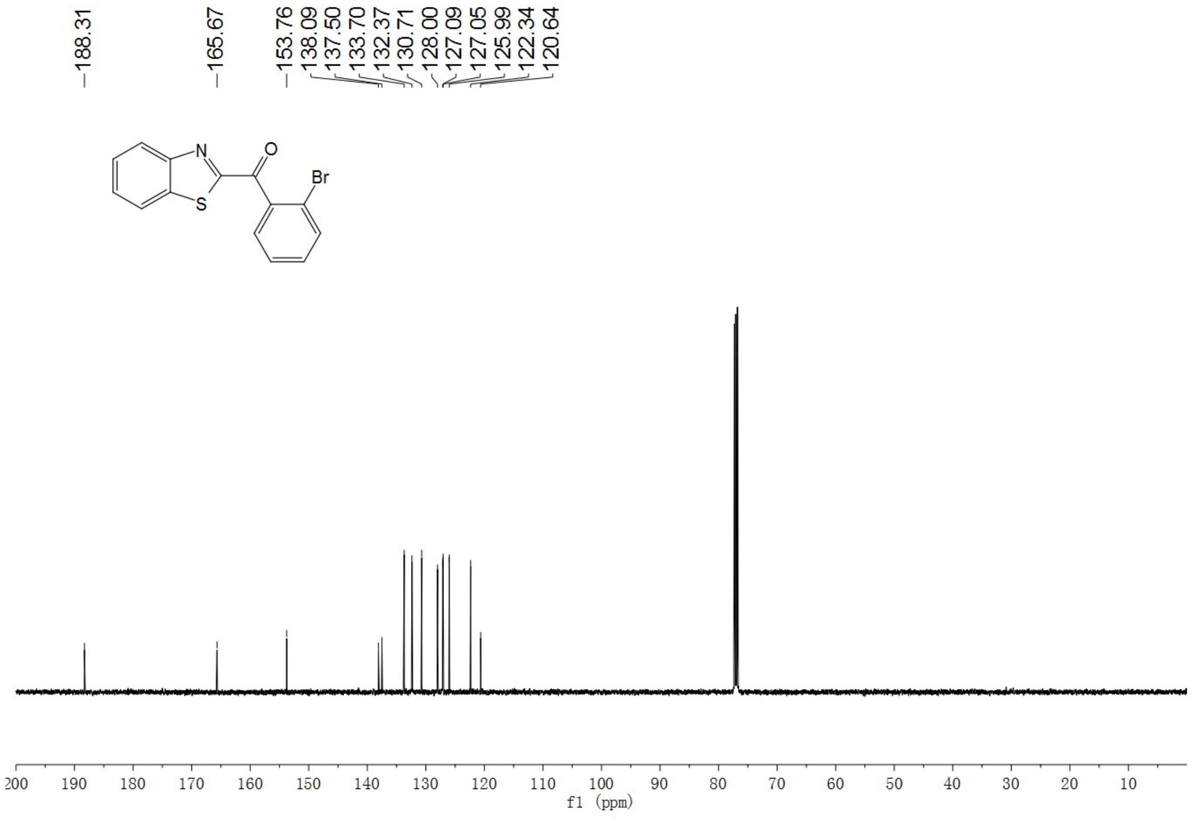

^{1}H and ^{13}C NMR Spectra of 3al

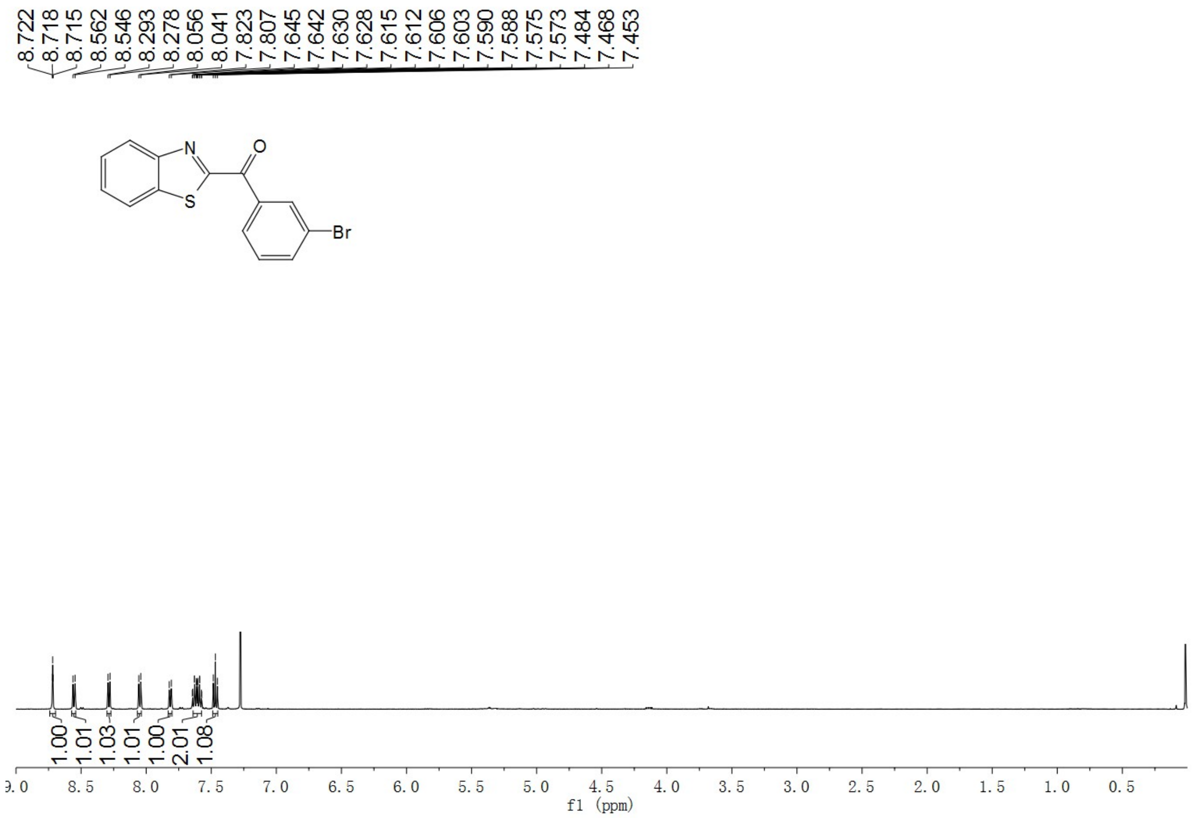

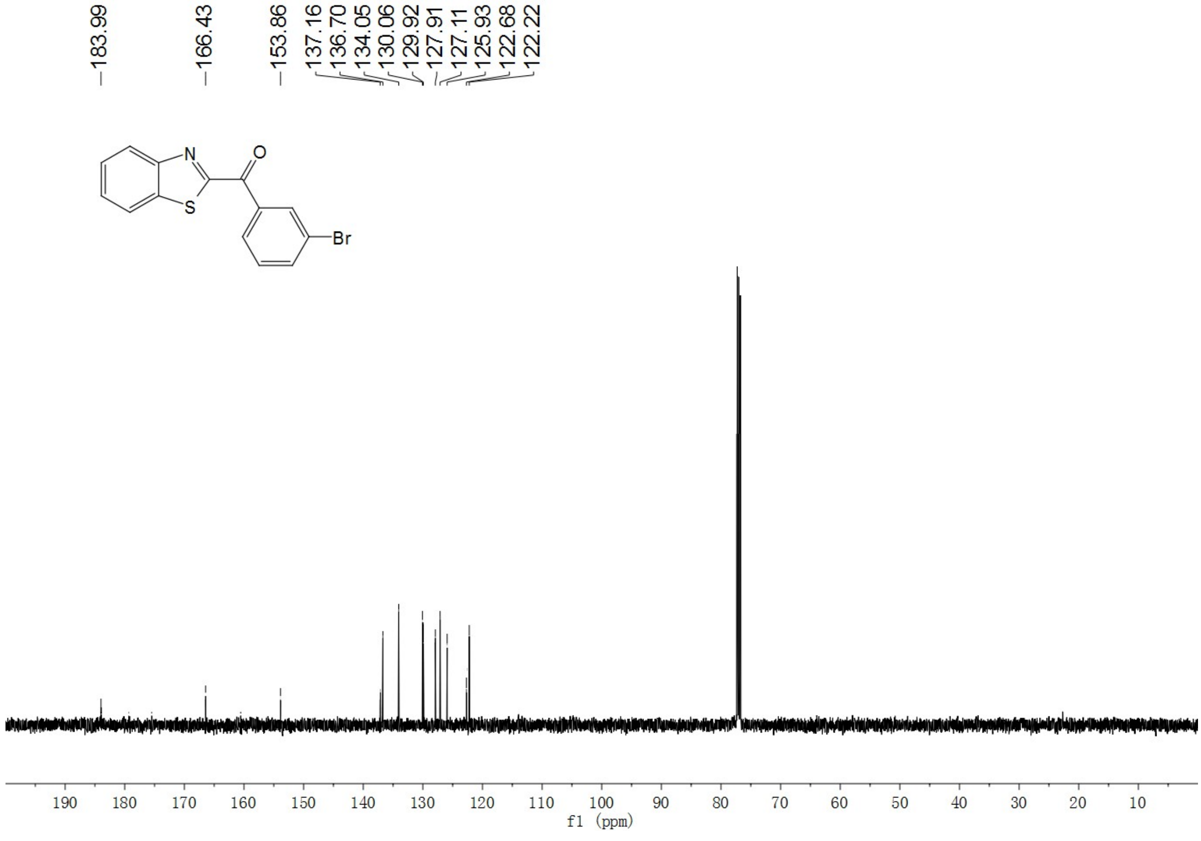

^{1}H and ^{13}C NMR Spectra of 3am

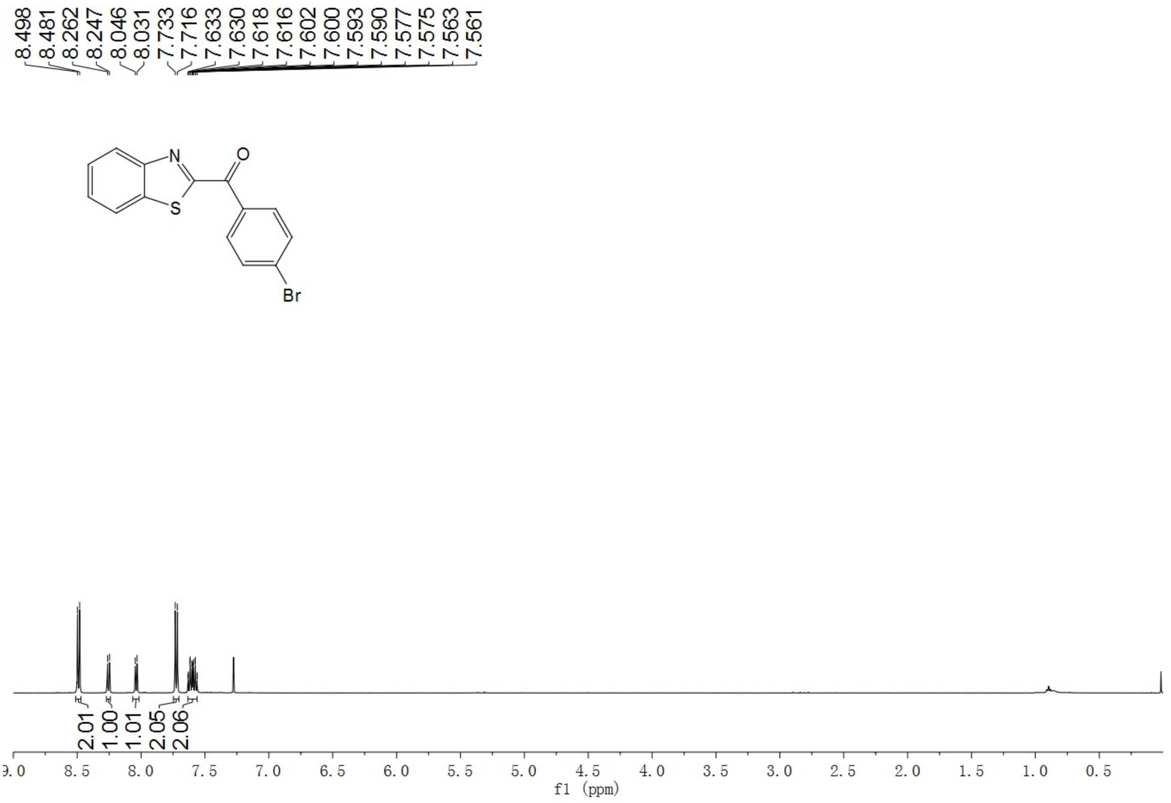

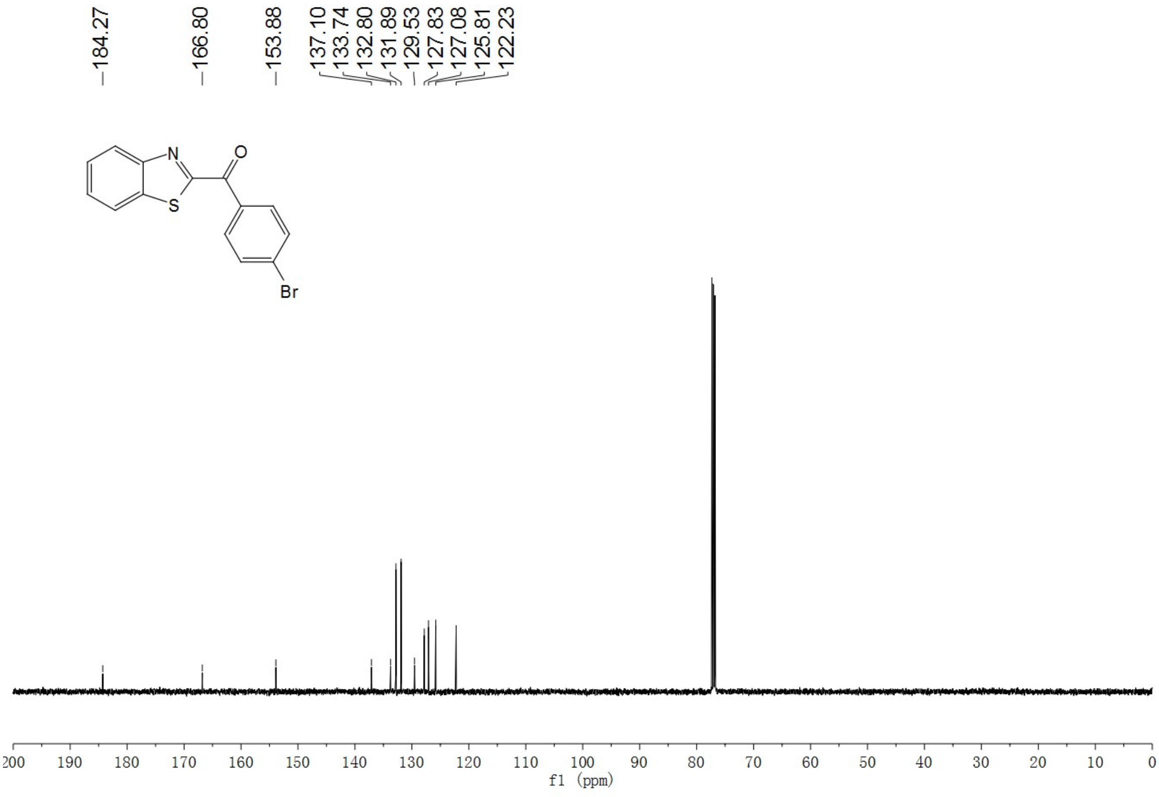

^{1}H and ^{13}C NMR Spectra of 3an

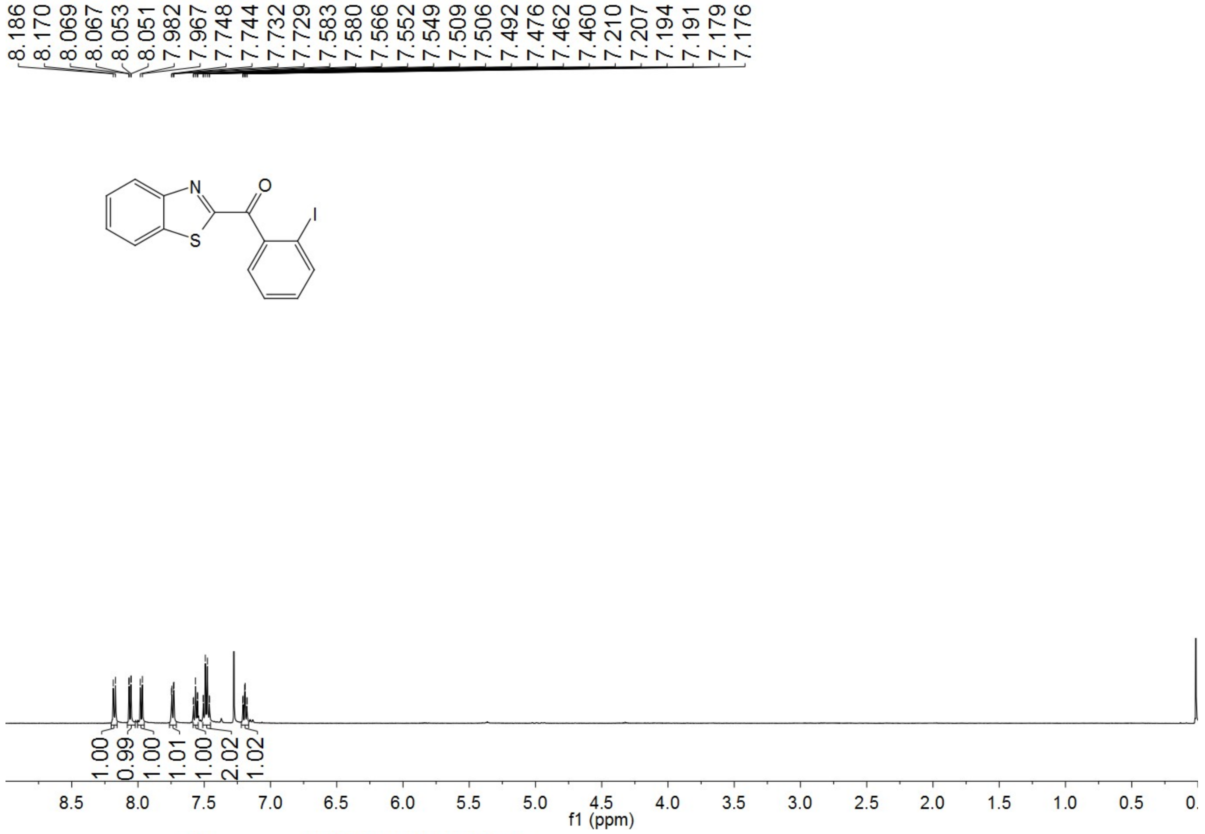

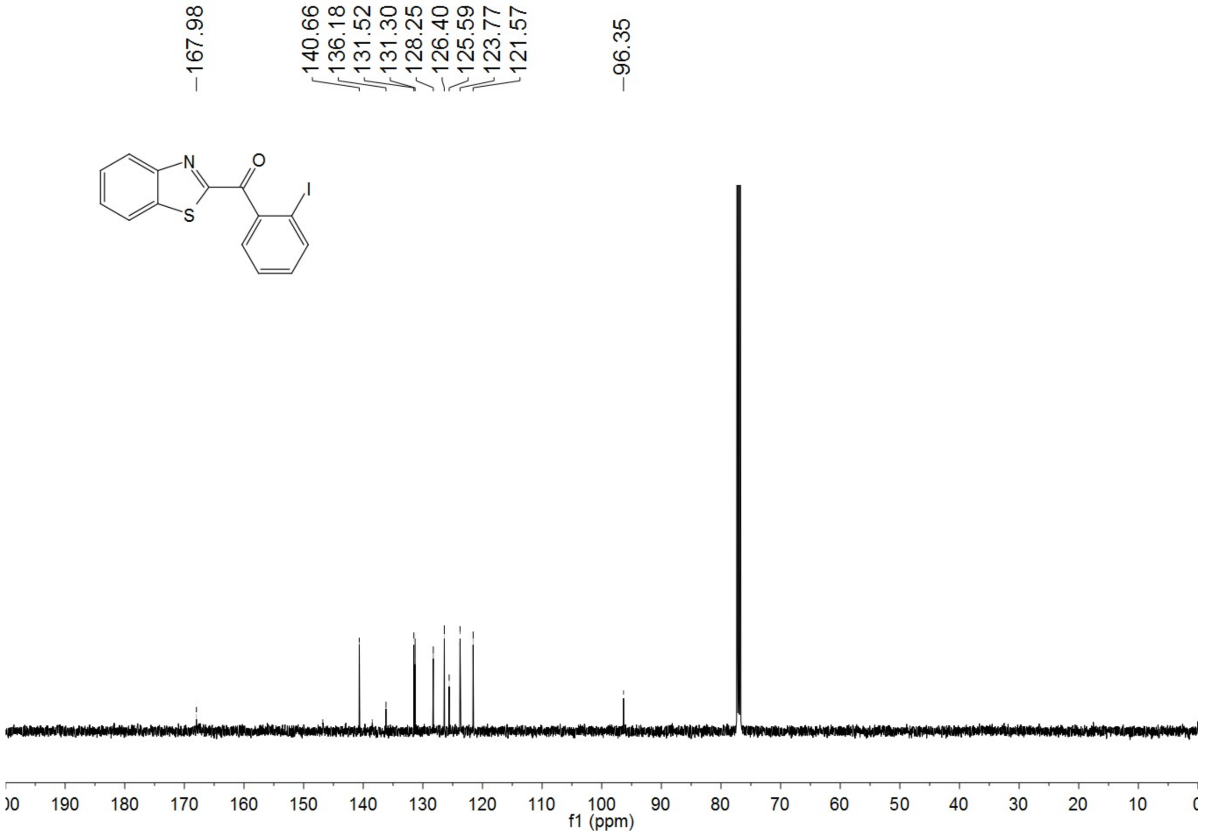

^{1}H and ^{13}C NMR Spectra of 3ao

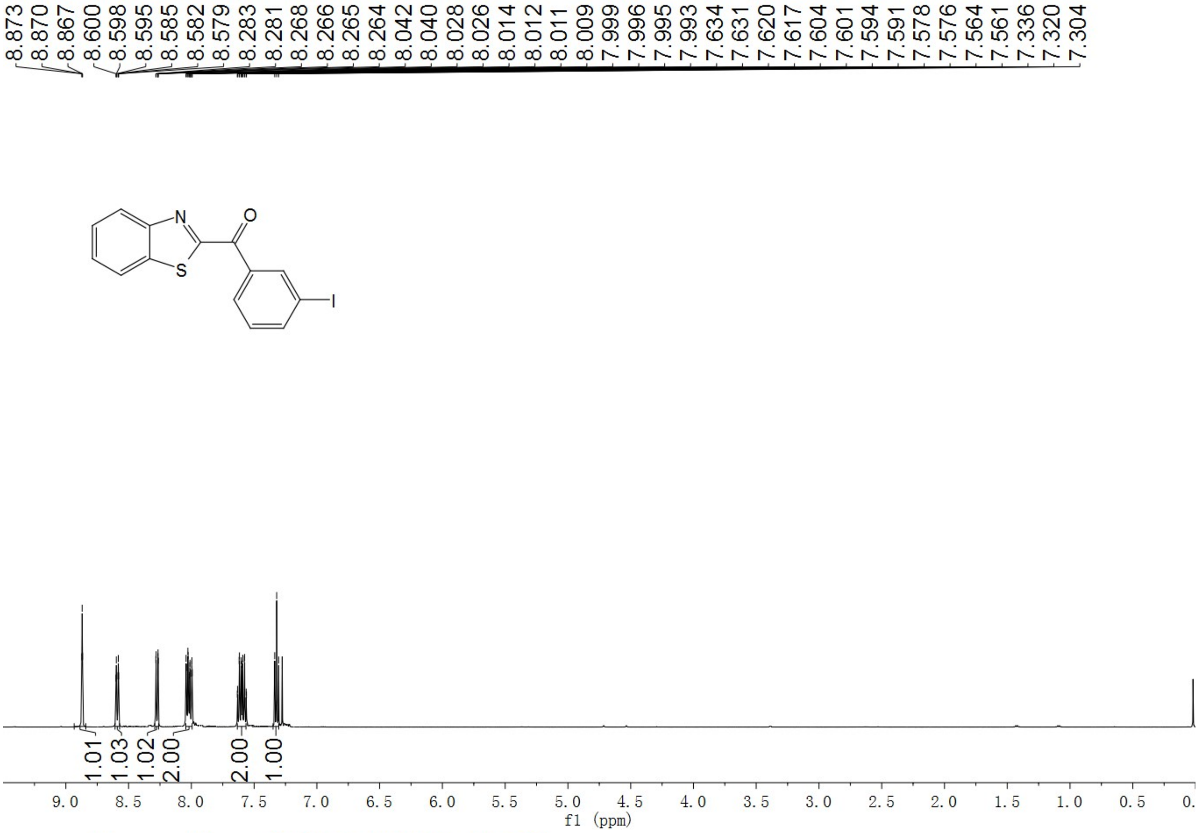

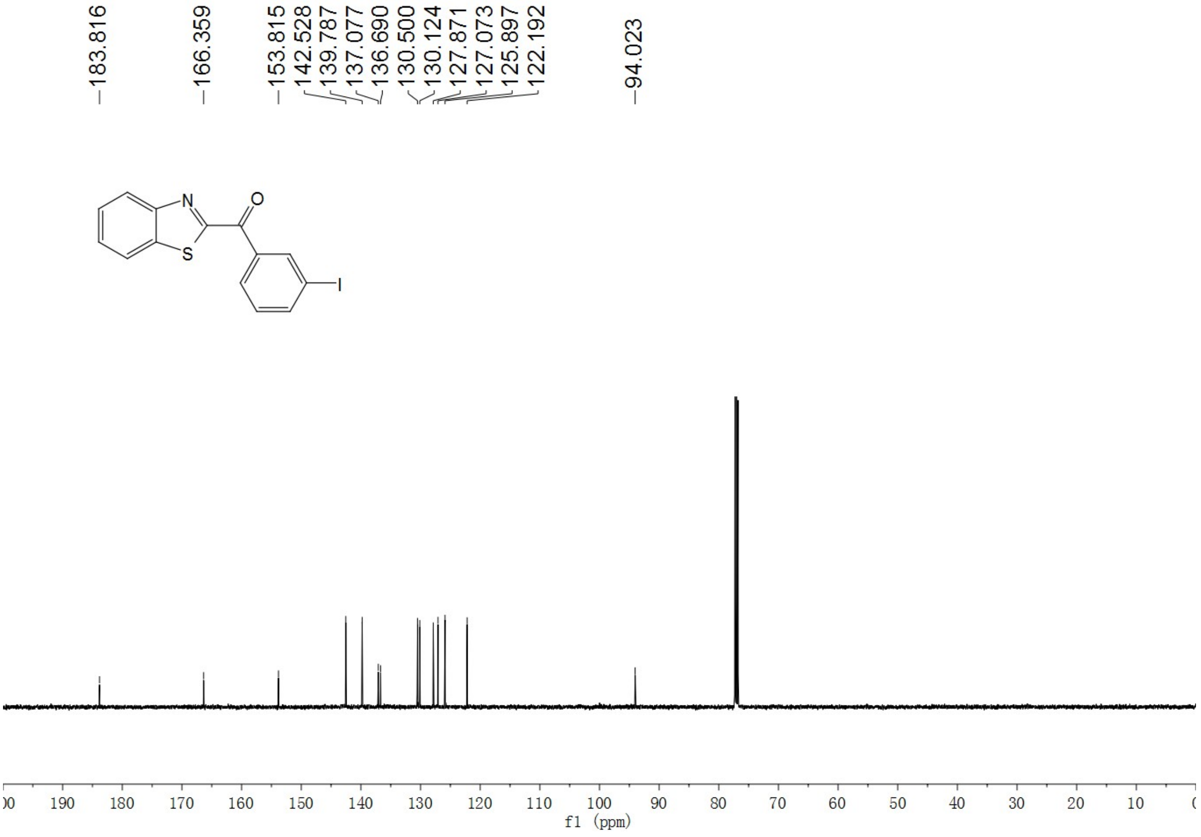

^{1}H and ^{13}C NMR Spectra of 3ap

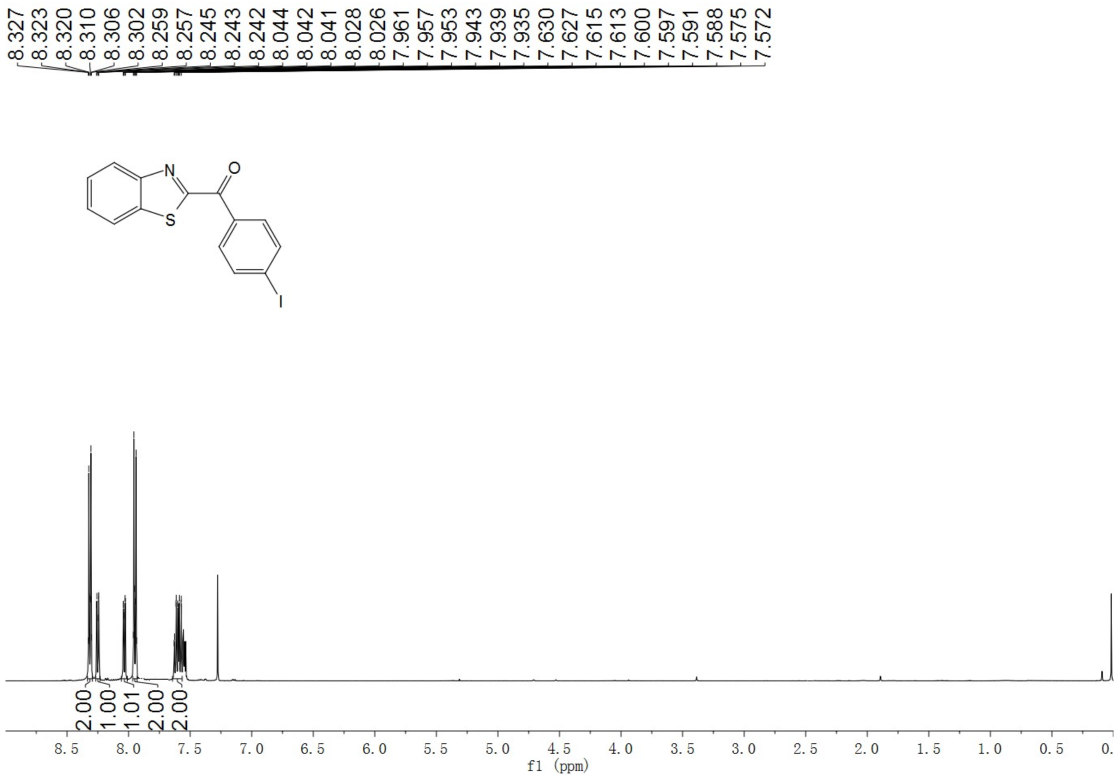

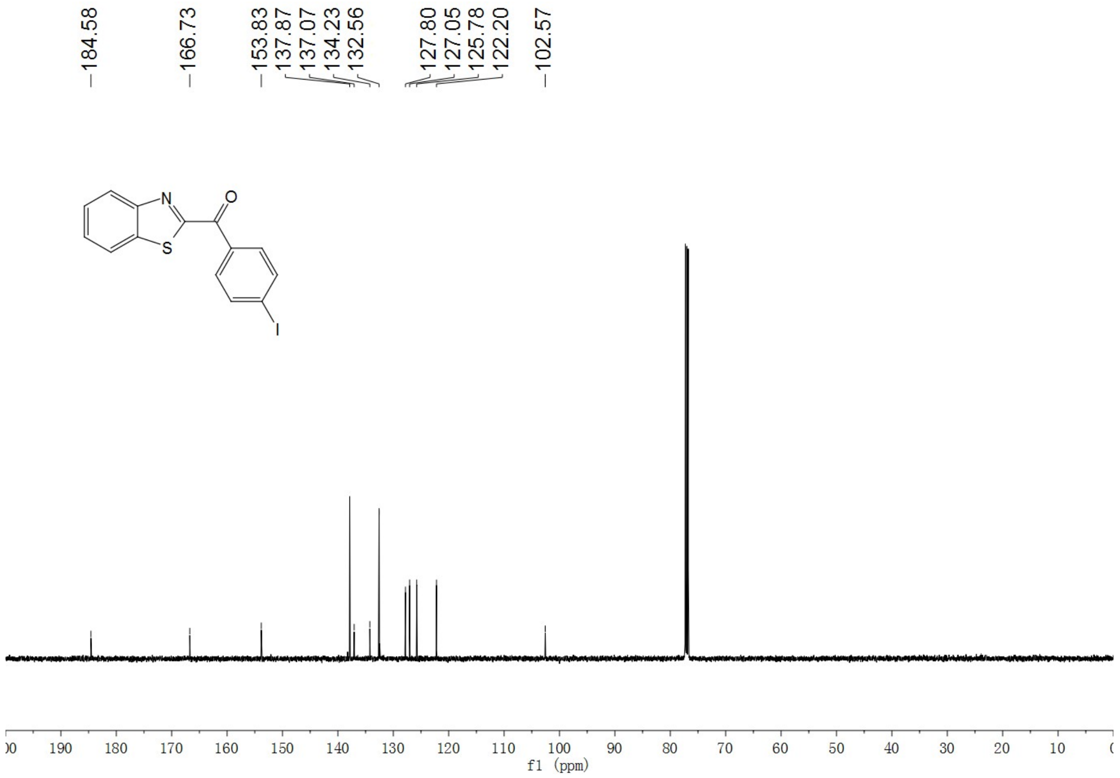

^{1}H and ^{13}C NMR Spectra of 3aq

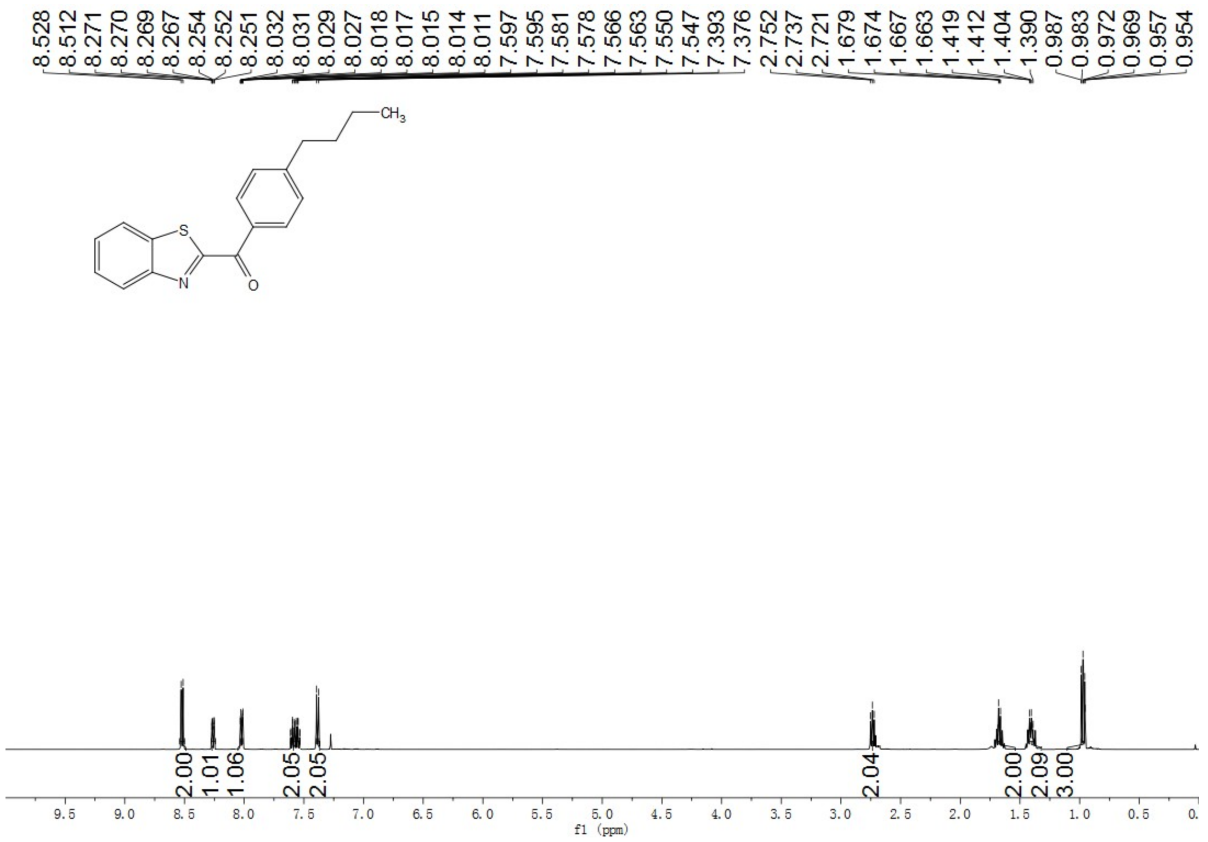

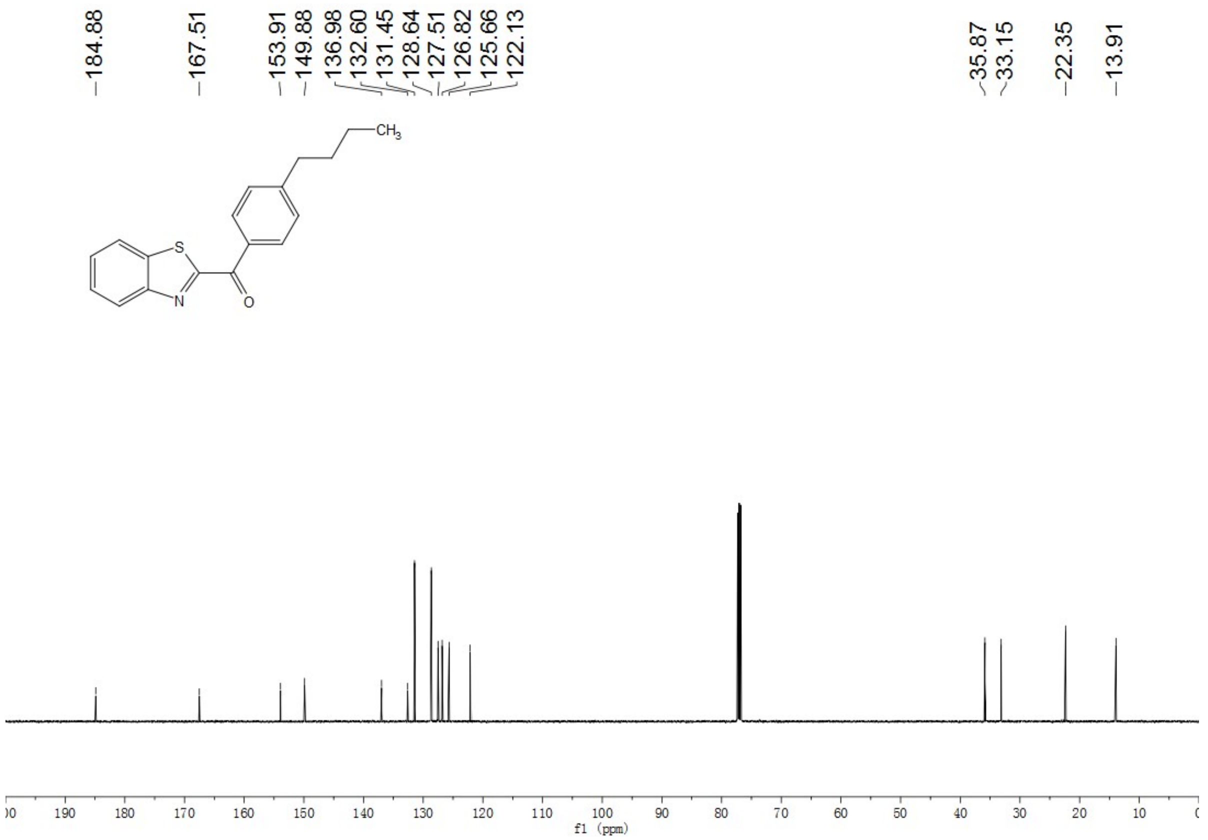

^{1}H and ^{13}C NMR Spectra of 3ar

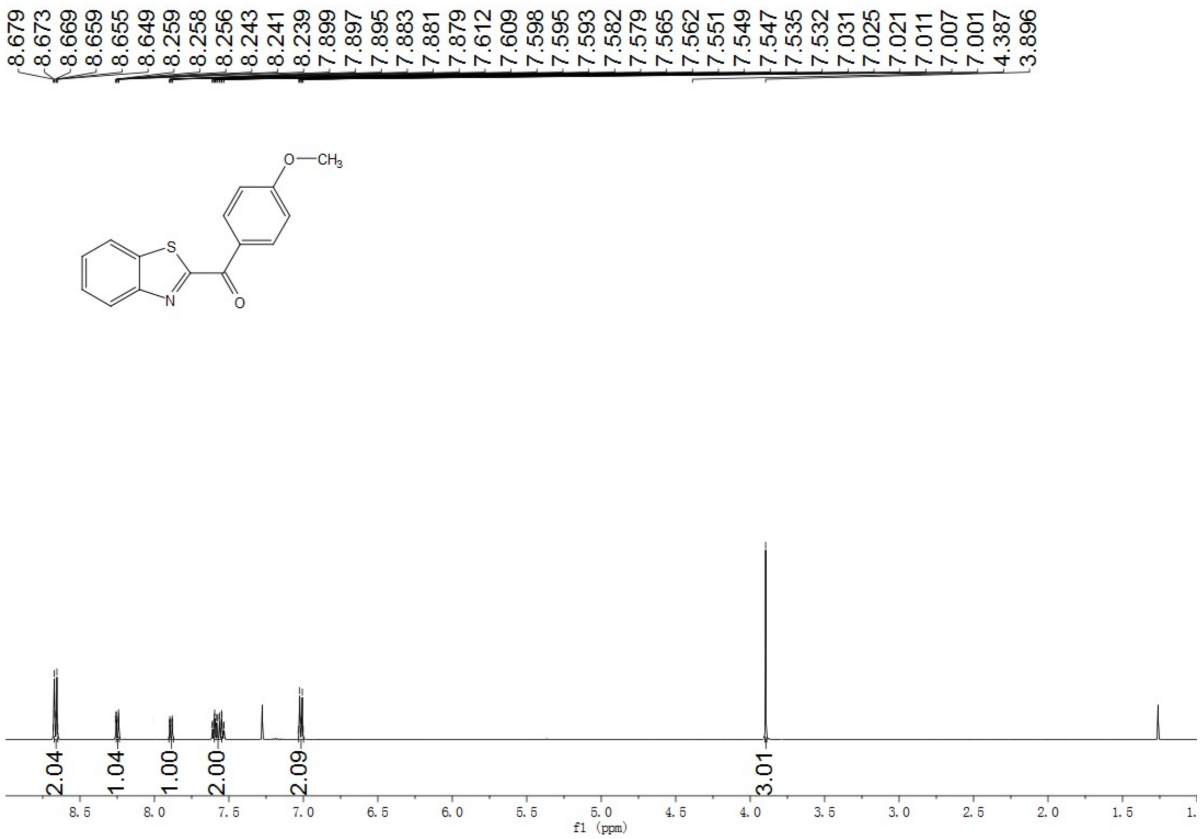

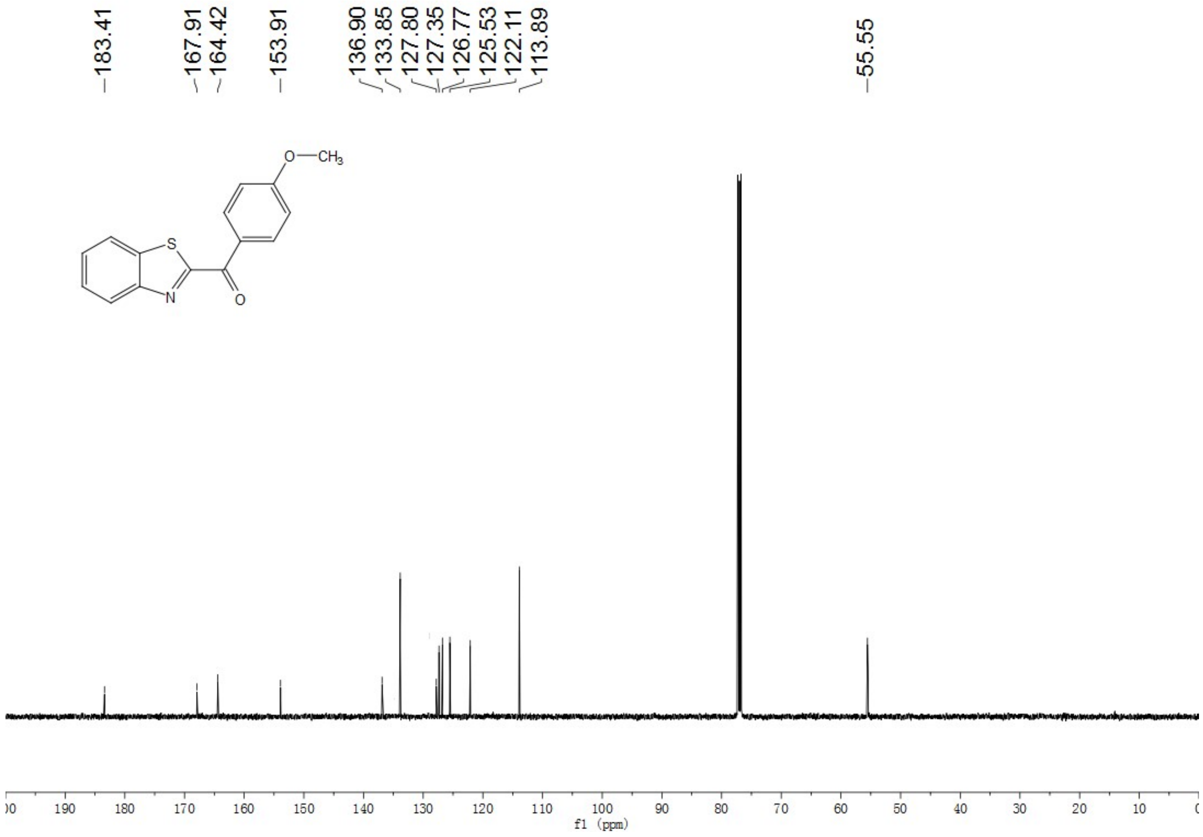

^{1}H and ^{13}C NMR Spectra of 3as

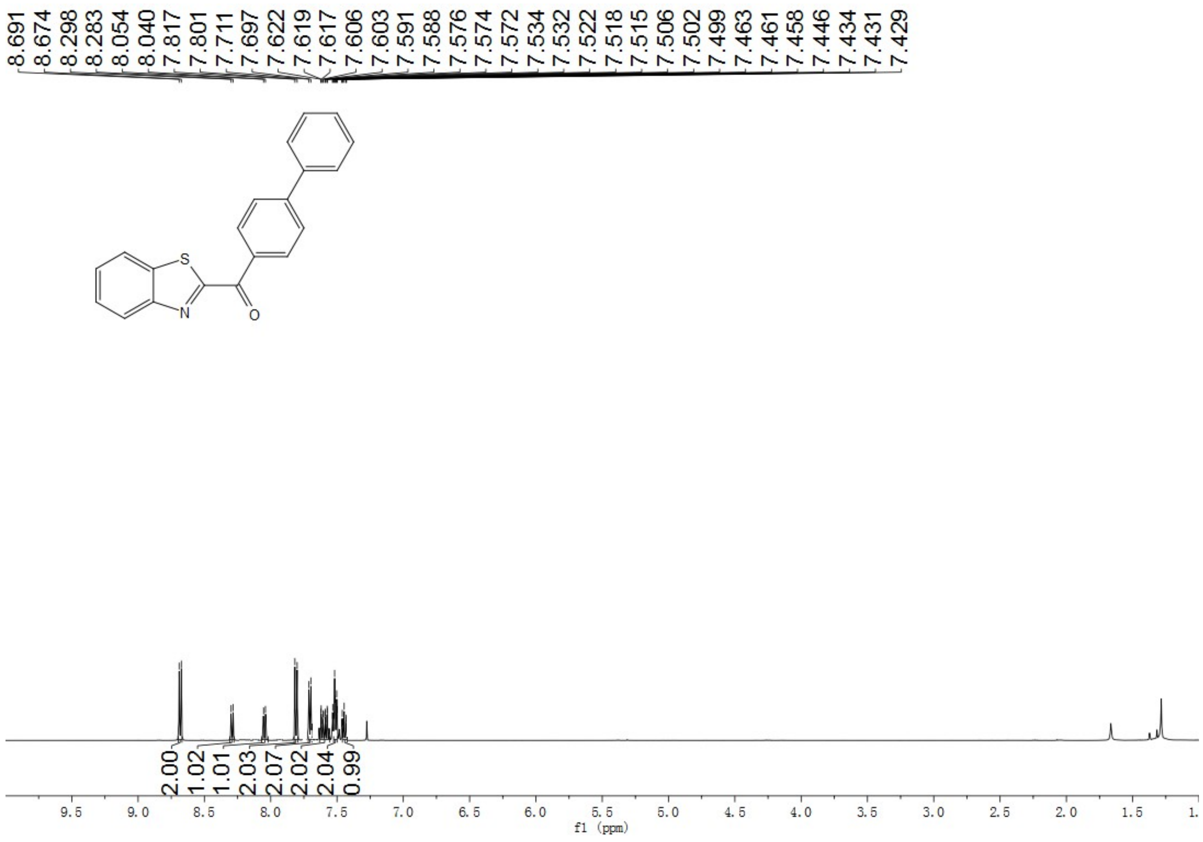

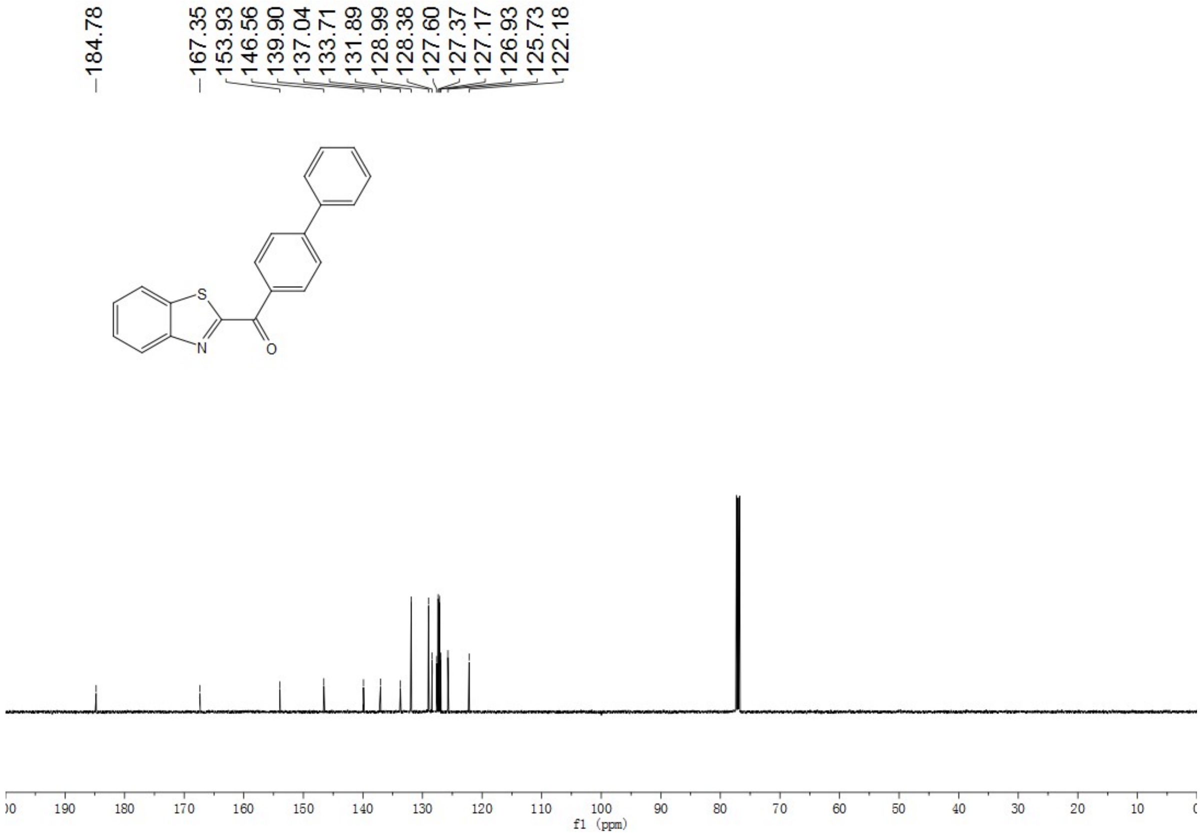

^{1}H and ^{13}C NMR Spectra of 3at

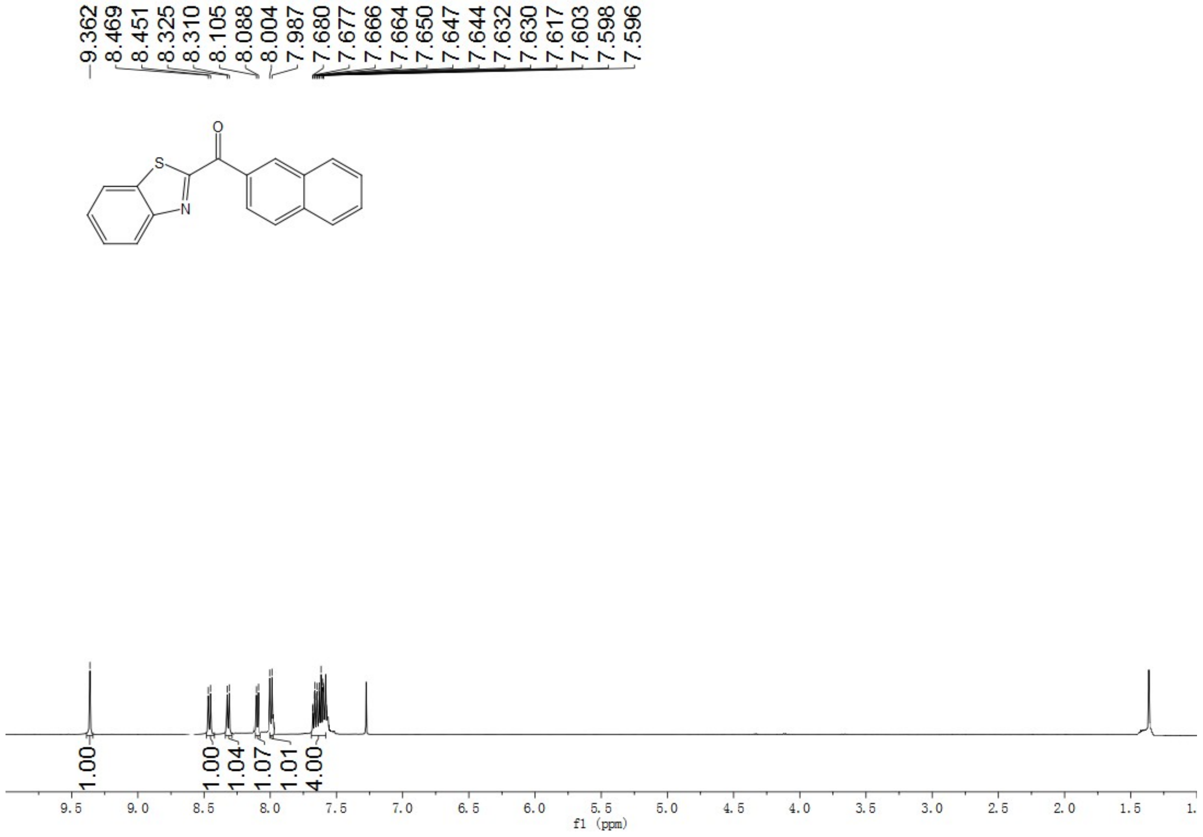

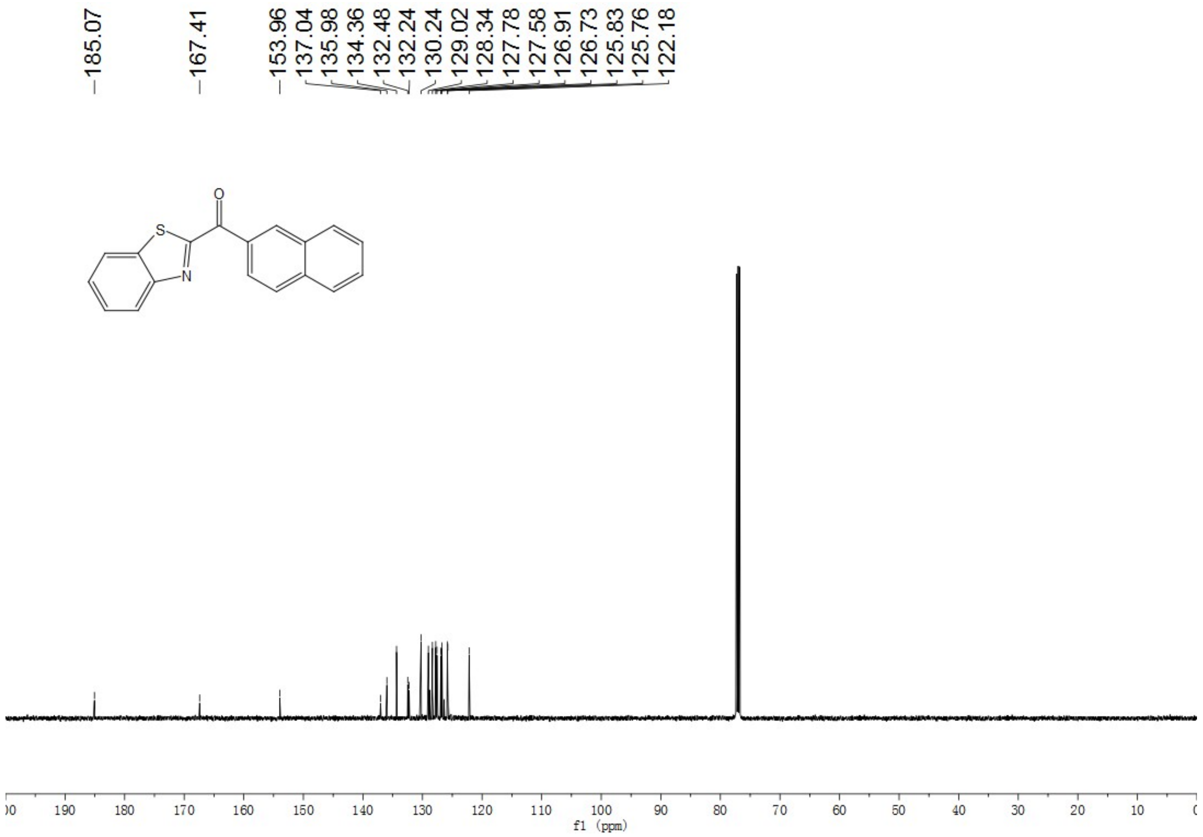

^{1}H and ^{13}C NMR Spectra of 3ba

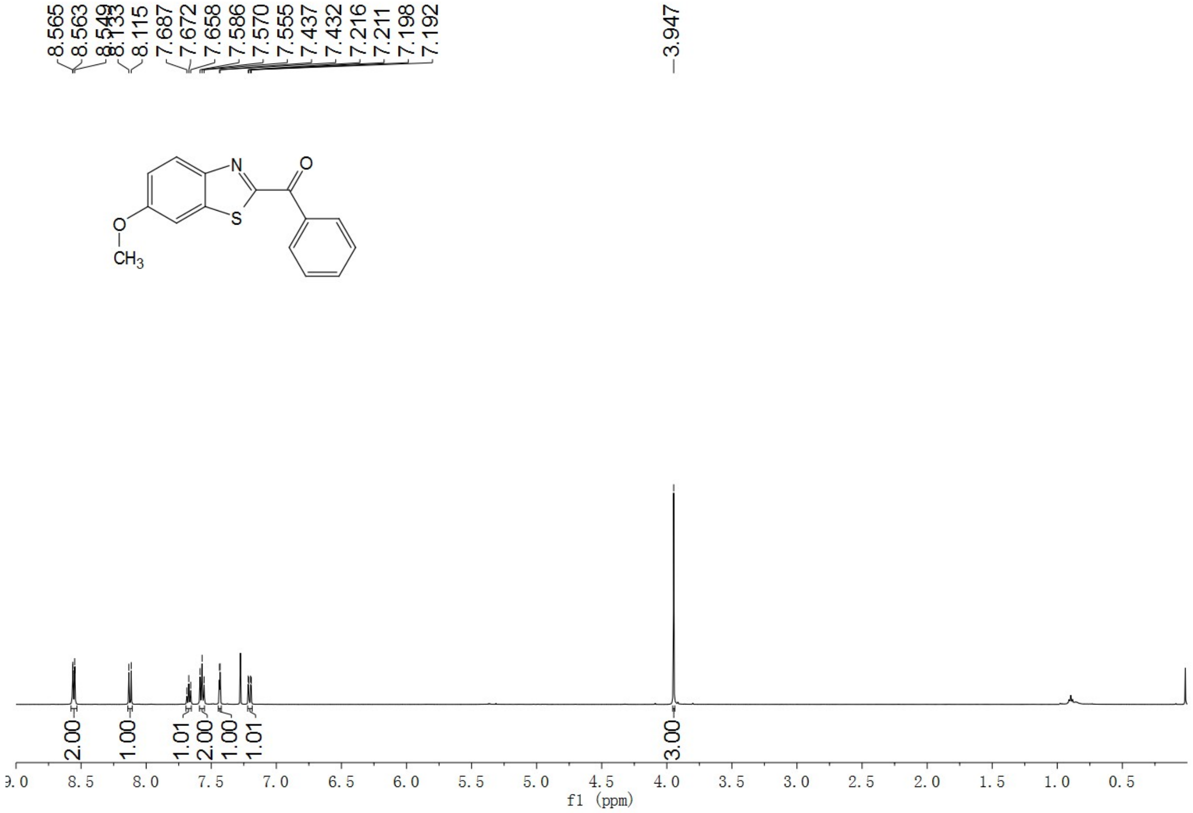

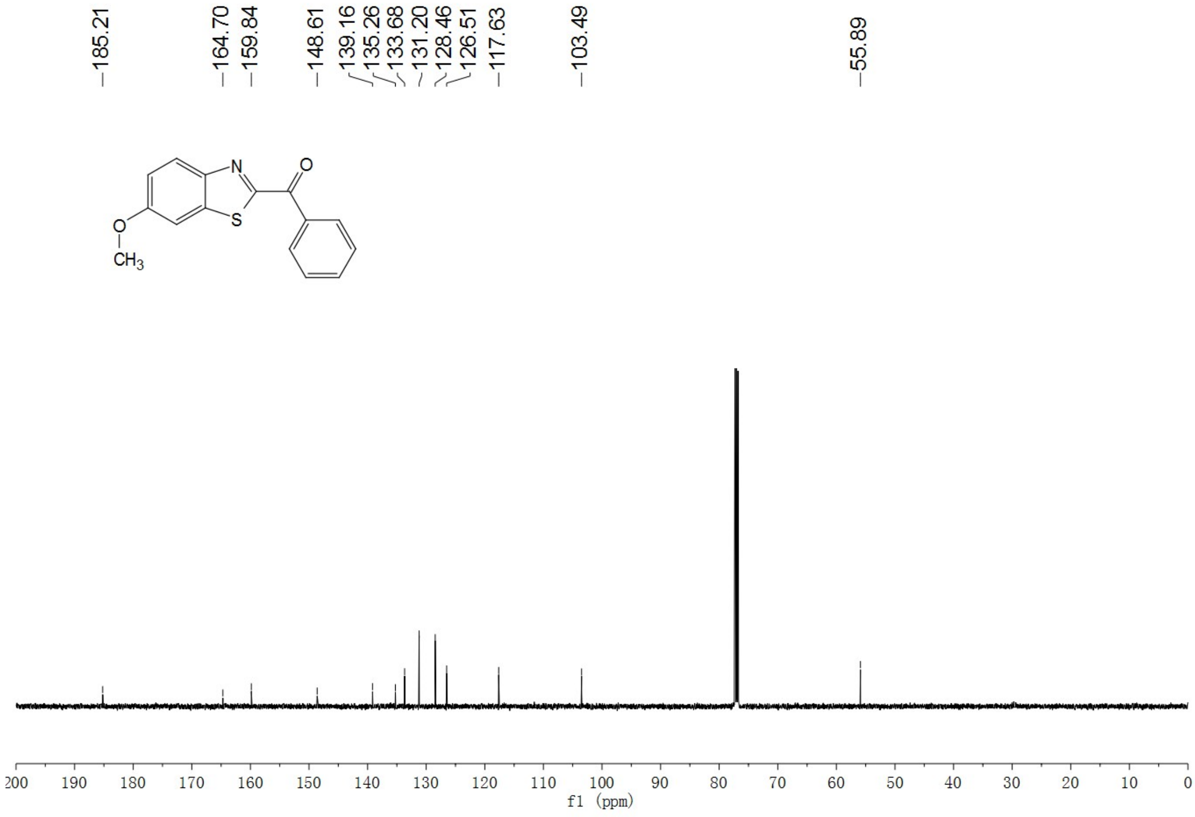

^{1}H and ^{13}C NMR Spectra of 3bb

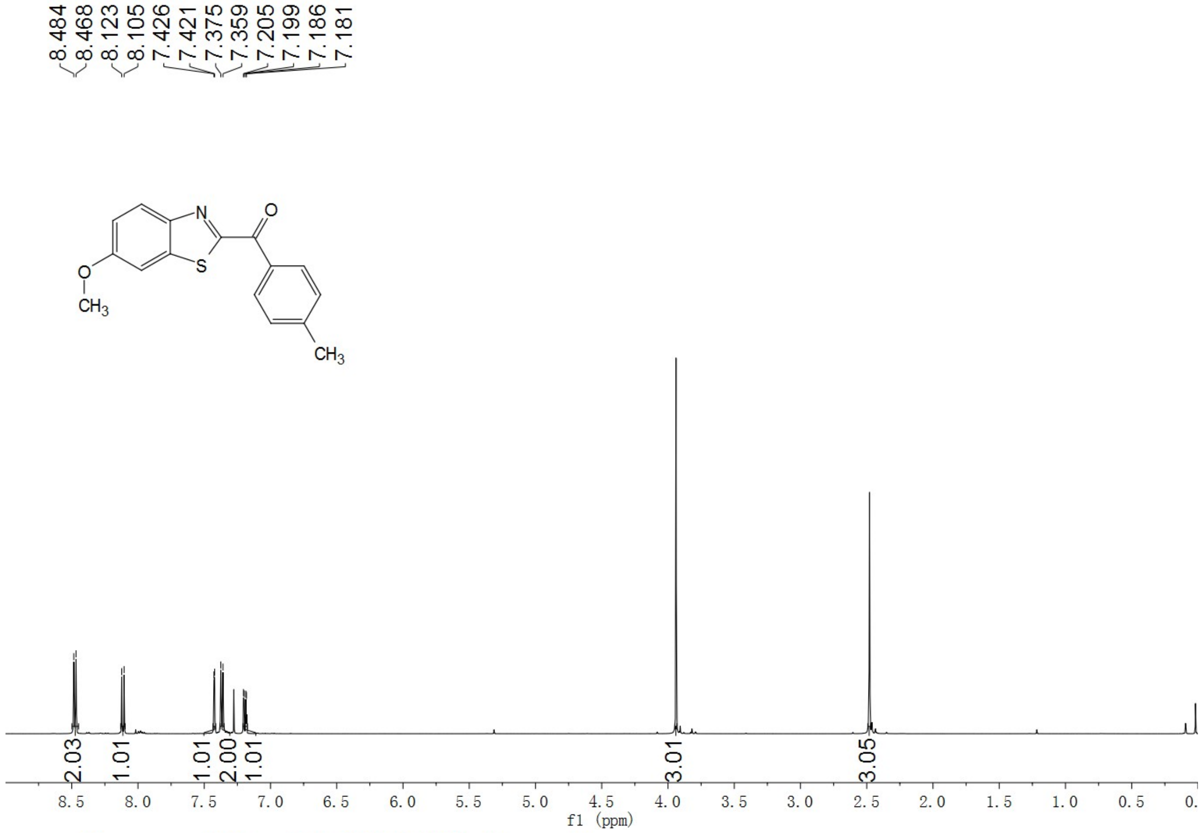

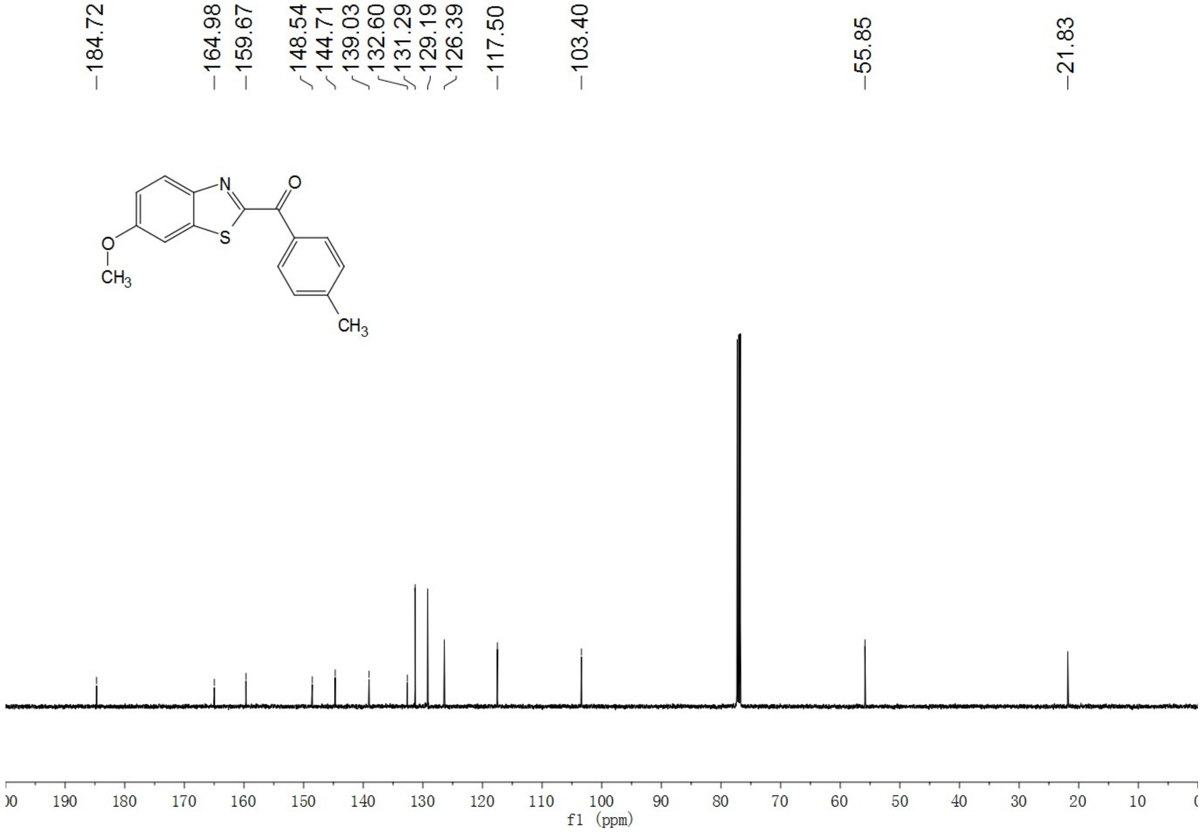

^{1}H and ^{13}C NMR Spectra of 3bc

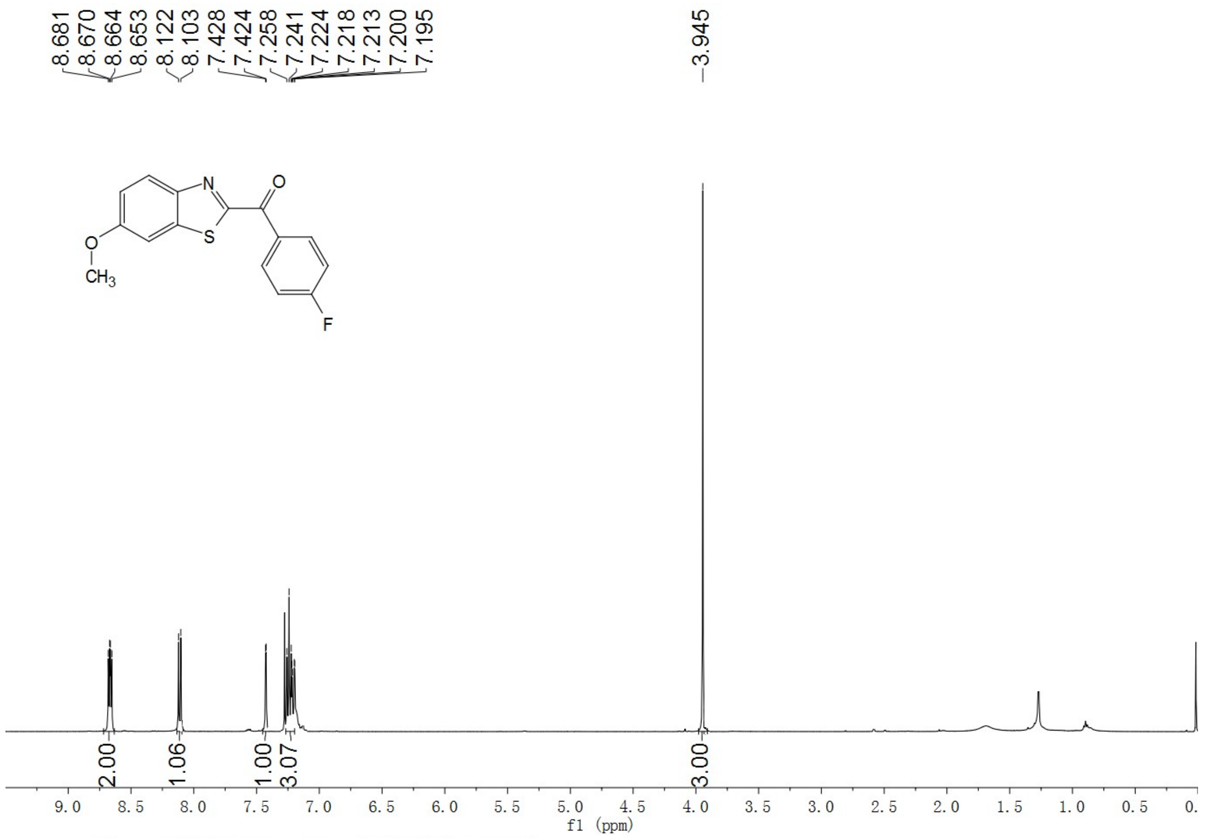

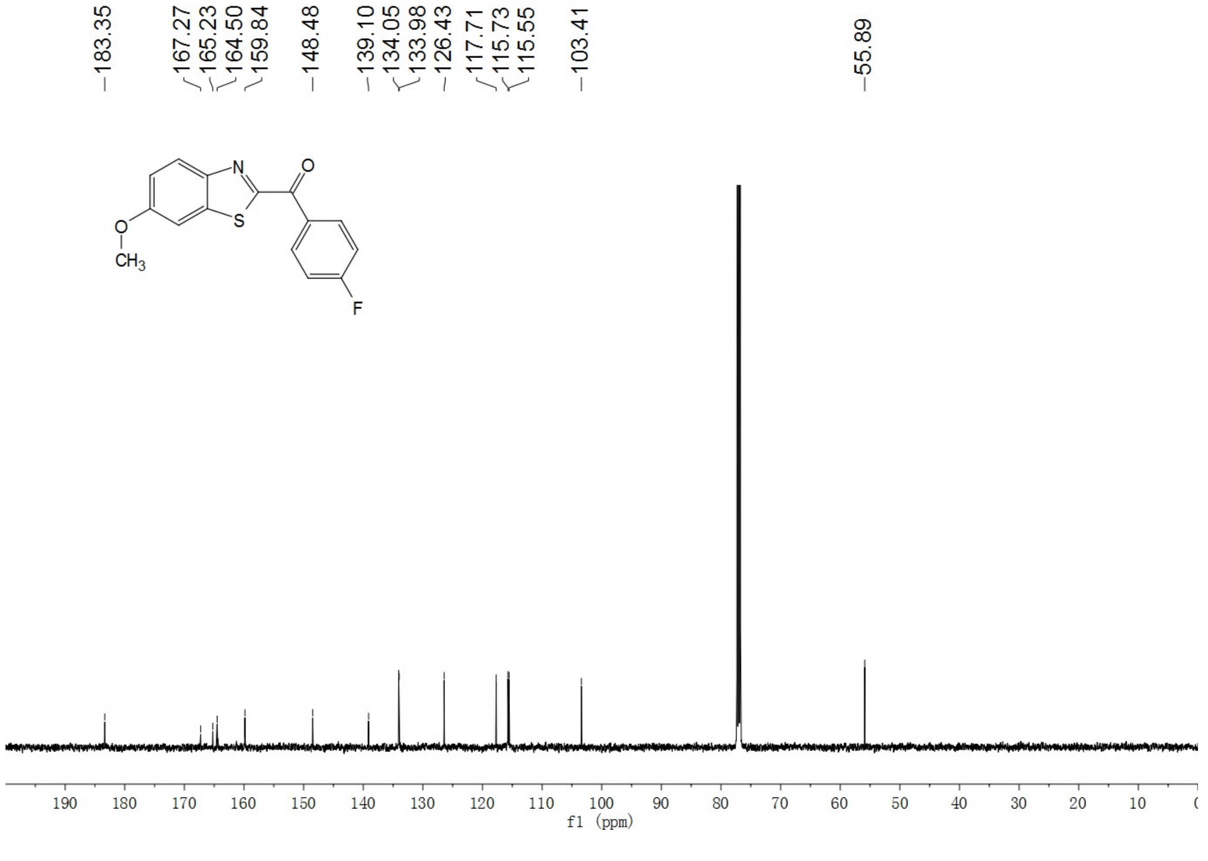

^{1}H and ^{13}C NMR Spectra of 3bd

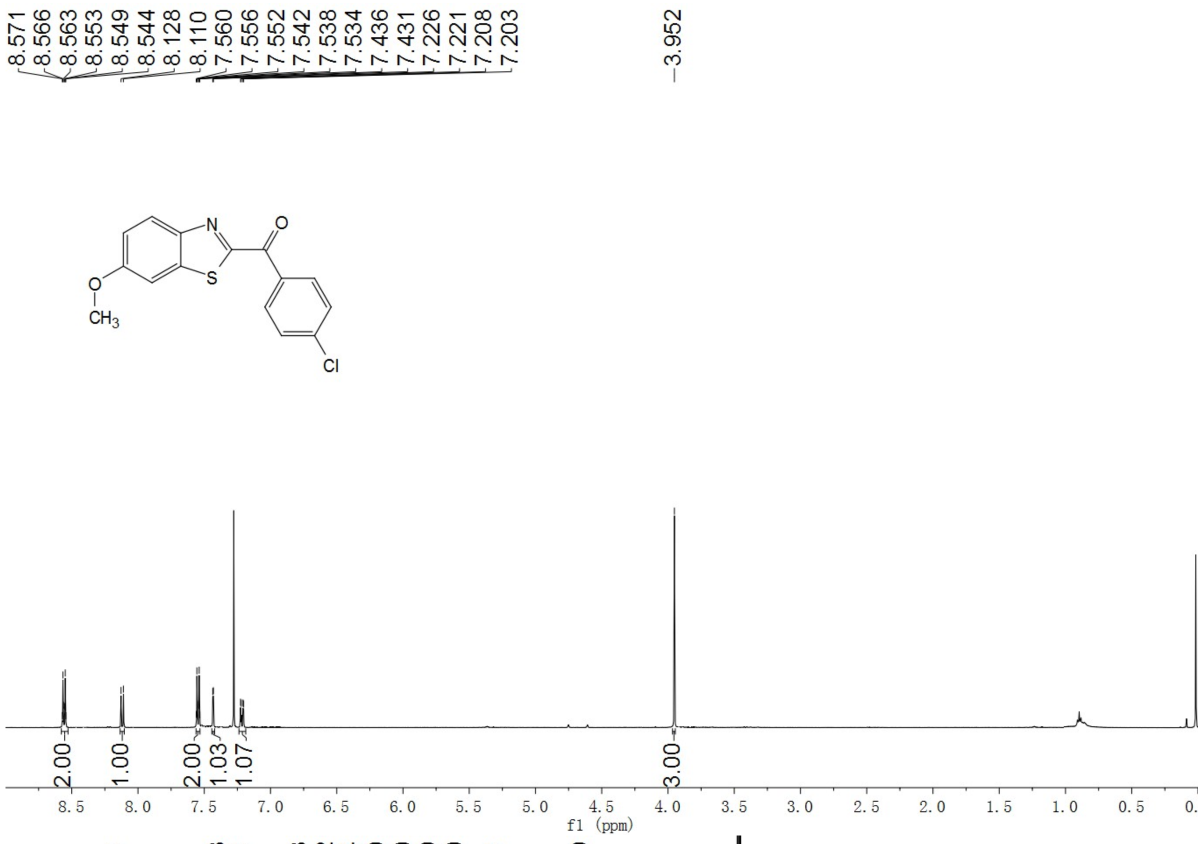

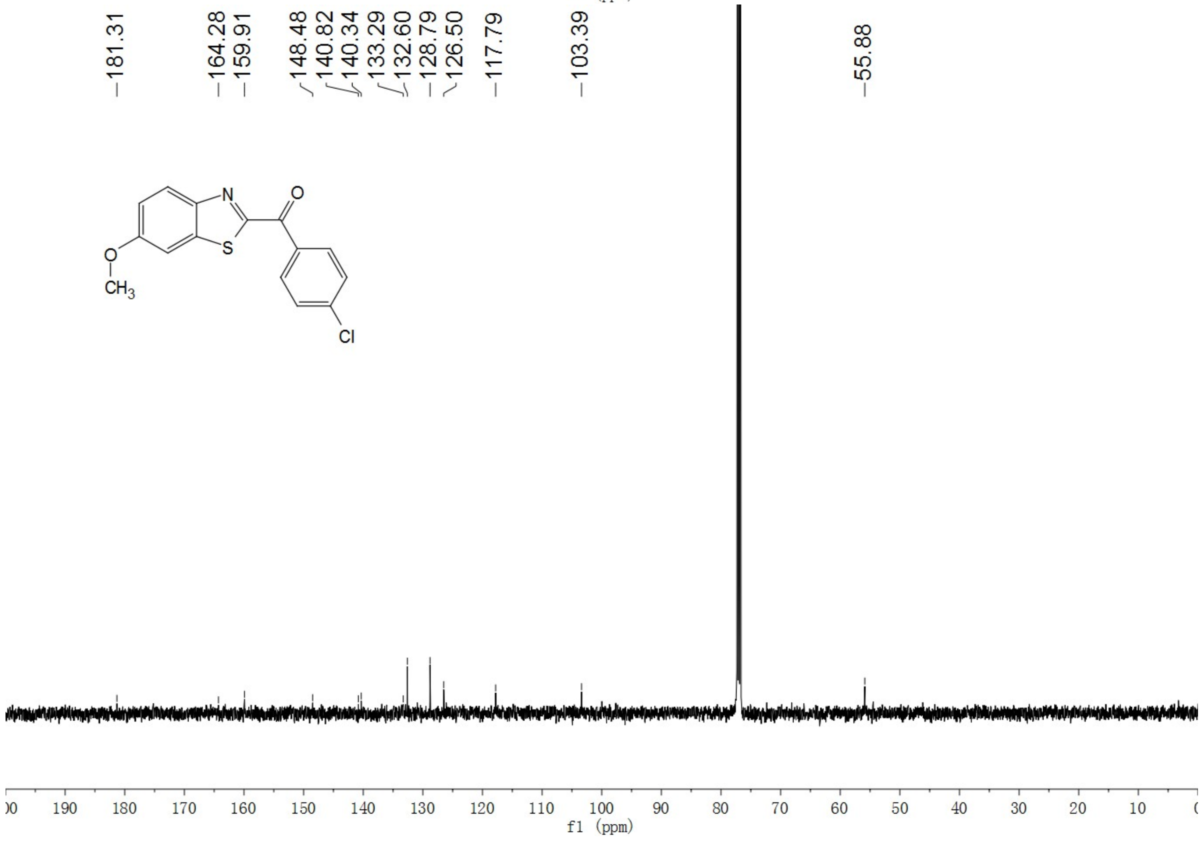

^{1}H and ^{13}C NMR Spectra of 3be

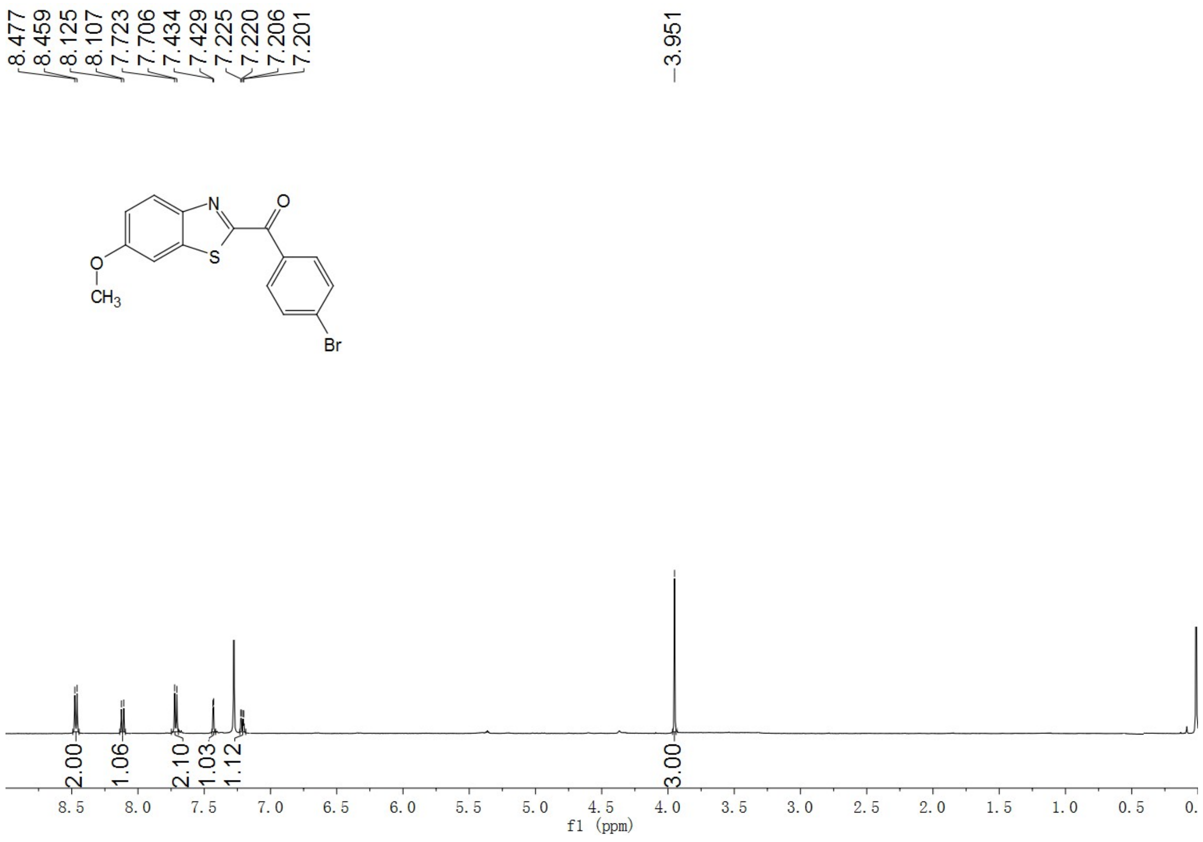

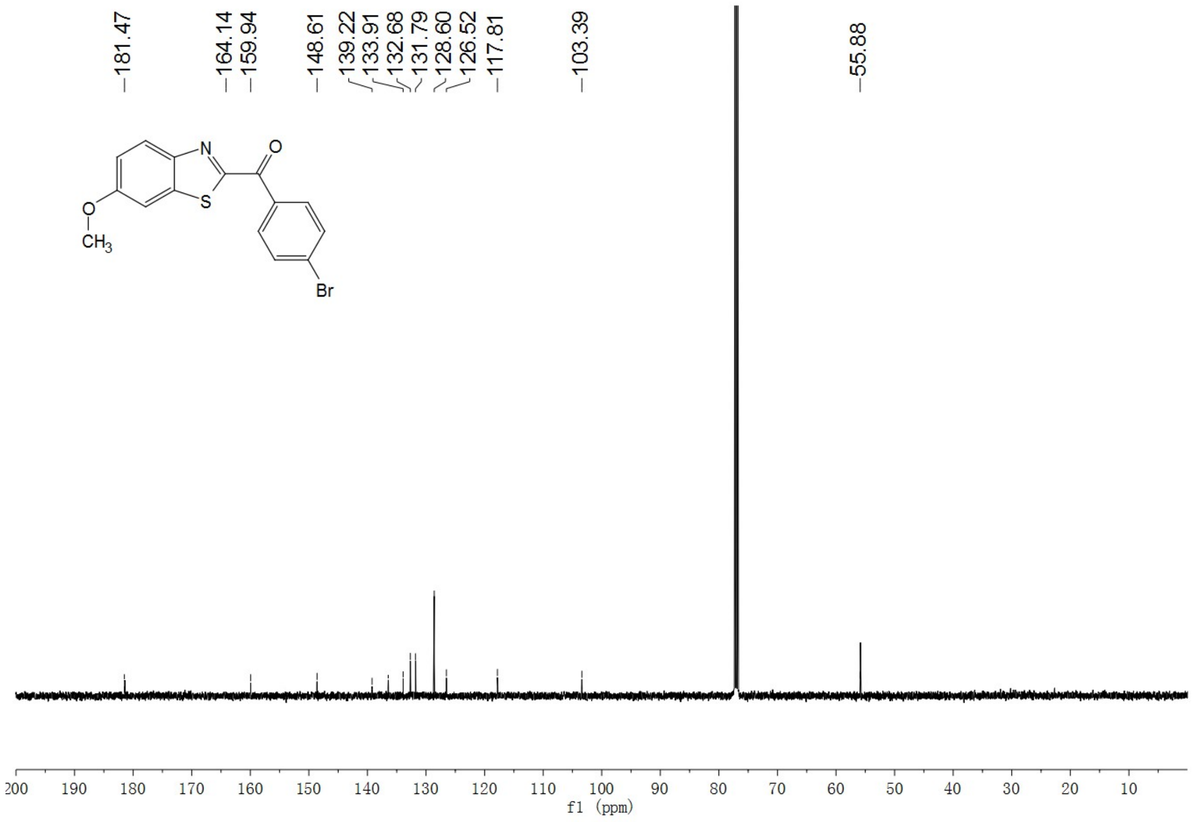

^{1}H and ^{13}C NMR Spectra of 3bf

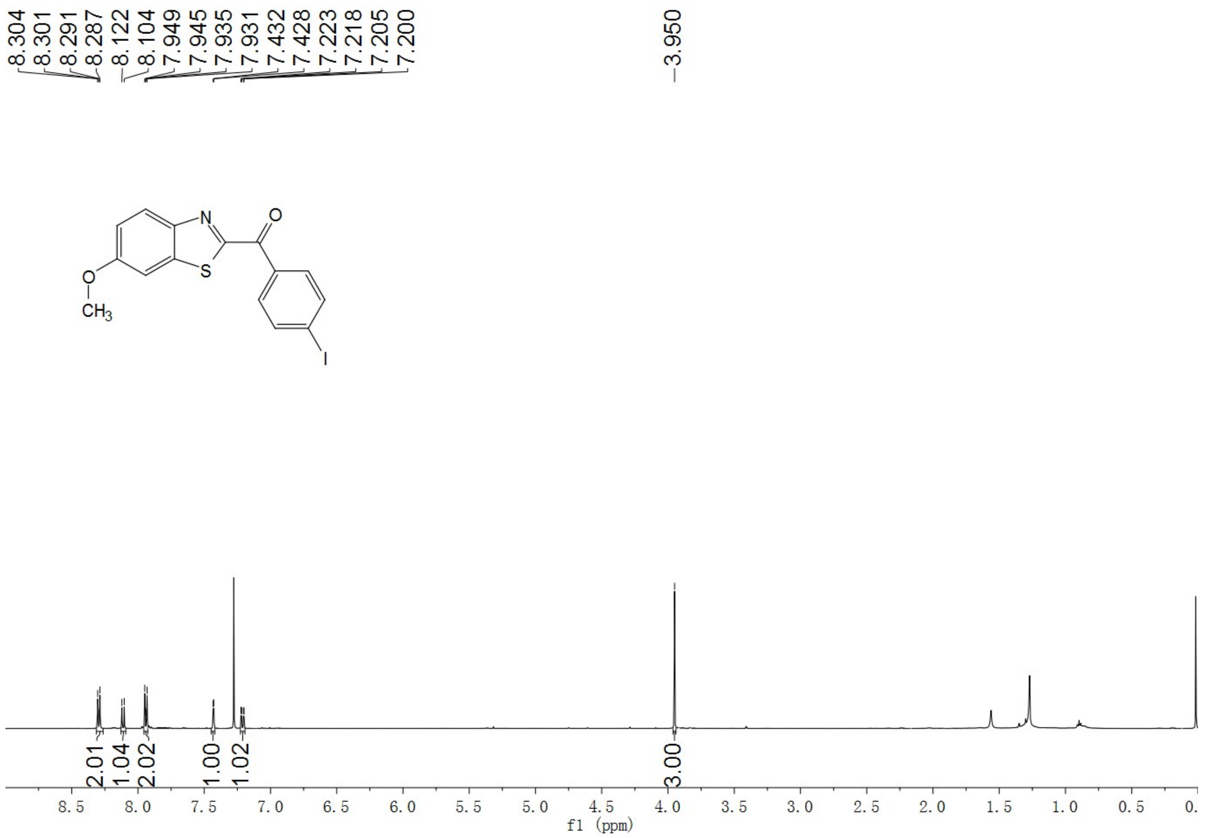

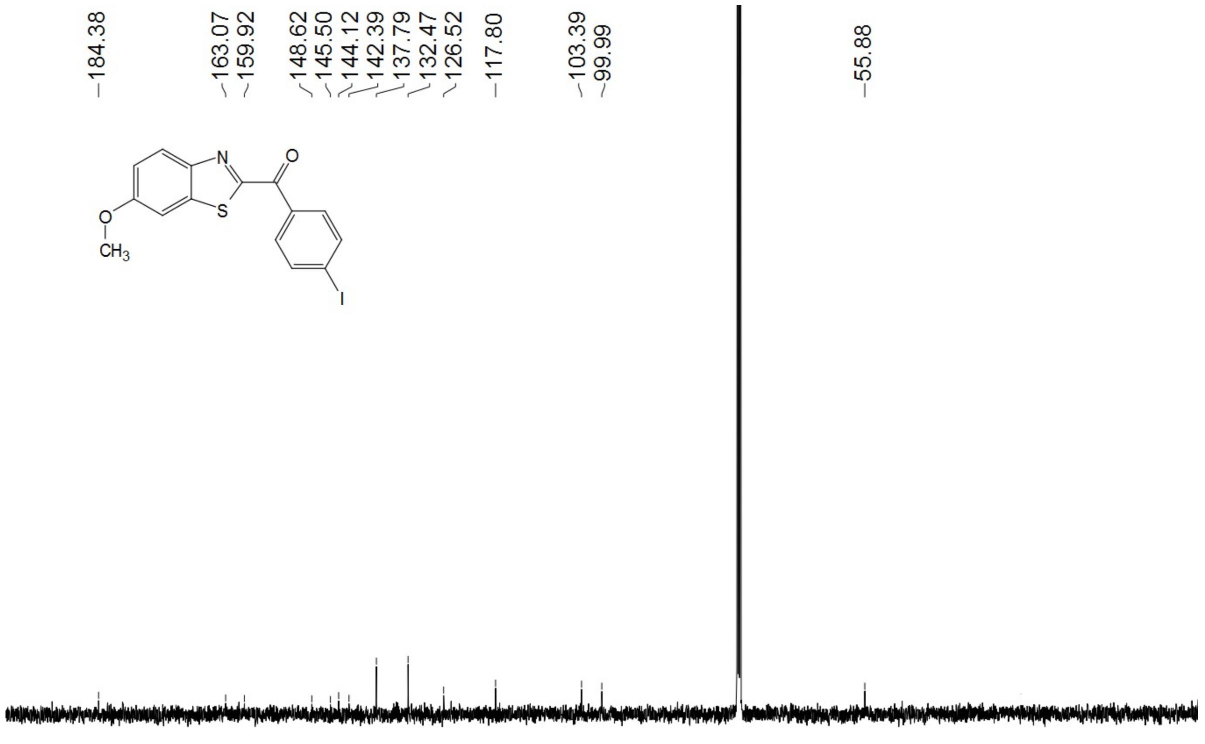

^{1}H and ^{13}C NMR Spectra of 3bg

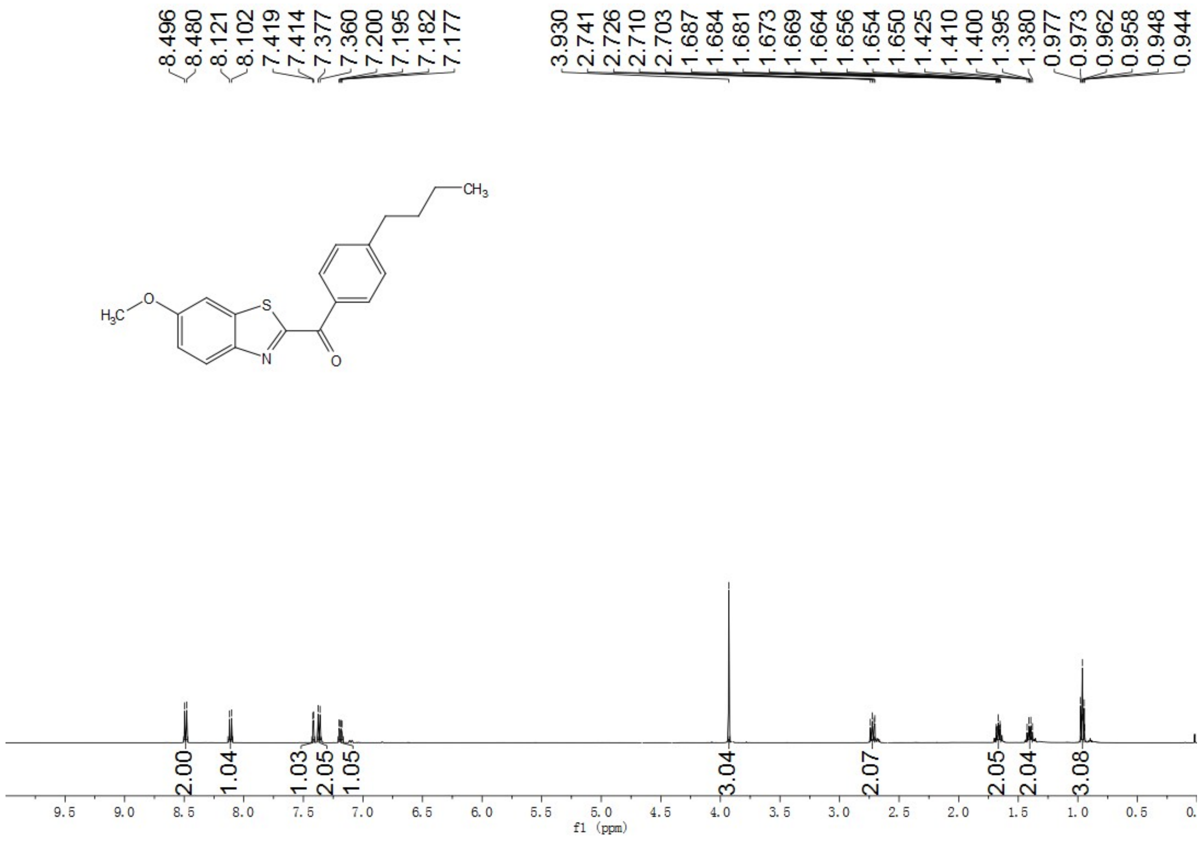

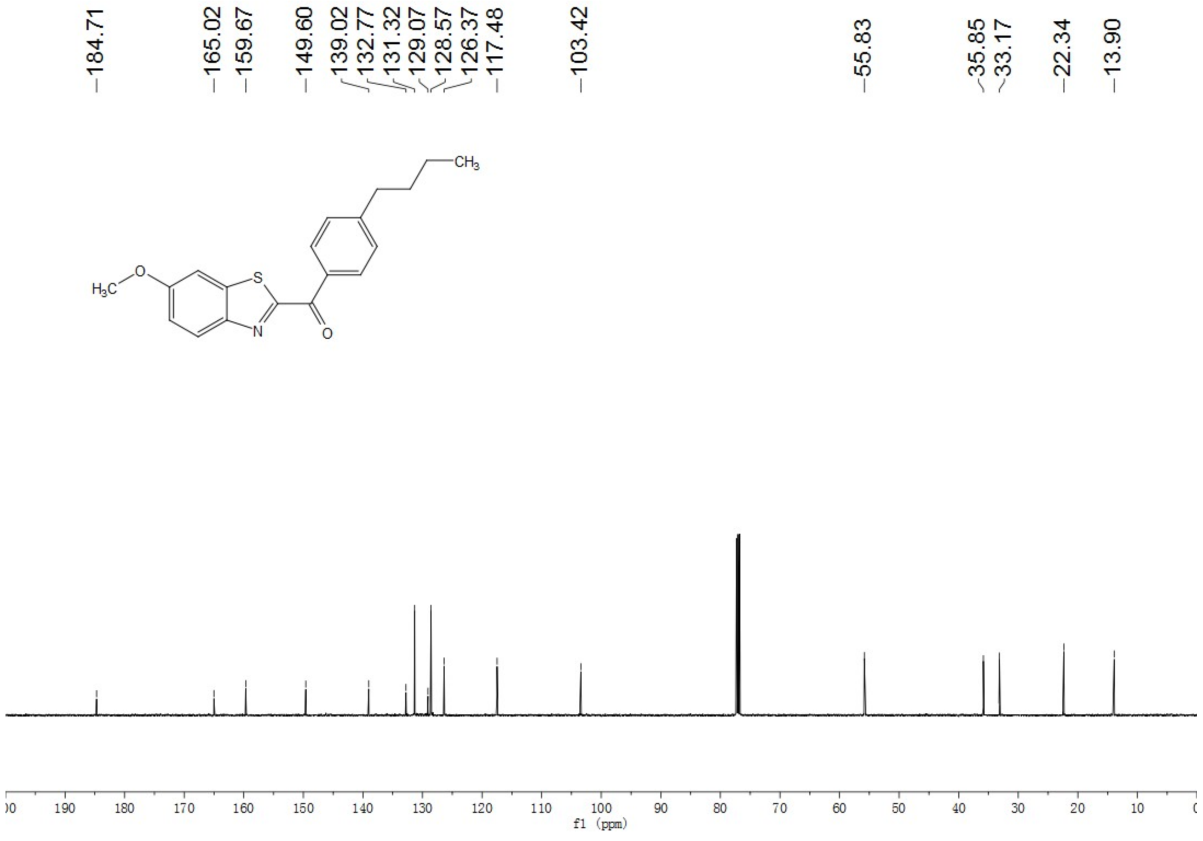

^{1}H and ^{13}C NMR Spectra of 3bh

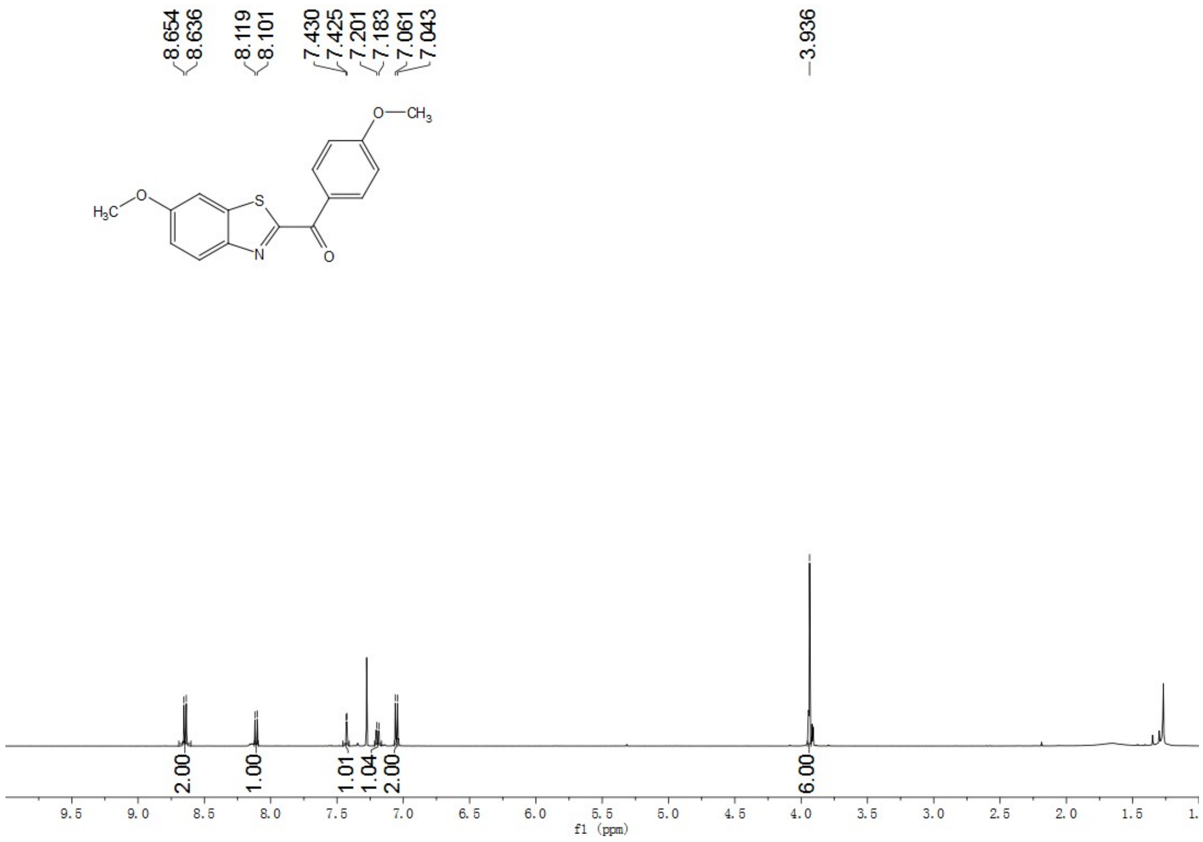

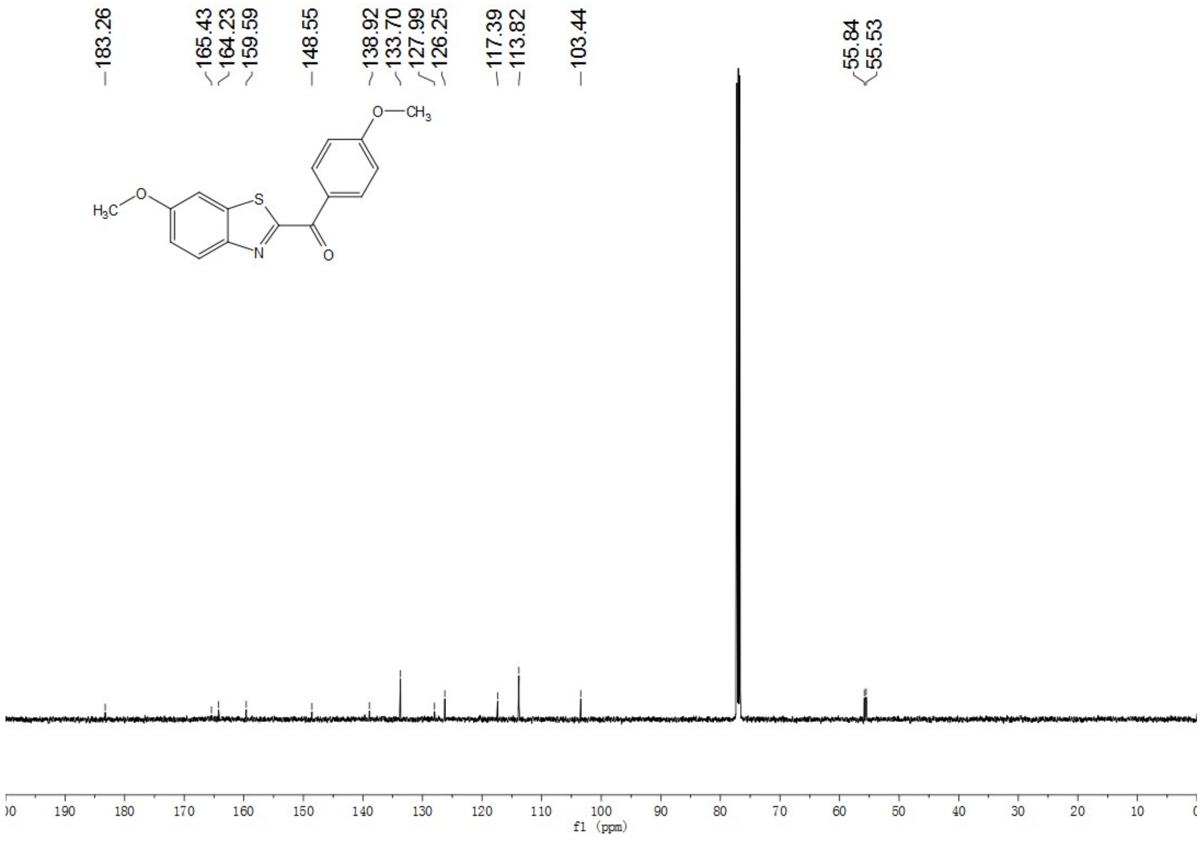

^{1}H and ^{13}C NMR Spectra of 3bi

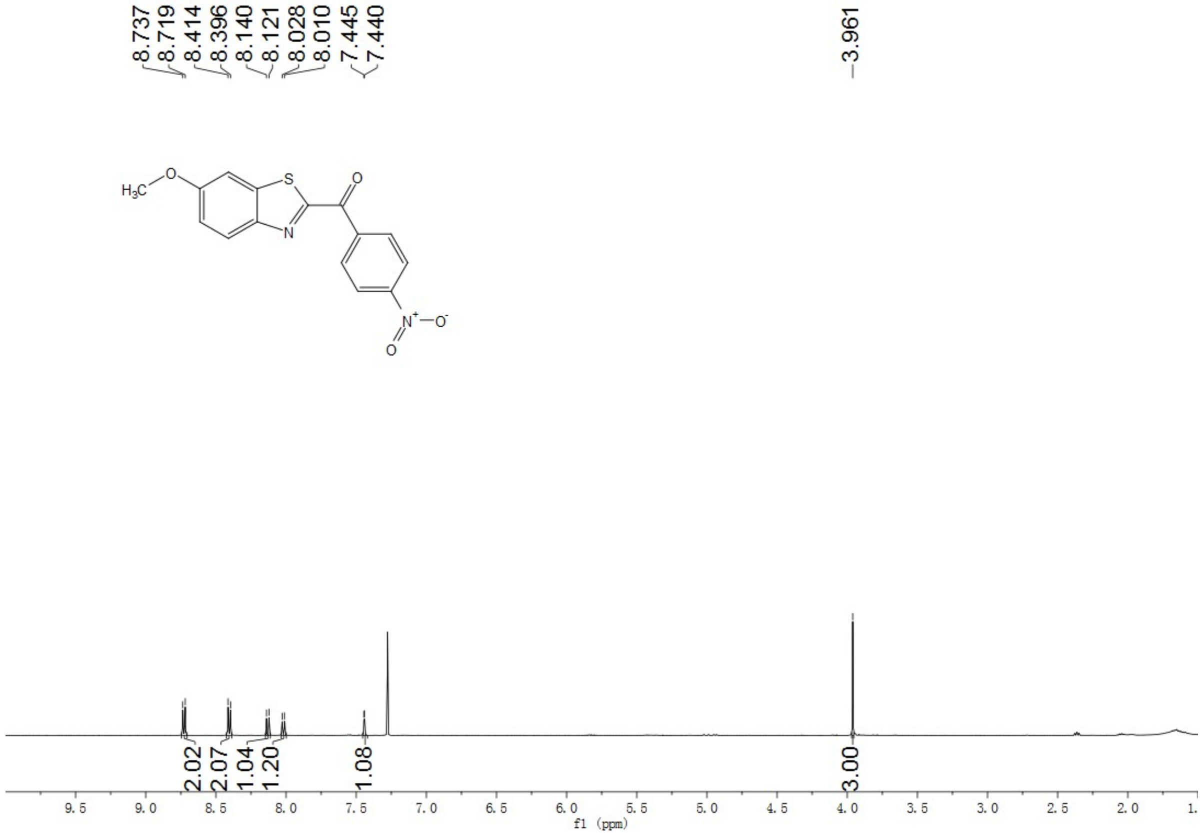

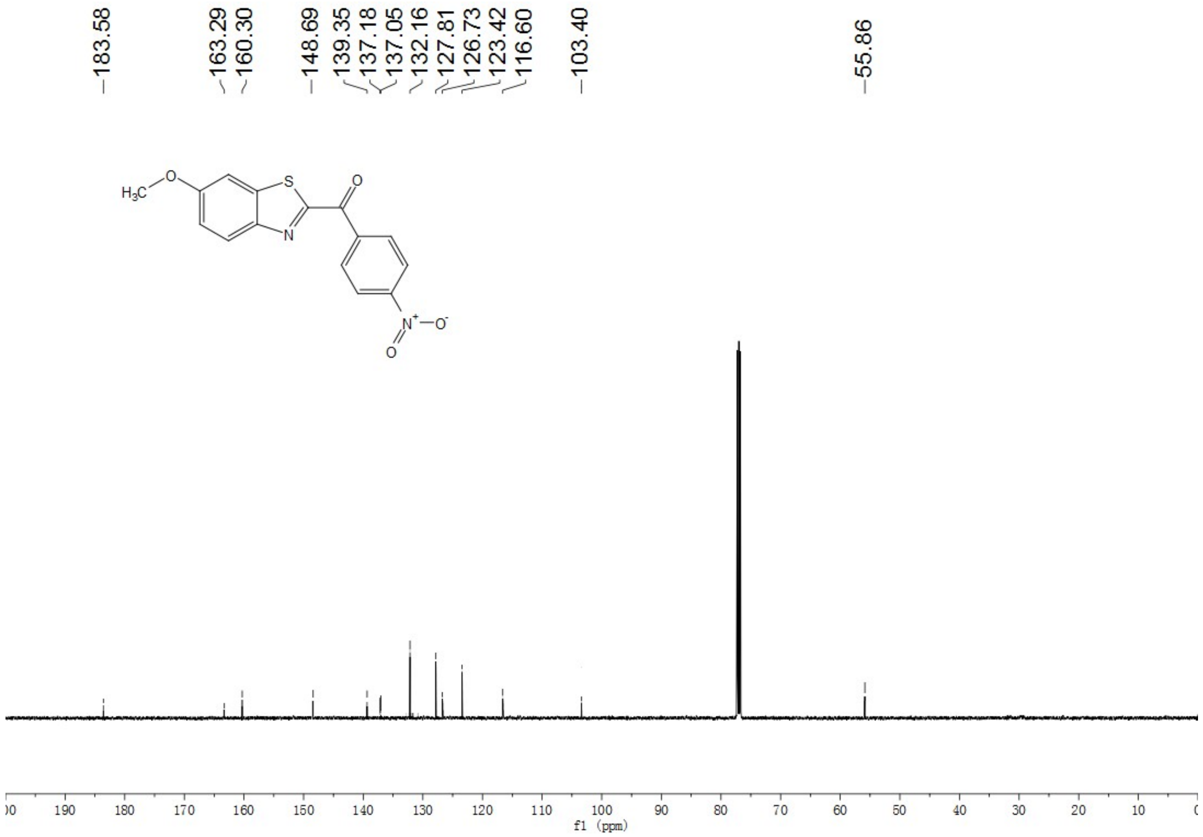

^{1}H and ^{13}C NMR Spectra of 3bj

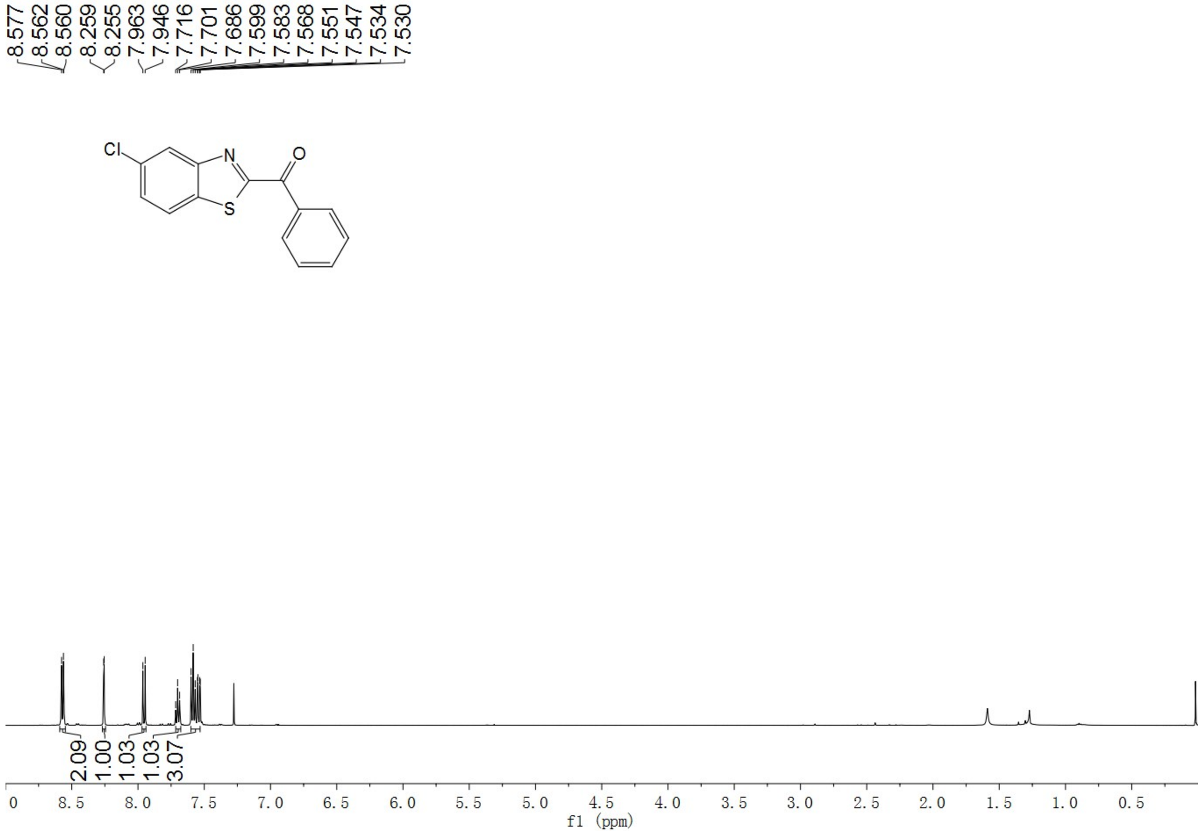

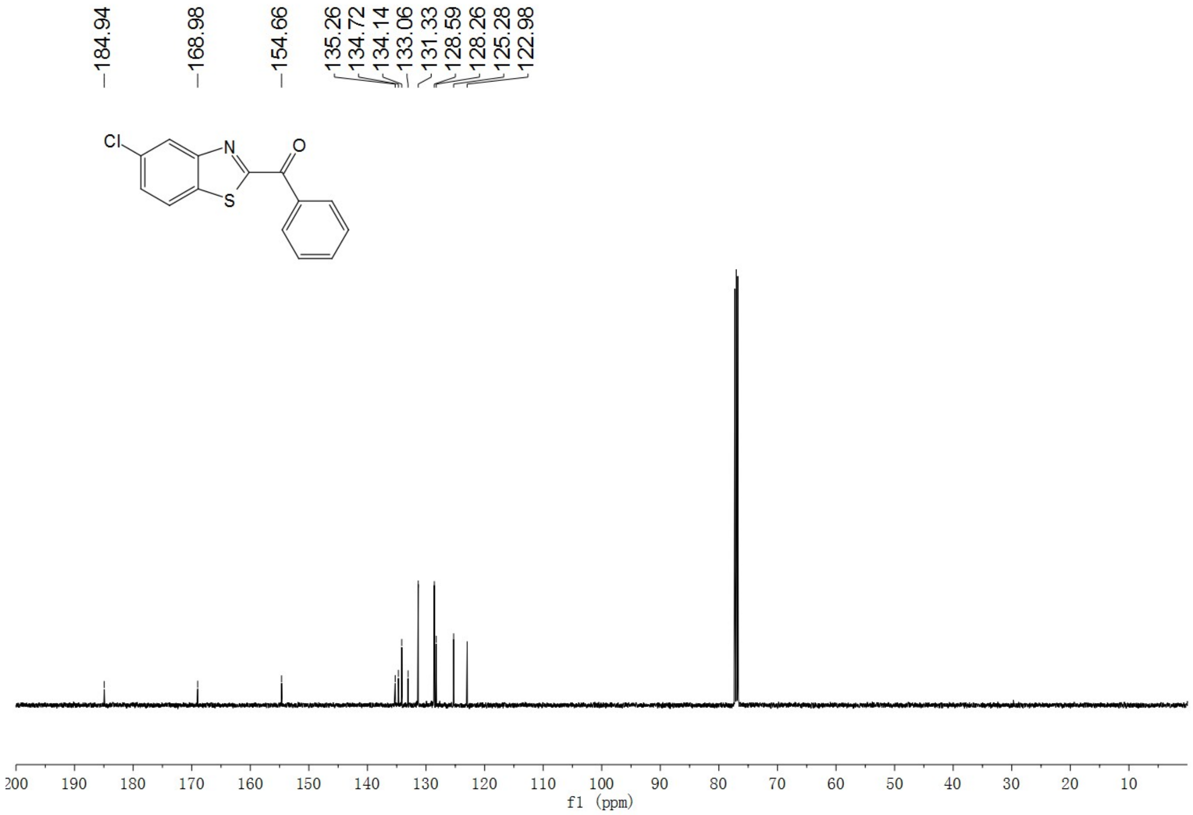

^{1}H and ^{13}C NMR Spectra of 3bk

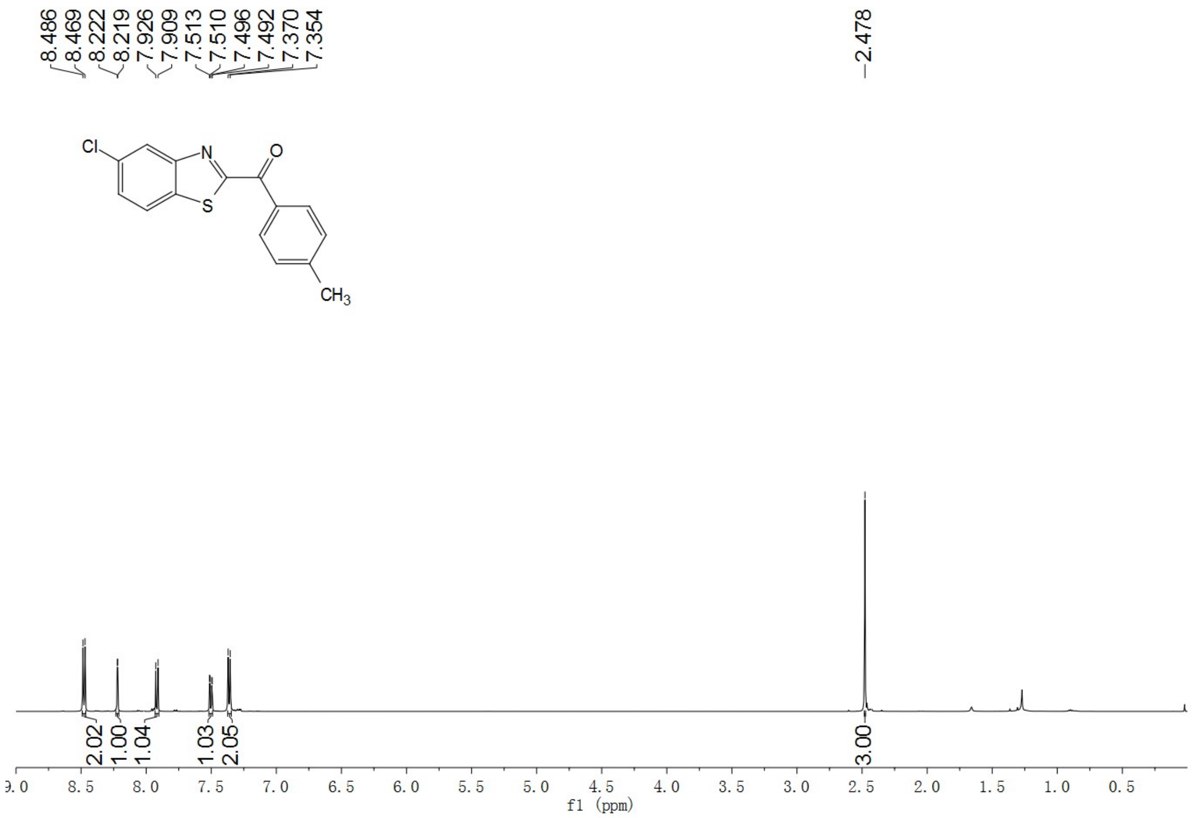

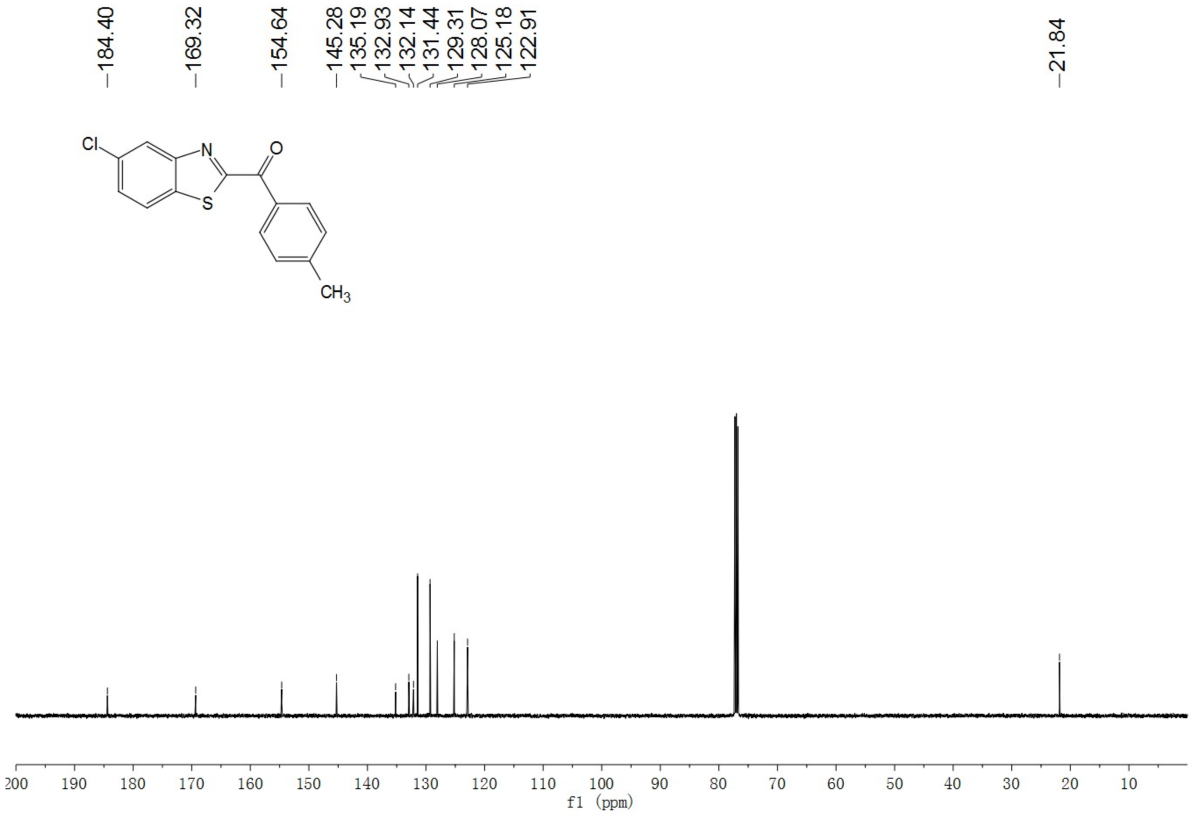

^{1}H and ^{13}C NMR Spectra of 3bl

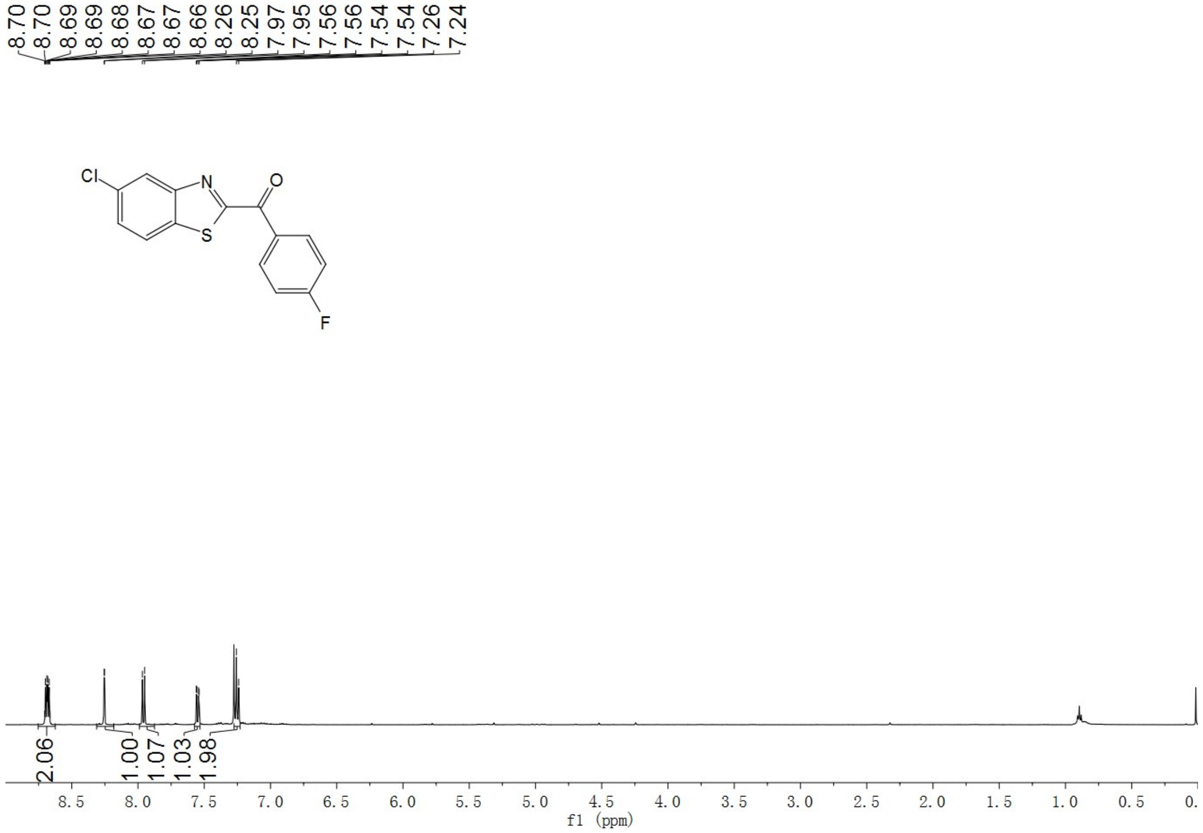

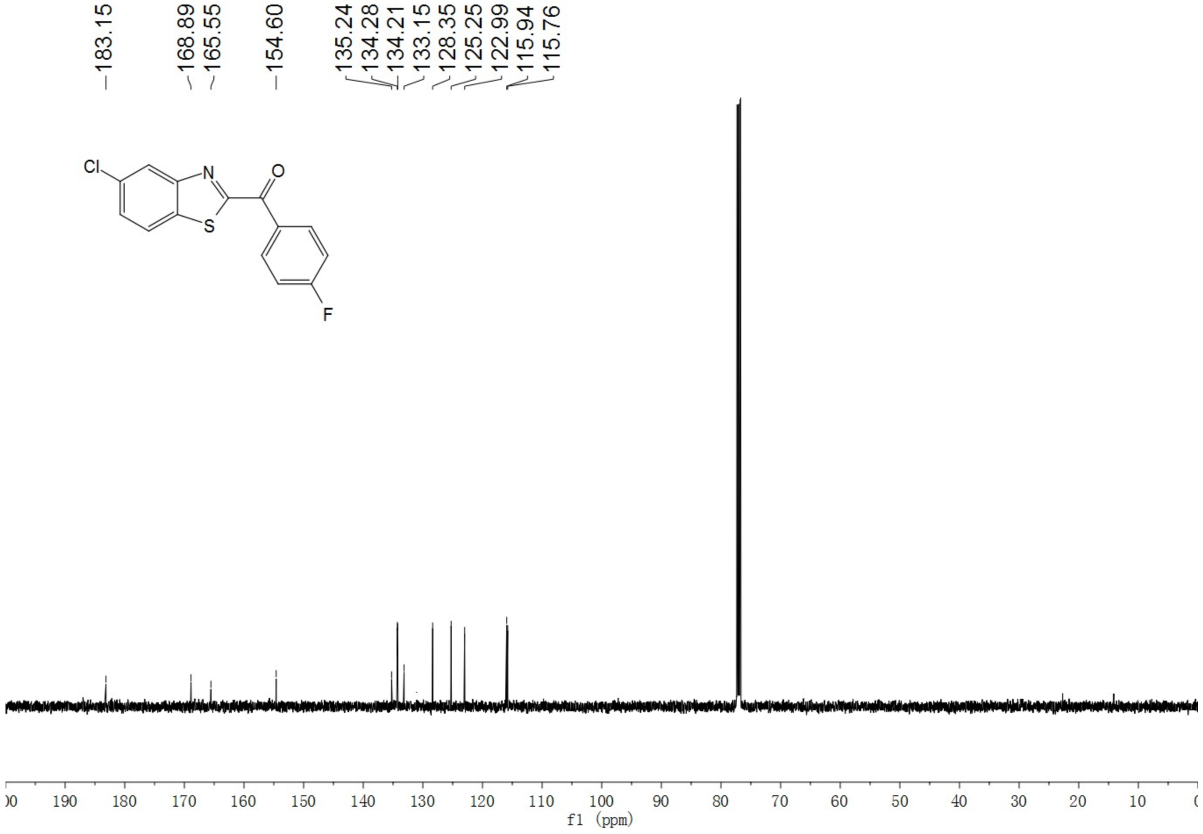

^{1}H and ^{13}C NMR Spectra of 3bm

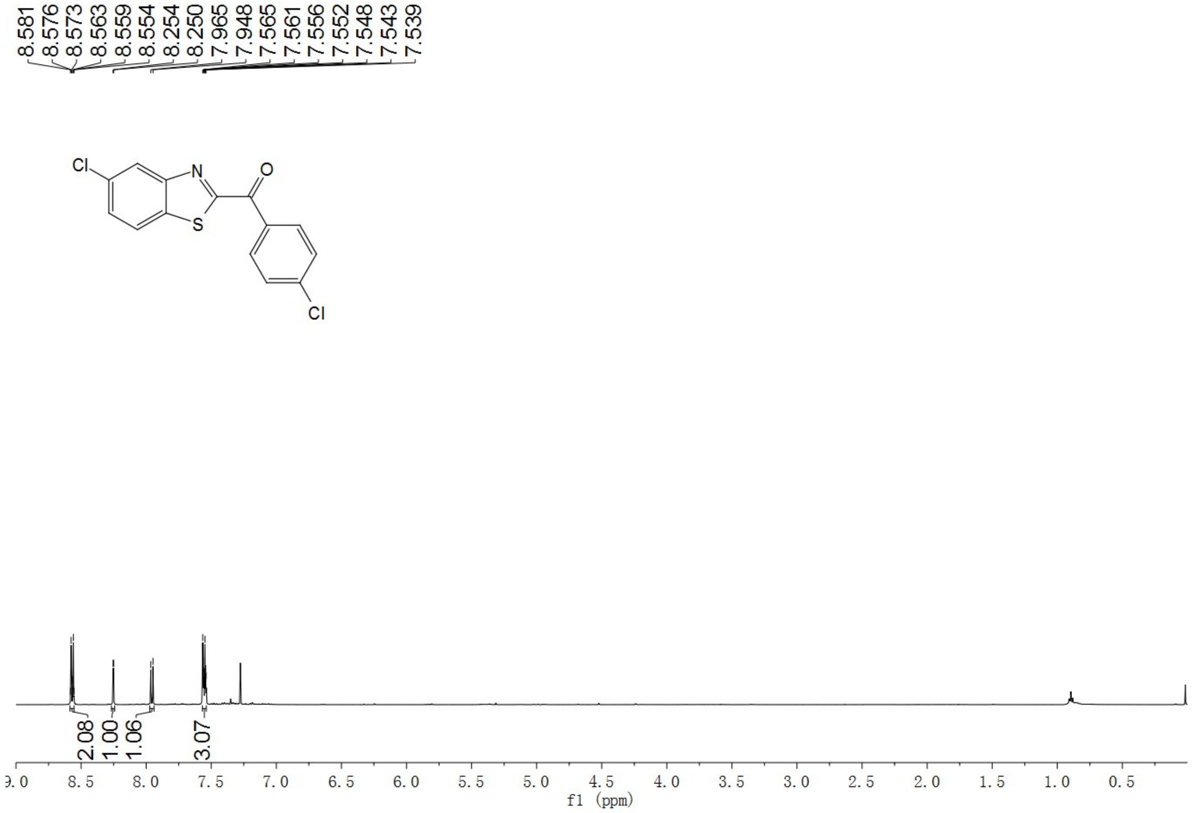

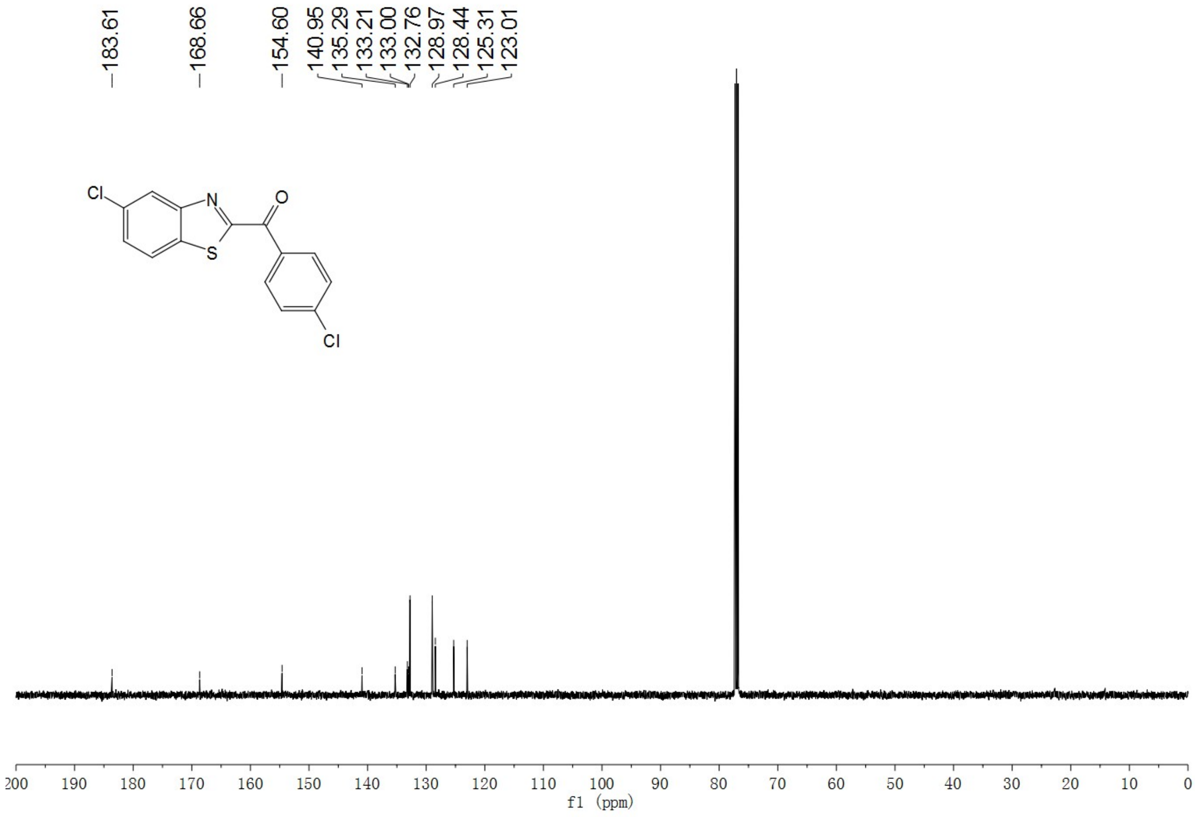

^{1}H and ^{13}C NMR Spectra of 3bn

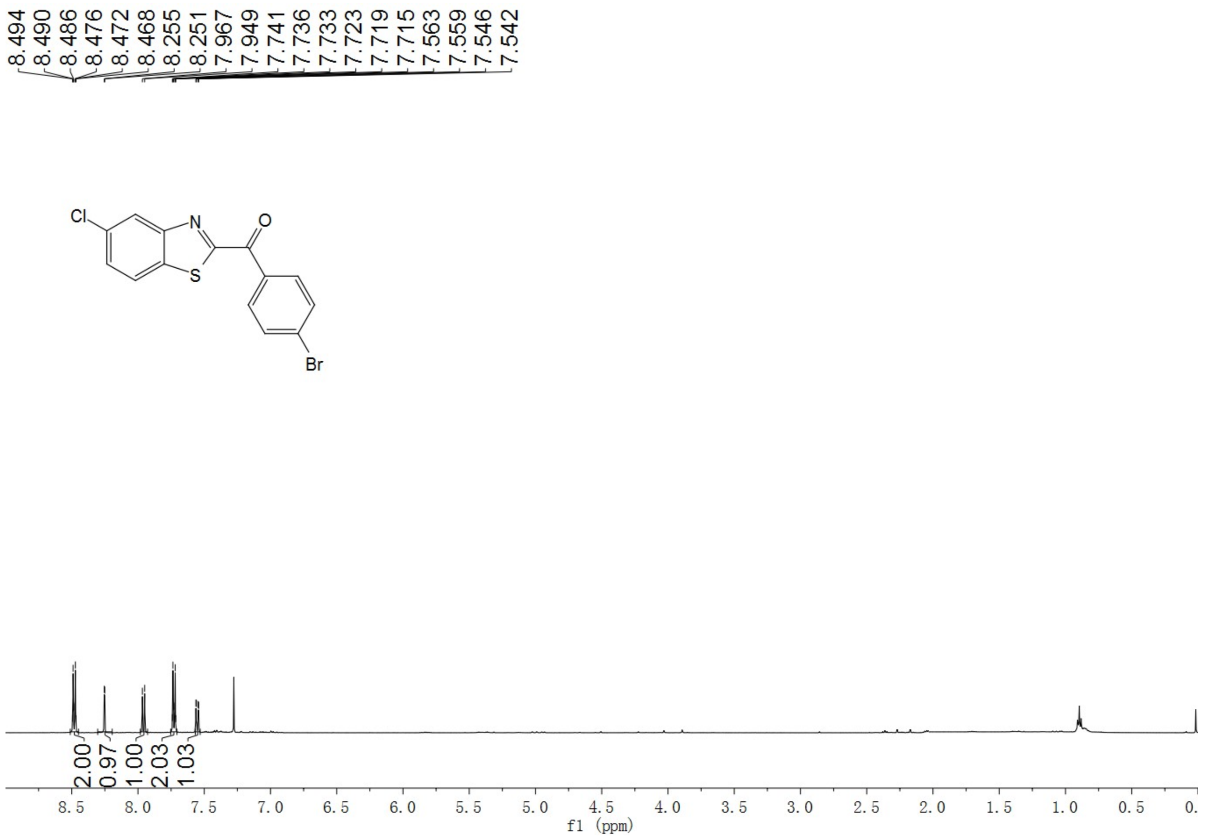

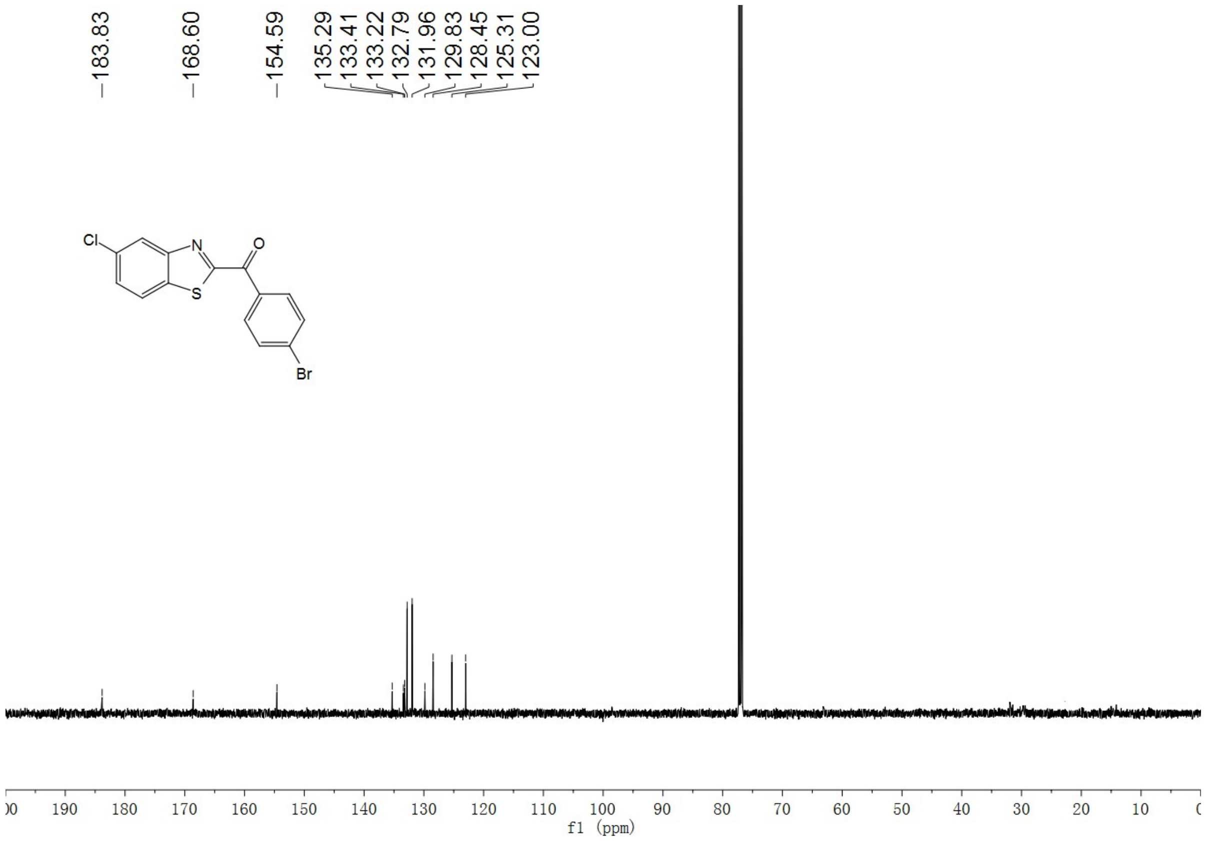

^{1}H and ^{13}C NMR Spectra of 3bo

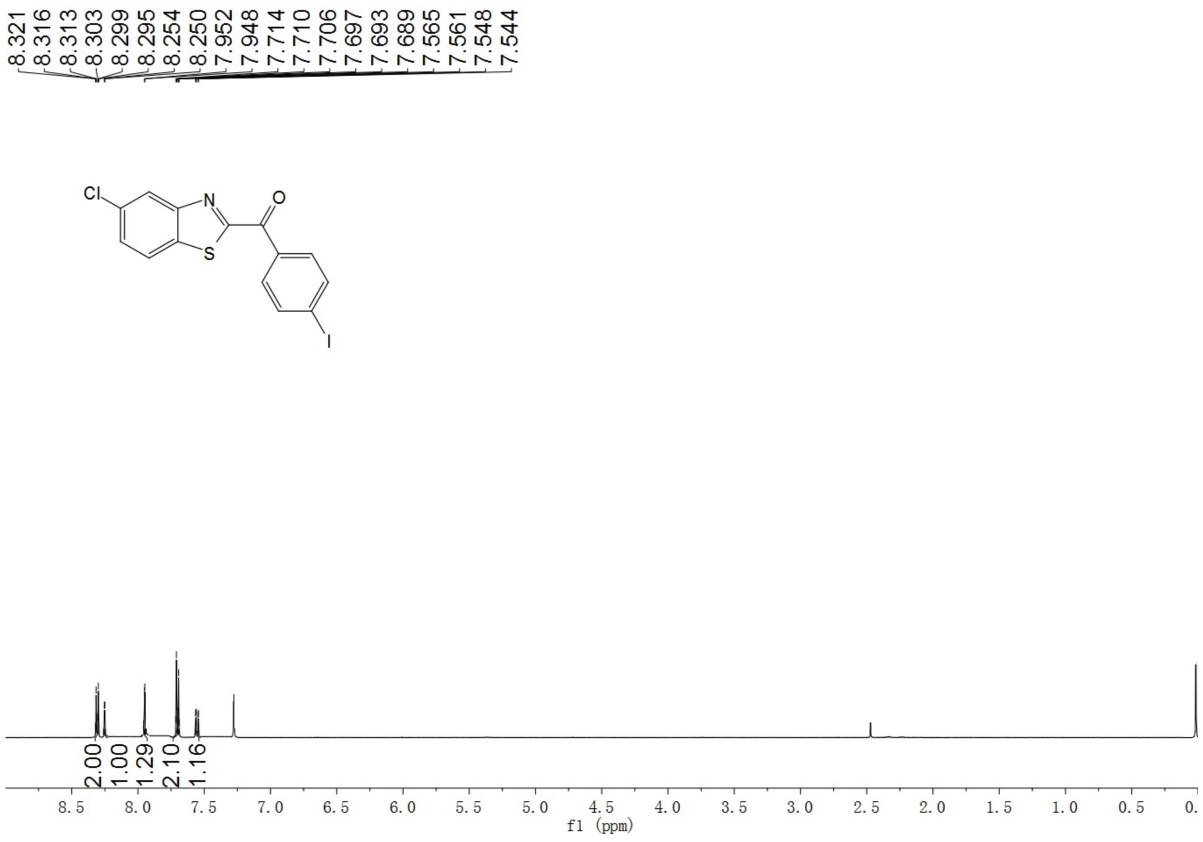

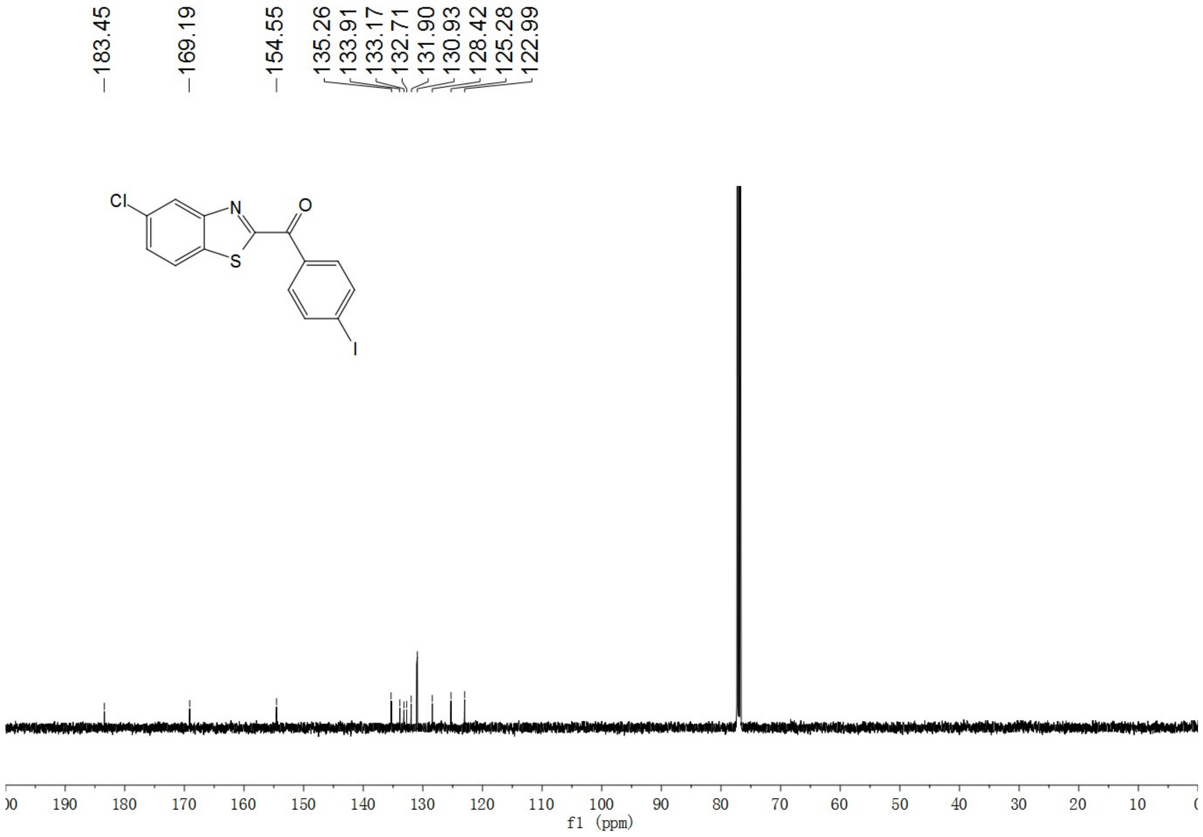

^{1}H and ^{13}C NMR Spectra of 3bp

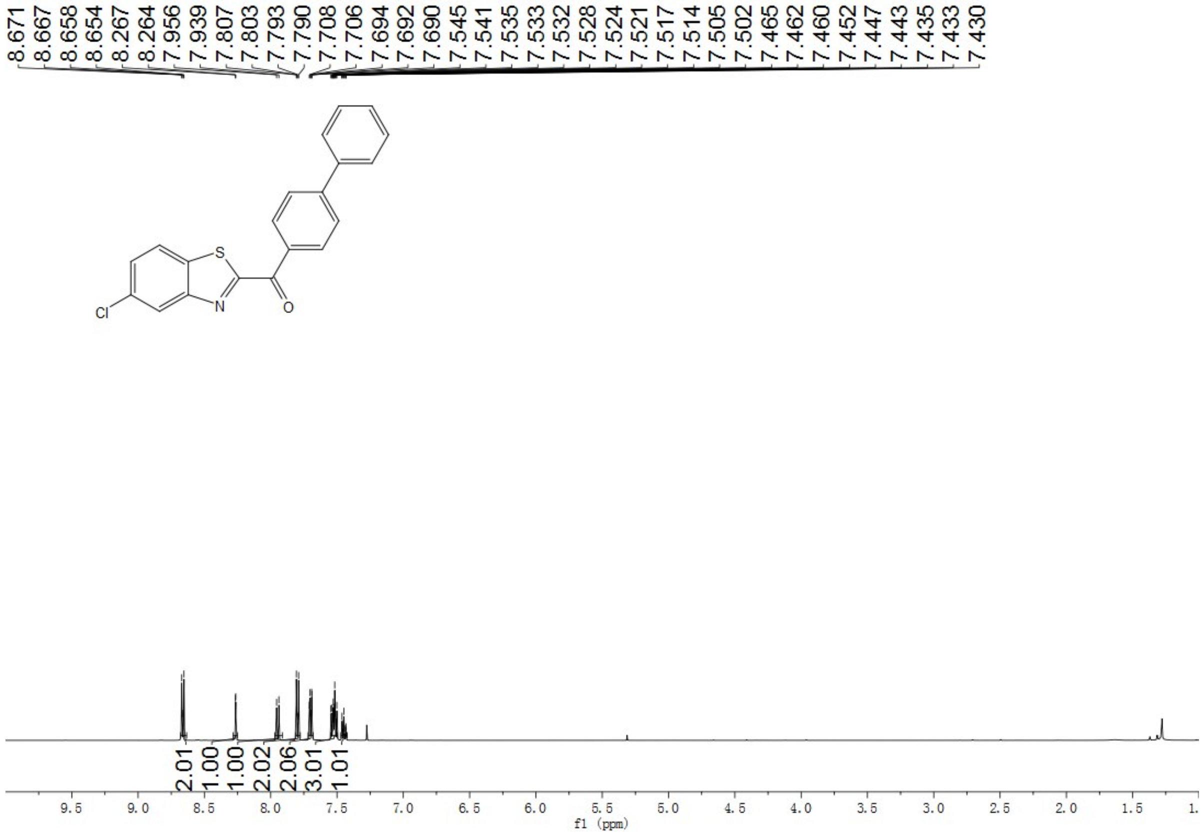

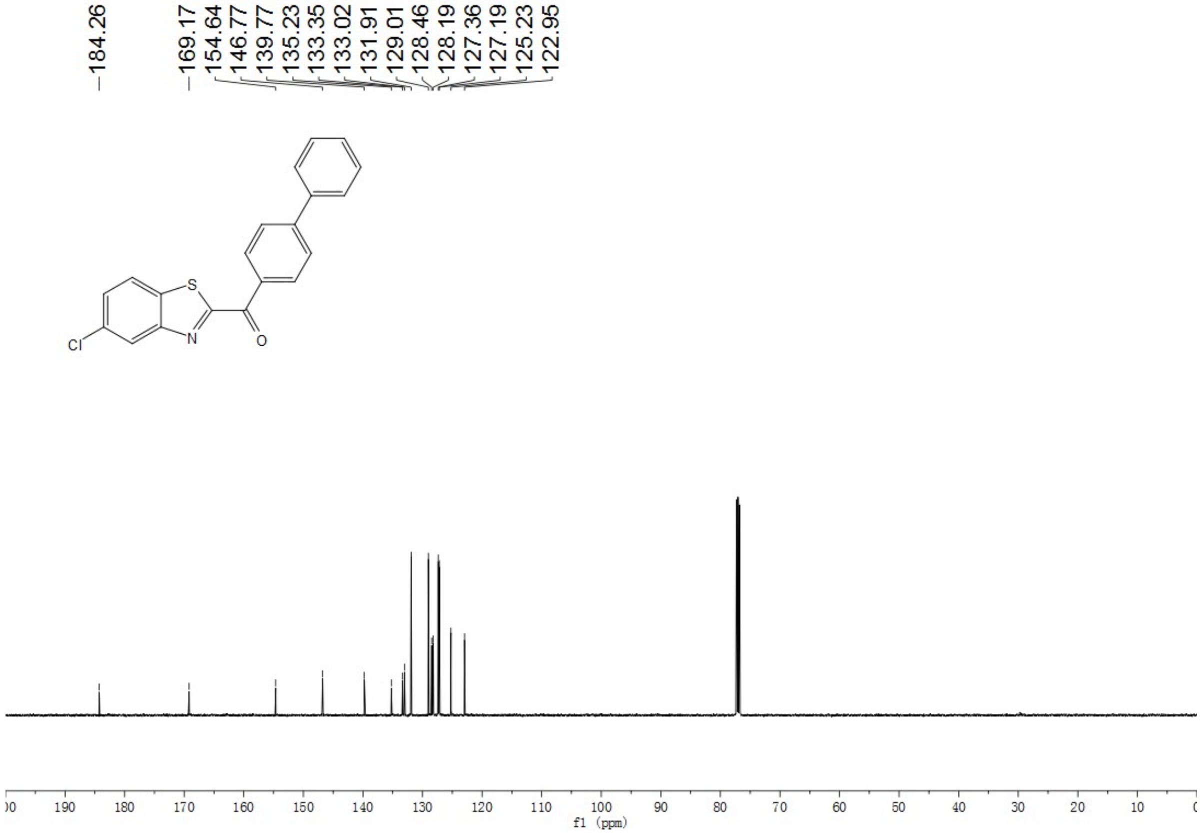

^{1}H and ^{13}C NMR Spectra of 3bq

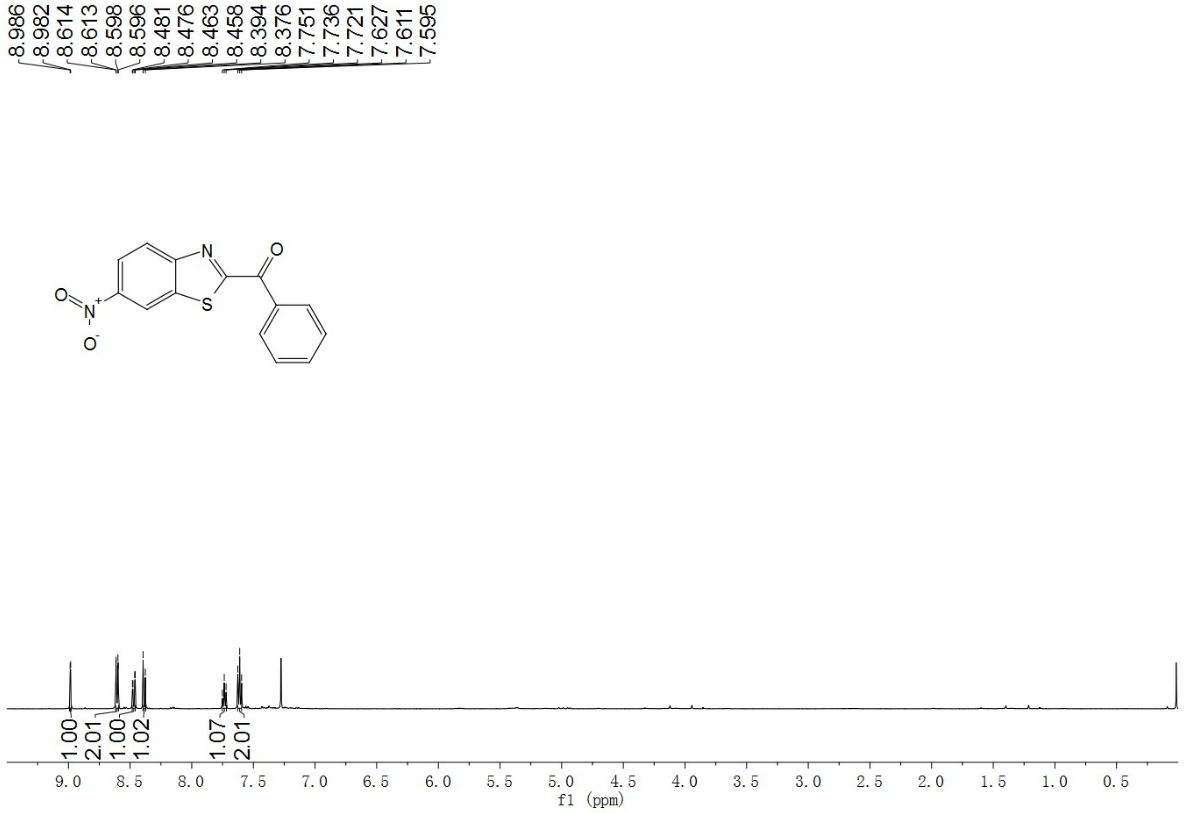

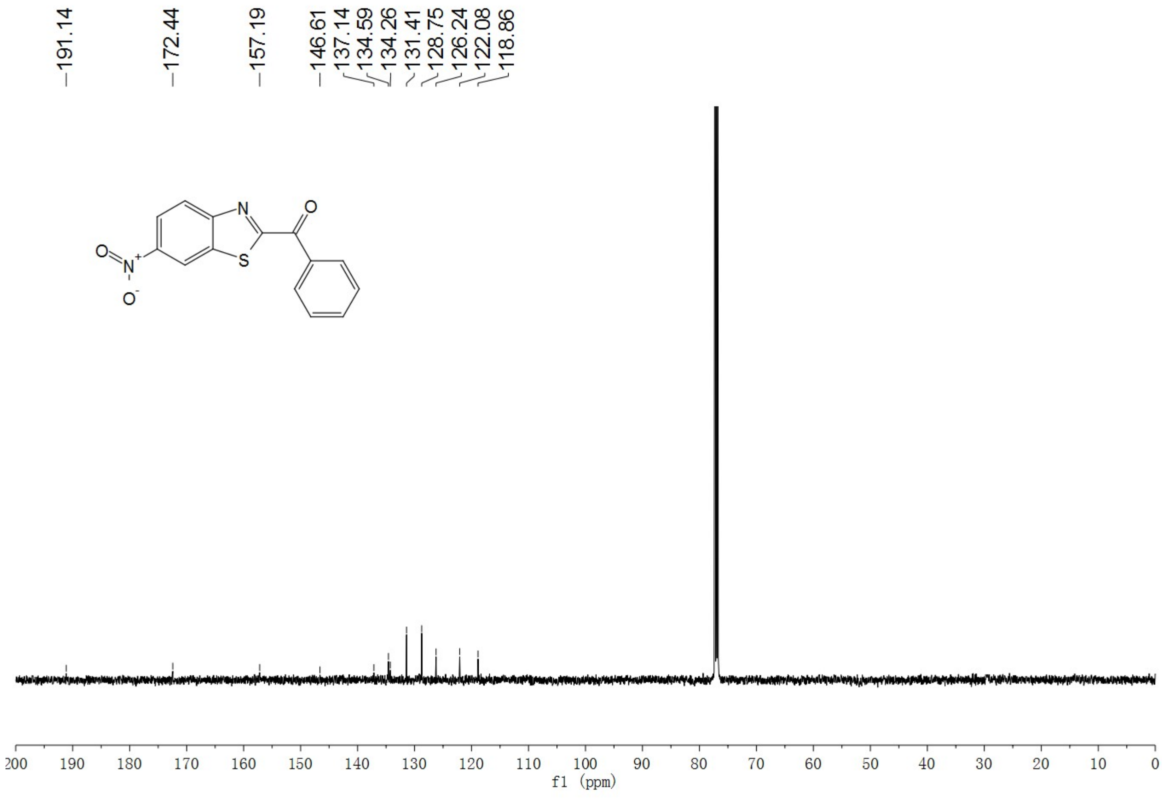

^{1}H and ^{13}C NMR Spectra of 3br

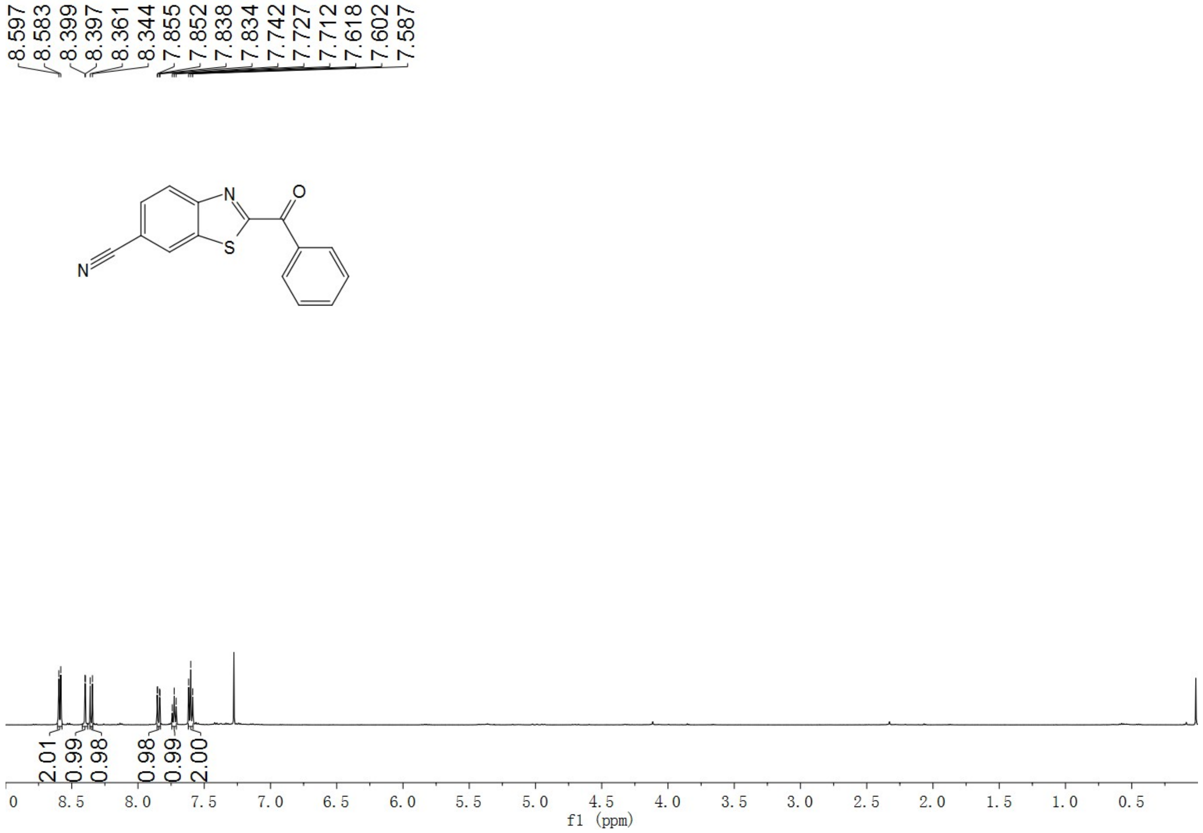

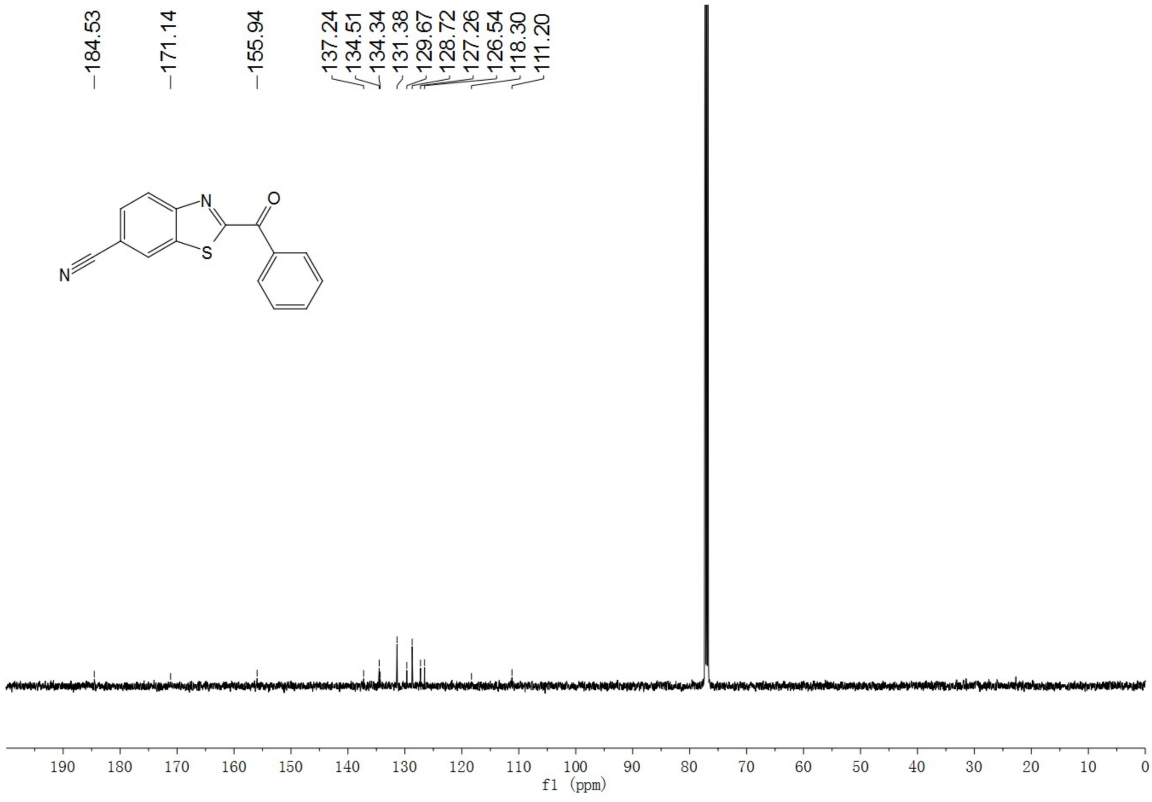

2. NMR Spectrum of 1b

2-Aminobenzenethiol (*1b*): Yellow solid; yield 83% (31 mg); m.p.: 70-71 °C; ^{1}H NMR (500 MHz, cdcl$_3$) δ 7.19 – 7.16 (m, 2H), 6.73 (dd, J = 8.0, 1.0Hz, 1H), 6.61 (td, J = 7.5, 1.5 Hz, 1H), 4.17 (s, 3H); ^{13}C NMR (125 MHz, CDCl$_3$) δ 148.59, 136.83, 131.60, 118.80, 118.28, 115.27.

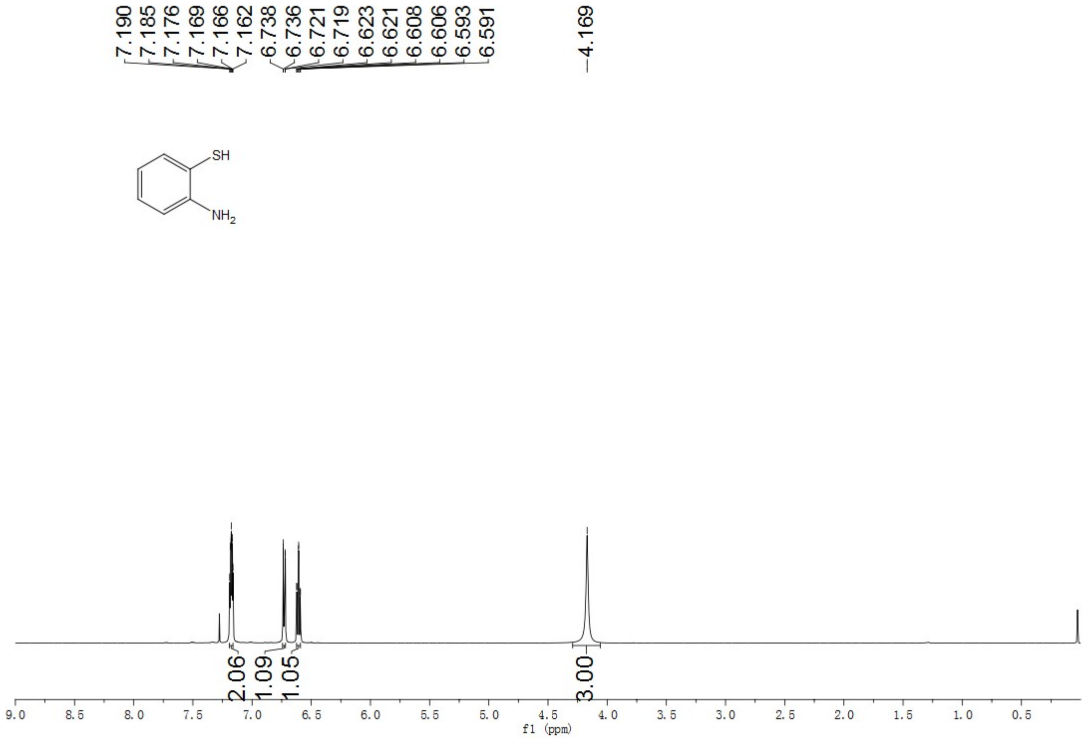

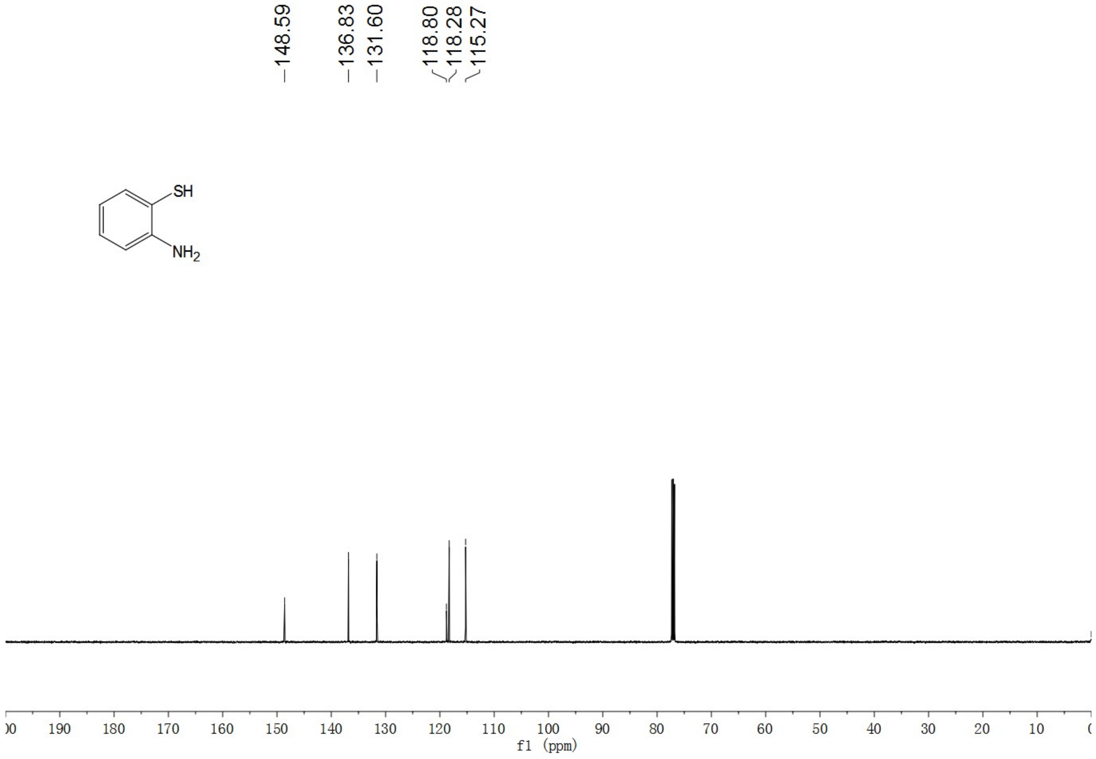

3. GC-MS Spectrum of PhI

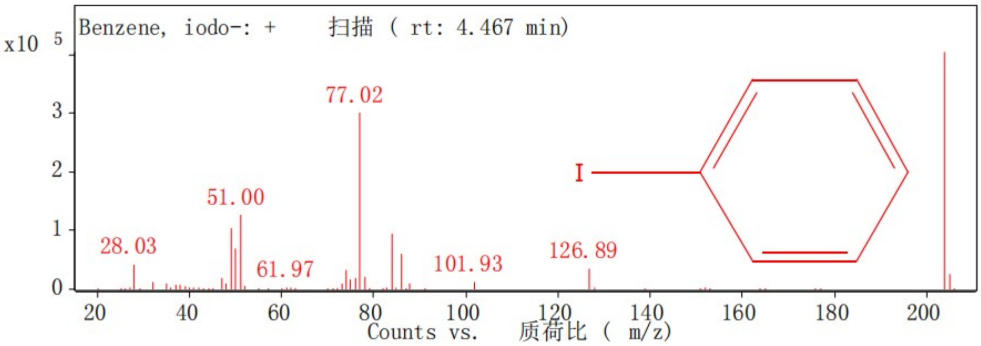

质谱图结构

Benzene, iodo-

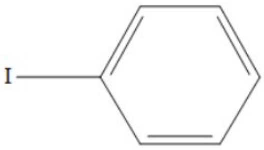

Supplement: Supplementary file 1 [file molecules-27-00726-s001.zip › molecules-1557267-supplementary.pdf]
